# DNA methylation patterns of *FKBP5* regulatory regions in brain and blood of humanized mice and humans

**DOI:** 10.1038/s41380-024-02430-x

**Published:** 2024-02-05

**Authors:** Natan Yusupov, Simone Roeh, Laura Sotillos Elliott, Simon Chang, Srivaishnavi Loganathan, Lidia Urbina-Treviño, Anna S. Fröhlich, Susann Sauer, Maik Ködel, Natalie Matosin, Darina Czamara, Jan M. Deussing, Elisabeth B. Binder

**Affiliations:** 1 Department Genes and Environment, Max Planck Institute of Psychiatry, Munich, Germany; 2 International Max Planck Research School for Translational Psychiatry (IMPRS-TP), Munich, Germany; 3 Molecular Neurogenetics, Max Planck Institute of Psychiatry, Munich, Germany; 4 School of Medical Sciences, Faculty of Medicine and Health, University of Sydney, Sydney, NSW Australia

**Keywords:** Neuroscience, Psychiatric disorders

## Abstract

Humanized mouse models can be used to explore human gene regulatory elements (REs), which frequently lie in non-coding and less conserved genomic regions. Epigenetic modifications of gene REs, also in the context of gene x environment interactions, have not yet been explored in humanized mouse models. We applied high-accuracy measurement of DNA methylation (DNAm) via targeted bisulfite sequencing (HAM-TBS) to investigate DNAm in three tissues/brain regions (blood, prefrontal cortex and hippocampus) of mice carrying the human FK506-binding protein 5 (*FKBP5*) gene, an important candidate gene associated with stress-related psychiatric disorders. We explored DNAm in three functional intronic glucocorticoid-responsive elements (at introns 2, 5, and 7) of *FKBP5* at baseline, in cases of differing genotype (rs1360780 single nucleotide polymorphism), and following application of the synthetic glucocorticoid dexamethasone. We compared DNAm patterns in the humanized mouse (*N* = 58) to those in human peripheral blood (*N* = 447 and *N* = 89) and human postmortem brain prefrontal cortex (*N* = 86). Overall, DNAm patterns in the humanized mouse model seem to recapitulate DNAm patterns observed in human tissue. At baseline, this was to a higher extent in brain tissue. The animal model also recapitulated effects of dexamethasone on DNAm, especially in peripheral blood and to a lesser extent effects of genotype on DNAm. The humanized mouse model could thus assist in reverse translation of human findings in psychiatry that involve genetic and epigenetic regulation in non-coding elements.

## Introduction

Genetic loci associated with risk for psychiatric disorders frequently lie in non-coding regions such as cis-regulatory DNA elements [1, 2]. These gene regulatory elements (REs) have been shown to be enriched for disease-associated genetic variants, but are also targets for epigenetic alterations related to environmental risk exposures [3]. While increasing numbers of risk-associated loci are being cataloged, there remains a large gap in our understanding of their functional consequences on multiple levels, from molecular, to cellular to systems. Human induced pluripotent stem cell- (iPSC) derived systems in combination with gene editing are tools that now allow to assess the functional consequences of both genetic and epigenetic alterations in disease-related gene REs, but they still lack the complexity of an intact organism. This level of exploration requires model organisms, such as rodents, which are established for investigating disease-related neurobiological mechanisms and preclinical testing of pharmacological targets [4]. However, these models are of limited use for the exploration of gene REs relevant to disease due to the lack of sequence similarity with humans in intergenic and gene regulatory regions [5, 6]. While genomic conservation across human and mouse genomes is high overall, only about 40% alignment can be reached at the nucleotide level [6]. The lack of conservation is especially apparent in non-coding regions [6, 7]. To overcome this limitation and allow further mechanistic exploration of disease-relevant human gene REs in whole organisms, genetically engineered humanized mouse models are an option. “Humanized mouse models” refer to mice that carry human genetic sequences, where the mouse gene is substituted by its human orthologue [8–10]. Such models can allow to explore human gene REs that are not conserved in rodents in an intact behaving organism and across all tissues and cell types.

Several humanized mouse models have been applied in neuropsychiatric research. Most of these have targeted coding regions [11–21], while some inserted full-length genes with a potential to model genetic differences lying in non-coding regions [22–30]. However, epigenetic modification of gene REs, also in the context of gene x environment interactions, have not yet been explored in such models. This is the aim of this study, focusing on a widely investigated candidate gene in psychiatric stress research, *FKBP5*, as an example. *FKBP5* encodes a co-chaperone molecule, the FK506-binding protein 5 (FKBP5), a strongly stress-responsive protein that modulates the hypothalamic–pituitary–adrenal (HPA) axis among other targets [31]. Genetic variants in this locus are mainly tagged by the intronic single nucleotide polymorphism (SNP) rs1360780. This SNP has been repeatedly associated with increased risk for a range of psychiatric disorders, mainly in the context of exposure to early adversity [32]. The current mechanistic model derived from human and animal studies proposes that disease risk is mediated by enhanced FKBP5 levels through genetic and epigenetic mechanisms, with downstream, tissue-specific consequences on its many interaction partners [32, 33]. Induction of *FKBP5* transcription by stress/glucocorticoids (GCs) is triggered by binding of the activated glucocorticoid receptors (GR) to specific DNA sequences, so-called glucocorticoid-responsive elements (GREs) [34]. This GC-related induction is moderated by the functional SNP rs1360780 (C/T) which is located close to a GRE in intron 2 of the gene. The minor T allele induces a stronger *FKBP5* expression by generation of an additional TATA-Box binding element to loop back to the transcription start site and is associated with a prolonged systemic cortisol response likely through the effects of FKBP51 on HPA-axis regulation [32, 35]. While this genetic variant has been associated with increased risk for psychiatric disorders, associations mainly occur in the context of early adversity and it has been proposed that the regulatory effects of the SNP need to be accompanied by additional epigenetic changes in other GREs of the *FKBP5* locus that are induced by adversity and stress hormone activation. Demethylation of DNA in cytosine-phosphate-guanine dinucleotides (CpGs) near GREs in introns 2, 5, and 7 of the *FKBP5* gene have been reported following exposure to environmental stressors such as childhood abuse [35] and is likely mediated by direct binding of the GR to GREs [36]. Demethylation of GREs has been associated with subsequent increased transcriptional responsivity of *FKBP5* to GC-stimulation [35]. In summary, it appears that the minor allele of the rs1360780 SNP and the demethylation of DNA at and around GREs are both necessary to increase FKBP5 expression above a disease-relevant threshold ([32] for review). In animal models, increased *FKBP5* activity in specific, mainly limbic, brain regions has been associated with behaviors indicative of increased anxiety and reduced stress coping [37], while blocking of endogenous FKBP51 resulted in opposite behavioral effects [38–40]. In postmortem brain, *FKBP5* expression is higher in patients with schizophrenia and depression, especially in upper layer excitatory neurons [41] and FKBP51 has been proposed as an interesting drug target for a subset of patients [42]. To follow-up on this it would be critical to better understand the epigenetic and genetic regulation of the locus in the context of an organism, which would also improve biomarker development of central *FKBP5* hyperactivity.

Recently, two humanized mouse lines were generated by substituting the murine *Fkbp5* gene by the human *FKBP5* gene differing only in the intronic rs1360780 SNP allele [43]. Nold et al. confirmed that human *FKBP5* is expressed in CNS cells of these mice and that the risk-associated genotype leads to a greater induction of the gene by GCs [44]. It is unclear, however, whether DNA methylation (DNAm) profiles in the relevant intronic GREs would also be recapitulated in the humanized *FKBP5* locus and respond to GR activation in a SNP-dependent way as shown for human cells [36] and how such effects would correlate between brain and blood.

Applying high-accuracy DNAm measurement via targeted bisulfite sequencing (HAM-TBS [45]), we aimed to explore DNAm patterns of CpGs located within three functional intronic GREs of *FKBP5* in three tissues/brain regions: blood, prefrontal cortex (PFC) and hippocampus (HIP) in the humanized *FKBP5* mouse model. We compared DNAm patterns in blood and PFC of the humanized model with data from human cohorts of psychiatric patients and healthy controls (two for blood and one for postmortem brain). Finally, we investigated effects of genotype and of GC-stimulation on DNAm using the GC-analog dexamethasone in the different humanized mouse tissues/brain regions and compared them to effects on DNAm in humans.

## Materials and methods

### Samples

#### Humanized *FKBP5* mouse

**Fig. 1:**
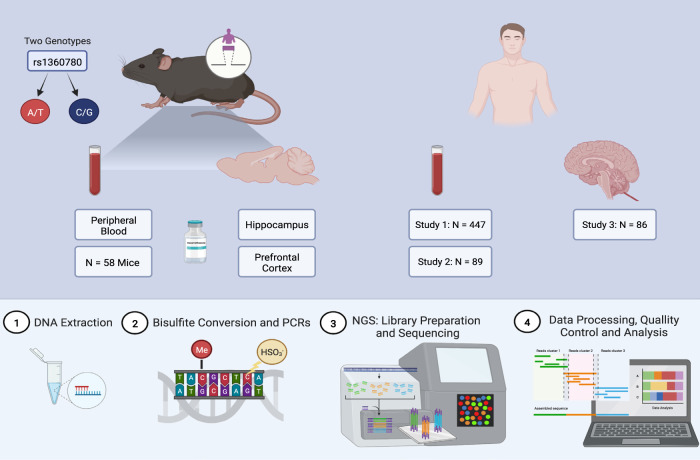
Summary of study design and cohorts. Upper part: Human DNA methylation (DNAm) data was available/generated for three cohorts of different tissues/brain regions: two cohorts of peripheral blood and one of postmortem prefrontal cortex tissue (orbitofrontal cortex, BA 11). Humanized Mouse DNAm data of three tissues: blood, prefrontal cortex, and hippocampus, was available for two humanized mouse lines (carrying different alleles of the rs1360780 SNP) for the *FKBP5* gene. Mice were treated with 2 mg/kg body weight dexamethasone or vehicle and tissue harvested at three time points (baseline, after four and 24 h). Lower part: brief description of high-accuracy measurement of DNAm via targeted bisulfite sequencing (HAM-TBS) preparation and analysis workflow including: DNA extraction, bisulfite conversion of DNA, targeted PCRs amplification and library preparation followed by new generation sequencing, data processing with quality control and subsequent analysis. Created with BioRender.com.

All animal experiments were conducted with the approval of and in accordance with the Guide of the Care and Use of Laboratory Animals of the Government of Upper Bavaria, Germany. Mice were group-housed under standard lab conditions (22 ± 1 °C, 55 ± 5% humidity) and maintained under a 12 h light-dark cycle with food and water *ad libitum*. All experiments were conducted with adult male mice (age: 2–4 months). Generated mice carried either the risk A/T (RiA, C57BL/6NTac-*Fkbp5*^*tm4570(FKBP5)Tac*^) or the resilient C/G (ReG, C57BL/6NTac-*Fkbp5*^tm4571(*FKBP5)Tac^) allele of rs1360780 SNP of the *FKBP5* gene (N_RiA _= 28, N_ReG_ = 30; Fig. 1 and Supplementary Table 1). In these mouse lines the murine *Fkbp5* gene on chromosome 17 (from the start to the stop codon, i.e, exon 2-12 including interspersed introns of ENSMUST00000079413) was substituted by the homologous segment of the human *FKBP5* gene (exon 3–12 of ENST00000536438) (see [43] for a detailed description).

#### Human blood tissue

##### Study 1

We included 447 subjects with and without current (past 4 weeks) psychiatric disorders who consented for the Max Planck Institute of Psychiatry (MPIP) and were recruited in Munich, Germany as participants of two studies: the Biological Classification of Mental Disorders study (BeCOME, registered on ClinicalTrials.gov, TRN: NCT03984084, *N* = 319) [46] and a subset of patients recruited for major depression from a clinical psychotherapy study (OPTIMA, registered on ClinicalTrials.gov, TRN: NCT03287362; *N* = 128) [47] who agreed to participate in an additional biobanking project (see Table 1 and supplementary methods for detailed cohort description). All participants provided written informed consent. The studies and all procedures as well as a specific withdrawal request from the MPIP Biobank were approved by the Ludwig Maximilian University Ethics Committee (application 338-15).

**Table 1:** Demographic details for human cohorts.

	Study 1	Study 2	Study 3
Tissue	Peripheral blood	Peripheral blood	Postmortem brain
Final N	440	89	84
Age (years)			
Mean (SD)	37.6 (13.0)	41.6 (14.0)	52.7 (14.1)
Median [Min, Max]	34 [19, 74]	42 [12, 75]	54 [22, 84]
Sex			
Female N (%)	271 (61.6%)	22 (24.7%)	31 (36.9%)
Current Psychiatric Diagnosis			
Yes N (%)	264 (60%)	59 (66.3%)	51 (60.7%)
Missing N (%)	7 (1.6%)	0 (0%)	0 (0%)
rs1360780 SNP			
CC N (%)	189 (42.9%)	50 (56.2%)	42 (50%)
TC N (%)	154 (35%)	30 (33.7%)	24 (28.6%)
TT N (%)	39 (8.9%)	9 (10.1%)	9 (10.7%)
Missing N (%)	58 (13.2%)	0 (0%)	9 (10.7%)

##### Study 2

We used previously acquired data of DNAm and genotyping for 89 Caucasian participants at our institute (see [36], Table 1 and supplementary methods for detailed cohort description). DNAm levels were determined using the same method (HAM-TBS) as in all cohorts of our study but were available only for introns 5 and 7.

#### Human postmortem brain tissue: study 3

Postmortem brain tissues from the orbitofrontal cortex (BA 11) for 86 subjects were obtained from the NSW Brain Tissue Resource Centre (University of Sydney, Australia). Tissue was dissected from the 3rd 8–10 mm coronal slice of each fresh-frozen hemisphere (see [41], Table 1 and supplementary methods for detailed cohort description). Informed consent for brain autopsy was provided by the donors or their next of kin. The study was approved by the Ludwig Maximilian University Ethics Committee (project 17-085, application 22-0523).

### Experimental design

Mice were treated either with vehicle or 2 mg/kg body weight dexamethasone intraperitoneally and were assessed after four and 24 h (Fig. 1 and Supplementary Table 1 for details of the experimental design). Five mice of each genotype remained untreated and were sacrificed at t0. Three tissues/brain regions of each mouse (blood, PFC, and HIP) were harvested upon sacrifice and stored at −80 °C for further processing.

### Extraction of DNA

#### Humanized mouse

Genomic DNA was extracted from frozen tissue (−80 °C) of the HIP, PFC and submandibular vein blood of all samples except one blood sample (due to blood clot). Prior to DNA extraction, samples of each tissue were randomized (as separate blocks) into two 96-well plates with regards to genotype, time point and treatment using the *Omixer* R package [48].

#### Human blood

Genomic DNA was extracted from blood draw according to standard procedures [36]. Prior to DNA extraction, samples from study 1 were randomized into five 96-well plates with regards to sex, age, childhood maltreatment, and self-reported case-control status using the *Omixer* R package [48].

#### Human postmortem brain tissue

Genomic DNA was extracted from approximately 10 mg fresh-frozen tissue using the QIAamp DNA mini kit (Qiagen, Hilden, Germany) following the manufacturer’s instruction protocol. DNA samples were concentrated using the DNA Clean & Concentrator-5 (Zymo Research, Irvine, CA). DNA concentration was measured using Qubit™ dsDNA BR-Assay (Invitrogen, Carlsbad, California, USA).

### DNA methylation analysis

DNAm at the *FKBP5* locus was assessed with high-accuracy DNAm measurement via targeted bisulfite sequencing (HAM-TBS), a next-generation sequencing method for detection of DNAm changes in specific regions, as described in detail by Roeh et al. [45]. Briefly, triplicates of samples (200-500 ng DNA each processed according to the manufacturer’s instructions) were treated with bisulfite using EZ DNA Methylation Kit (Zymo Research, Irvine, CA). Amplification of target sequences (Supplementary Table 2: primers list; Supplementary Table 3: amplicons list) was performed using TaKaRa EpiTaq HS Polymerase (Clontech, Mountain View, CA; final concentration: 0.025 U/l). Selected bisulfite-specific primers originated from a validated panel of regulatory regions within the *FKBP5* locus (details in [45]). Amplicons were quantified using the Agilent 4200 TapeStation (Agilent Technologies, Waldbronn, Germany) and pooled by Hamilton pipetting robot. To remove excess of primers and genomic DNA, after speed-vacuum and resuspension in 50 µl, a double-size selection using Agencourt AMPure XP beads (Beckman Coulter GmbH, Krefeld, Germany) was performed. Next, PCR-free libraries were prepared with Illumina TruSeq DNA PCR-Free HT Library Prep Kit (Illumina, San Diego, CA) according to the manufacturer’s protocol (500 ng of starting material). Qubit 1.0 (Thermo Fisher Scientific Inc., Schwerte, Germany) was used for quantification of libraries prior to equimolar pooling. Quality assessment of final pooled library was performed with Agilent’s 2100 Bioanalyzer (Agilent Technologies, Waldbronn, Germany) and Kapa Library quantification kit on LightCycler480 (Roche, Mannheim, Germany). Sequencing of libraries was conducted on an Illumina MiSeq with Reagent Kit v3 (Illumina, San Diego, CA; 600 cycles, 12pM Library, paired-end mode, 15% PhiX).

Proportions of blood cell types were calculated from Illumina Infinium MethylationEPIC BeadChip (Illumina, San Diego, CA, USA) data for study 1 (*N* = 436, see details in [49]) and the Illumina Infinium HumanMethylation450 BeadChip for study 2 (*N* = 89, see details in [36]) as suggested by Houseman et al. [50].

### Data processing

The following preprocessing was applied separately to all generated data of humanized mouse, human blood and human postmortem samples. Quality of reads was assessed with *FastQC* [51]. Reads were trimmed with *cutadapt* v1.11 [52], setting the minimal read length to 100 bp. Reads were mapped with *Bismark* v0.18.2 [53] to a restricted reference comprised of the amplicon sequences, including 50 bp padding on each side. Overlapping ends of reads were removed symmetrically to avoid sequence quality dropping towards the end of each read. Data was inserted to R v4.0.4 [54] and underwent further preprocessing steps: (1) exclusion of PCR artifacts, (2) exclusion of samples with low median coverage (low sequencing depth) in every amplicon (total read number <1000, humanized mouse: *N* = 3; human blood: *N* = 30, human postmortem brain: none), (3) exclusion of samples with low rates of bisulfite conversion (<95%, none), and (4) failed amplicons: sufficient coverage in more than 50% of the samples in all amplicons. Raw methylation calling and bisulfite conversion assessment were performed by *methylKit* R package v1.6.3 [55], with minimum Phred quality score of 30 (99.9% base call accuracy). After QC, a total of 20 CpGs in introns 2, 5, and 7 of the *FKBP5* locus shared by the three data sets remained. CpGs were named after their positions on chromosome 6 of the human reference genome hg19 (Supplementary Table 4: list of CpGs with genomics locations). Next, we excluded technical outlier samples per amplicon (DNAm < (1. quartile – 2xIQR) or > (3. quartile + 2xIQR) in over 50% of CpGs; humanized mouse: PFC: *N* = 1, Blood: *N* = 1 and HIP: *N* = 5; human blood: *N* = 24; human postmortem brain: *N* = 6). One animal was excluded due to hydrocephalus, leaving 57 subjects in the final cohort. Seven human blood and two human postmortem samples were removed due to technical issues (blood: two failed library preparation, one pipetting error, four had missing CpGs > 20%; brain: missing CpGs > 20%, outlier in DNA isolation batch). Final samples comprised 440 and 84 subjects for human blood and postmortem brain, respectively. To exclude major sources of variation explained by technical batch effects, DNAm data of each data set (in the humanized mouse each tissue separately) was dimensionality reduced via principal component analysis after imputation using the *missMDA* R package v1.18 [56]. Subsequently principal components were tested with ANOVA of linear models for possible batch effects (humanized mouse: row, column, plate and dissector (in brain tissues); human blood: row, column, plate and isolation batch; human postmortem brain: row, column, isolation batch, hemisphere, brain pH, postmortem interval and storage time). Batch effects of column and dissector were detected in brain tissue of the humanized mouse and included as covariates in all statistical models. The same procedure revealed batch effects of storage time and brain pH in human postmortem brain as well as isolation batch and plate in human blood. The covariates were included in all statistical models. Batch-corrected data was used for visualized comparison of means between tissues/brain regions and species as well as correlation analysis (corrected with ComBat of the sva R package v3.38.0 [57]).

### Genotyping of human postmortem prefrontal cortex

Genotyping was conducted using Illumina global screening arrays (GSA-24v3-0, Illumina, San Diego, CA, US) excluding SNPs with low call rate (98%), a minor allele frequency <1% or deviation from Hardy-Weinberg-Equilibrium (*p*-value < 1 × 10^-05^). Individuals with call rates <98% were excluded. Only unrelated individuals were included for further analysis. After LD-pruning, outliers on multi-dimensional scaling components of the genotypes IBS matrix (>3 standard deviations (SD) from the mean on any of the first 10 axes) and heterozygosity outliers (>3 SD from mean heterozygosity) were removed. Allelic information for the rs1360780 SNP was retrieved. Complete data with genotype and DNA methylation was available for 75 subjects.

### Statistical analysis

Mean and SD (in percent) were used for comparison and Spearman correlations were used to analyze similarities in DNAm levels between tissues/brain regions. To evaluate genotype and dexamethasone treatment effects on DNAm of the humanized mouse, multiple linear regressions were performed on M transformed values (M = log2(Beta/(1-Beta)) [58]). Normality of values was evaluated using quantile-quantile plots. Prior to regression modeling, non-variable CpGs (interquartile range, IQR < 1% methylation) within a tissue were removed (Blood and PFC: *N* = 2; HIP: *N* = 4; Supplementary Fig. 1). The following linear model was used: CpG Methylation ~ significant batch covariates (if present) + genotype + treatment + genotype × treatment. *P*-values were FDR corrected for multiple testing. *P*-values, *q*-values, beta estimates, standard error and F-statistic are reported. Even considering removed samples, a power analysis (G*Power version 3.1.9.6 [59]) showed a sufficient power (>0.8) for detecting medium main effects (Cohen’s *f*^2^ = 0.2) at a significance criterion of *α* = 0.05 in the multiple linear regression. All statistical analyses were conducted in R version 4.0.4 [54].

## Results

### Similarity of DNA methylation levels in relevant intronic *FKBP5* GREs in blood, prefrontal cortex and hippocampus of humanized *FKBP5* mice

**Fig. 2:**
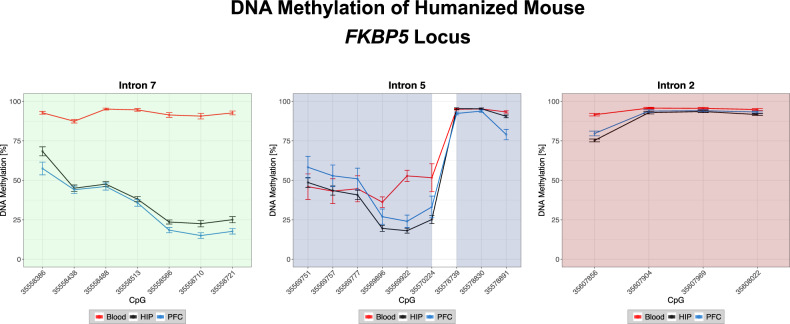
DNA methylation levels in peripheral blood, prefrontal cortex and hippocampus of humanized mouse. Depicted are DNAm levels of 20 CpGs (mean and standard deviation in percent) of three introns of the humanized *FKBP5* gene (green: intron 7, blue: intron 5, red: intron 2) across three tissues at baseline (*N* = 10). CpGs are named according to their positions on the human reference genome hg19.

Twenty CpGs (named according to positions on the human reference genome hg19) within six amplicons of the humanized *FKBP5* locus, covering the three main intronic GREs (Fig. 2), were investigated in 57 animals. CpGs of intron 7 showed similar DNAm patterns between both brain regions (5% average of delta mean DNAm) and these differed strongly from blood (−59% for PFC and −53% for HIP average of delta mean DNAm). CpGs in intron 2, however, were similar across all analyzed tissues (5% average of delta mean DNAm; Fig. 2; Supplementary Table 5: baseline mean and SD of DNAm; Supplementary Table 6: delta mean DNAm across tissues/brain regions). Intron 5 showed more diverse DNAm patterns with CpGs where DNAm in blood was similar to HIP and PFC (delta mean DNAm in 35569751, 35569757, 35569777, 35578739 and 35578830 of <4% for HIP and <12% for PFC) and CpGs with different DNAm levels in all three tissues (35569896, 35569922, 35570224, 35578891; Fig. 2; Supplementary Table 5, 6). Overall, we observed low correlations of DNAm levels across CpGs in brain and blood of the humanized mouse with only very few correlations reaching significance (Supplementary Fig. 2; Supplementary Table 7). This was also reflected in different inter-CpG correlation structure of blood and the two brain regions, with the latter being more similar (but still showing relevant differences in correlation structure as compared to blood (Supplementary Fig. 3)).

To compare brain-blood correlations of CpGs from our humanized mice to correlations previously reported in humans, we used two publicly available tools from studies that have evaluated correlations of CpG DNAm between blood and several brain regions in humans using a genome-wide DNAm array (Illumina Infinium HumanMethylation450 BeadChip): Blood Brain DNA Methylation Comparison Tool (https://epigenetics.essex.ac.uk/bloodbrain/ [60]) and Blood-Brain Epigenetic Concordance (BECon, https://redgar598.shinyapps.io/BECon/ [61]). However, due to the limited representation of our CpGs within the *FKBP5* gene on the array, only one CpG from our panel was present: 35570224 in intron 5 (cg14284211). Similar to the humanized mouse model (Blood-PFC: *r*_s_ = 0.09, *N* = 30; Blood-HIP: *r*_s_ = 0.1, *N* = 29), there were low correlations between blood and different brain regions in the Blood Brain DNA Methylation Comparison Tool (blood-PFC: *r*_s_ = -0.009, *N* = 74; blood-entorhinal cortex: *r*_s_ = −0.006, *N* = 71; blood-superior temporal gyrus: *r*_s_ = −0.11, *N* = 75 and blood-cerebellum: *r*_s_ = −0.027, *N* = 71) but higher correlations were reported in BECon (blood-BA10: *r*_s_ = 0.41; blood-BA20: *r*_s_ = 0.15; blood-BA7: *r*_s_ = 0.27, *N* = 16). Overall, 31 CpGs within the *FKBP5* locus have been assessed in the Blood Brain DNA Methylation Comparison Tool but only two showed significant correlations (cg06087101 and cg08915438). Our analysis in humanized *FKBP5* animals with tissues ascertained at the same time thus supports low correlation between DNAm of blood and brain tissue in this locus, especially considering that DNAm seems to be well recapitulated in humanized mice as described below.

### Comparison of DNA methylation levels in blood and prefrontal cortex of humanized *FKBP5* mouse and humans

We next compared baseline DNAm levels of the ascertained REs (introns 2, 5, and 7) at the *FKBP5* locus between humanized mouse and human tissue. We used previously generated DNAm data of peripheral blood ([36] for details, study 2) and two newly generated human data sets (study 1 for blood and study 3 for PFC) using HAM-TBS technology. Study 2 did not include CpGs of intron 2, but was otherwise comparable. Only non- or vehicle-treated animals were considered for this analysis (*N* = 31). In the two human datasets with peripheral blood, DNAm did not differ significantly between datasets after regressing out effects of age, sex, and cell type proportions, indicating consistent DNAm pattern in this locus in the same tissue across different cohorts and measurement batches (Supplementary Table 8: comparison of means; Supplementary Table 9: mean and SD of DNAm).

**Fig. 3:**
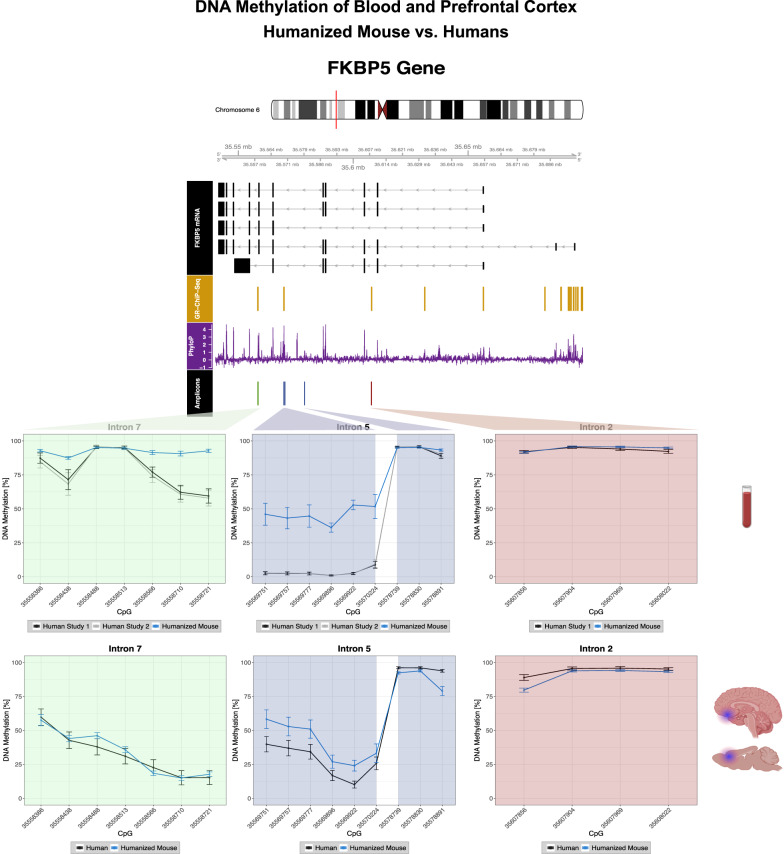
Comparison of DNA methylation patterns in peripheral blood and prefrontal cortex between humanized mouse and human. Depicted are the *FKBP5* human locus on chromosome 6 (hg19, 35541362-35656719), the common human splicing variants of the gene, the genomic locations of glucocorticoid-responsive elements, regions with transcription factor binding derived from ChiP-Sequencing experiments in two cell lines (A549, ECC-1) from the ENCODE project available at the UCSC browser (https://genome.ucsc.edu/, laboratory of Richard Myers, HAIB, Huntsville, Alabama) and PhyloP basewise conservation score available at the UCSC browser (https://genome.ucsc.edu/). Finally, genomic locations (named according to their positions on the human reference genome hg19) and DNAm levels of 20 CpGs in three introns of the *FKBP5* gene (green: intron 7, blue: intron 5, red: intron 2) are displayed as mean and standard deviation (in percent) in three cohorts for blood (upper part; study 1, study 2 and the humanized mouse) and in two cohorts for prefrontal cortex (lower part; study 3 and the humanized mouse). Blood DNAm data was not available for CpGs in intron 2 in study 2, but was otherwise comparable. Regions further away from each other in intron 5 are separated by a white space. GR-ChIP = glucocorticoid-receptor chromatin immunoprecipitation, CpG = cytosine-phosphate-guanine-dinucleotides. Symbols were created with BioRender.com.

**Fig. 4:**
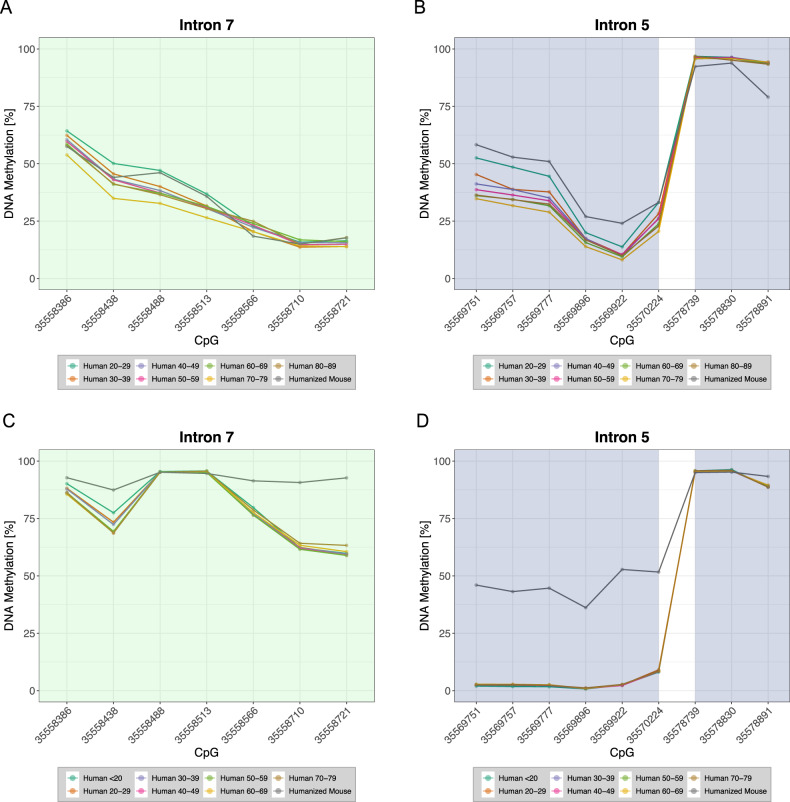
Age-dependent DNA methylation patterns in peripheral blood and prefrontal cortex between humanized mouse and human. Depicted are mean DNAm levels of 16 CpGs (in percent) of two introns of the *FKBP5* gene (green: intron 7, blue: intron 5) for prefrontal cortex (**A**, **B**; study 3 and the humanized mouse) and for blood (**C**, **D**; study 1 and the humanized mouse). Human subjects are categorized into age bins in years (N of subjects in each bin for study 1: <20: 6, 20–29: 158, 30–39: 103, 40–49: 63, 50–59: 84, 60–69: 22, 70–79: 4; N of subjects in each bin for study 3: 20–29: 3, 30–39: 12, 40–49: 17, 50–59: 27, 60–69: 16, 70–79: 5, 80–89: 4). CpGs are named according to their positions on the human reference genome hg19.

Overall, we observed similar DNAm patterns between humanized mouse and humans in blood in all CpGs of intron 2 (<3% delta mean DNAm), and some within introns 5 (35578739, 35578830, 35578891; <5% delta mean DNAm) and 7 (35558386, 35558488, 35558513; <9% delta mean DNAm), but stronger divergence in the other CpGs of intron 5 and 7 (delta mean DNAm range of 35-51% and 14-35% respectively; Fig. 3; Supplementary Table 10: delta mean DNAm of blood). We did not find any significant differences in DNAm pattern in human blood (cohort 1) or in brain due to current disease status after regressing out age, sex and calculated cell-types from both blood and brain (Supplementary Fig. 4). In fact, small differences dependent on depression status were only seen in cohort 2 for one CpGs site also tested in the humanized mouse model but with less than 2.2% DNAm difference at baseline [36]. DNAm patterns in human postmortem PFC showed higher similarity to DNAm of humanized mouse PFC (Fig. 3; Supplementary Table 11: baseline DNAm mean and SD; Supplementary Table 12: delta mean DNAm of PFC). Introns 7 and 2 presented highly similar patterns (mean and SD of delta mean DNAm: intron 7: mean 2%, SD 4%; intron 2: mean 4%, SD 4%). While intron 5 presented somewhat lower similarity at the DNAm levels (mean and SD of delta mean DNAm: part 1: mean 14%, SD 4%; part 2: mean −7%, SD 7%), the pattern of DNAm was highly similar (Fig. 3). Moreover, if comparing to a similar human age group (20–29 years [62]), even more similar DNAm levels were observed in the PFC (Fig. 4 and Supplementary Table 13).

### Genotype and dexamethasone effects on DNA methylation in humanized *FKBP5* mouse

To investigate genotype, dexamethasone, and genotype-specific glucocorticoid-induced effects on DNAm in the different tissues/brain regions (blood, PFC, and HIP), we administered 2 mg/kg dexamethasone (or vehicle) intraperitoneally to the two humanized mouse models and measured DNAm levels after four and 24 h. After removing CpGs with low variability within each tissue/brain region (IQR < 1%), we performed multiple linear regression models with 18 CpGs in blood and PFC and 16 CpGs in HIP.

Regarding genotype, the risk-associated allele of rs1360780 SNP was associated with significantly lower DNAm levels at ten CpGs after FDR correction in blood at baseline (Supplementary Table 14). The strongest effects were observed in four CpGs of both introns 5 and 7 (Supplementary Fig. 5A). In the human data, decreased DNAm with the T allele was also observed at a number of CpGs in cohort 2. The extent of genotype-related effects, however, was not always matching the humanized mouse model, with strongest effects observed in the human data in intron 7 but not intron 5 (Supplementary Fig. 5B). In the humanized mouse, no significant genotype effects were detected in PFC and HIP (Supplementary Table 15 and 16). However, univariate effects of risk allele homozygosity on DNAm levels were observed in the human PFC primarily in intron 5 but also in intron 7 (Supplementary Fig. 6). Nonetheless, the three CpGs in intron 5 with the strongest reduction in DNAm with the TT genotype in human PFC were also those with the largest effect sizes in the humanized mouse model (Supplementary Fig. 6).

**Fig. 5:**
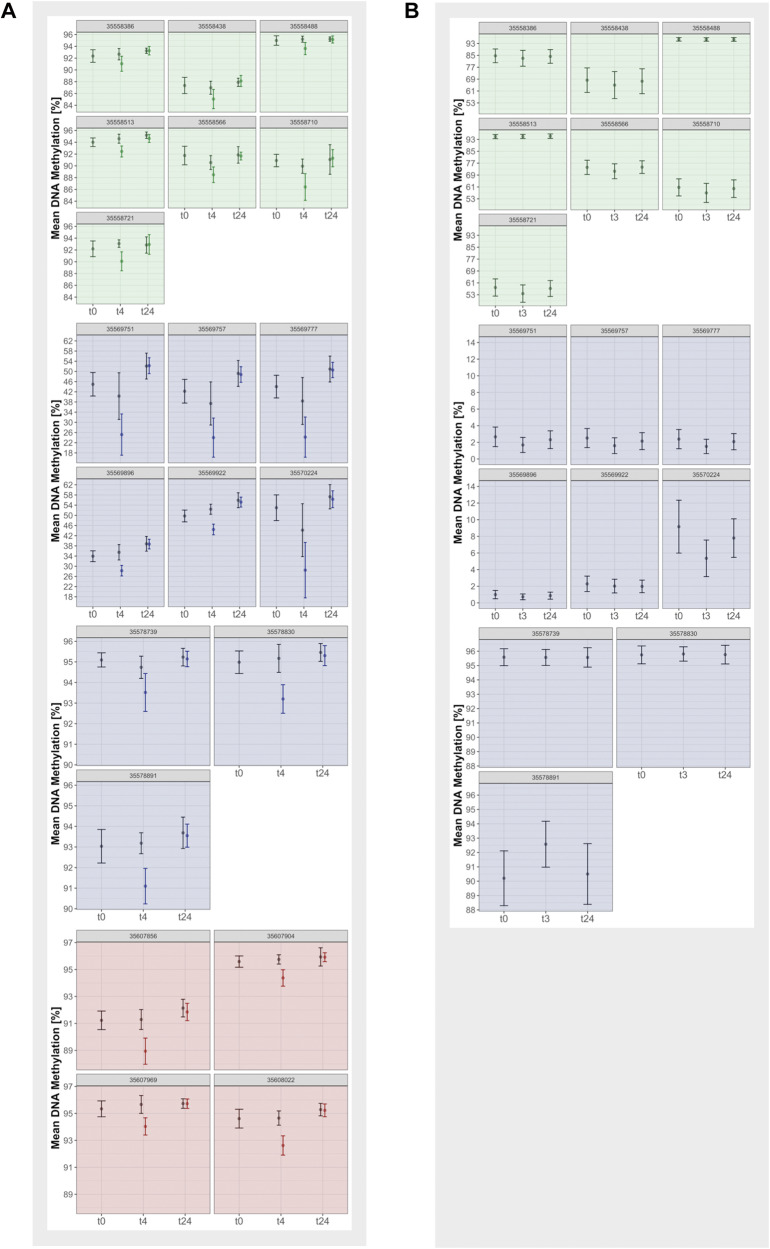
Dexamethasone effects on DNA methylation of each CpG in humanized *FKBP5* mouse and human blood. Depicted are mean and standard deviation of DNA methylation (in percent) of each CpG within introns of *FKBP5* (red: intron 2, blue: intron 5 and green: intron 7). **A** Sample group at baseline, 4 h, and 24 h after dexamethasone treatment in the humanized *FKBP5* mouse blood. **B** Samples at baseline, 3 h and 24 h after dexamethasone treatment in human blood (data from [36] was used).

As to dexamethasone effects, in the humanized mouse model, administration of dexamethasone was associated with significantly decreased DNAm after FDR correction in most blood CpGs, which returned to baseline after 24 h (Fig. 5A; Supplementary Fig. 5C; Supplementary Table 14). The strongest effects were seen in intron 5 (35569777, 35569757, 35569751). While the effects sizes very closely matched results from cohort 2 that had explored the effects of 1.5 mg dexamethasone orally in whole blood after 3 and 23 h in intron 7, the large effect sizes observed intron 5 of the humanized mice were not observed in cohort 2 (Supplementary Fig. 7). For the two brain regions, no human data was available for direct comparison of dexamethasone effects. The PFC showed no dexamethasone treatment effects (Supplementary Table 15). In the HIP, most intron 5 CpGs showed an increase in DNAm four h post-dexamethasone (35569922, 35569896, 35569751, 35569757 and 35569777; Supplementary Fig. 8), which was reversed after 24 h (Supplementary Table 16). Interaction effects of dexamethasone treatment with the risk allele were only nominal (*p* < 0.05) and did not survive correction for multiple testing. The strongest interaction was observed in intron 5 after 24 h in HIP in 35570224 (Supplementary Fig. 9; Supplementary Table 16).

## Discussion

In this study, we investigated DNAm patterns in functional intronic GREs of the *FKBP5* gene in brain and blood of a humanized *FKBP5* mouse model. This allowed cross tissue comparisons of human *FKBP5* GRE DNAm at baseline, in the context of a functional intronic variant and following GC-stimulation as well as comparisons with DNAm patterns of *FKBP5* in human tissues.

Overall, DNAm patterns of the humanized mouse model seemed to recapitulate DNAm patterns observed in native human tissue. This is likely attributable to the fact that the DNA sequence itself is one of the main drivers of local DNAm [63]. This was present to a higher extent in brain tissue, where DNAm patterns of relevant GREs within the *FKBP5* gene (introns 2, 5, and 7) of the humanized mouse model were highly similar to postmortem human tissue PFC. In blood, the least convergence was observed in intron 5, but with overall conserved pattern on DNAm. Differences in DNAm levels in blood could be related to differences in immune cell composition (*e.g*., neutrophils and lymphocytes balance) between mice and humans [64].

Beyond baseline levels, the humanized mouse model also mostly recapitulated effects of dexamethasone and, to a lesser extent, of genotype on DNAm. In fact, dexamethasone led to a reversible DNA demethylation in peripheral blood in all intronic GREs, very similar to previously reported effects in humans ([36], see Fig. 5). However, at CpGs with higher baseline methylation levels in humanized mice than humans such as in intron 5, effects sizes of dexamethasone-associated demethylation were larger in the animals, possibly suggesting effects of baseline DNAm on reactivity. While dexamethasone effects were observed in blood, most CpGs did not show altered DNAm in brain tissue. This could be due to lower GC-responsivity of specific regions such as the HIP, as previously suggested [65], but could also be attributed to a lower dose of dexamethasone and thus reduced intracerebral levels as compared to blood levels due to active extrusion at the blood brain barrier [66], differences in cell type heterogeneity or longer temporal dynamics of the GC-induced DNAm response in the brain. It is interesting to note that the CpG in location 35570224 in intron 5 showed the strongest DNAm change (25.4%) with dexamethasone in blood and a nominal interaction effect between dexamethasone treatment and genotype after 24 h in the HIP. Thus, following dexamethasone, only mice with the risk allele (A/T) showed a reduction in DNAm after 24 h. In a similar fine mapping of effects of dexamethasone on DNAm in the *FKBP5* locus of a human hippocampal progenitor cell line, the same CpG showed the strongest long-lasting effects of DNAm (−20.1% [67]).

While genotype effects were mostly recapitulated this was to a lesser extent than the dexamethasone effects and could be related to differences in genetic background and haplotype structure in humans as opposed to the animal model that only differed in this one SNP and limited power.

Our evaluation of DNAm patterns also revealed substantial tissue-specific DNAm across the three tissues/brain regions, an aspect previously reported for regions responsive to environmental stimuli [68–70]. While DNAm patterns in the humanized *FKBP5* mouse were similar between the two investigated brain regions (in mean DNAm and correlation structure), blood and brain concordance was low. Furthermore, there were also very little similarities of dexamethasone and genotype effects across brain and blood in the animal model. This is concordant with data from humans and confirms that differences are not due to differences in the timing of tissue extraction as in the case for postmortem brain studies, with brains sampled after death and blood often collected before. Our data therefore emphasizes the importance of tissue-specific DNAm levels, also with regard to genotype-associations and responsivity to environmental challenges.

Our study provides encouraging results regarding future use of humanized mouse models in the functional investigation of complex GxE that involve genetic and epigenetic regulation in non-coding elements. For example, Codagnone et al. have suggested that chronic selective inhibition of FKBP51 with the selective antagonist SAFit2 can induce stress resilience and change hippocampal neurogenesis in a chronic stress mouse model [39]. The humanized mouse model could elucidate whether such effects would be potentiated in a genotype-specific manner and/or are mediated by DNAm as a regulatory epigenetic mechanism, and could thus support genotype-guided treatment in patients.

Our results have several important limitations. First, the investigation was performed on a tissue-level, meaning an average across heterogeneous cells in each sample. Since DNAm is cell-type-specific, changes in DNAm of the whole tissue might not indicate effects on single cell types [71, 72]. Nold et al. showed that astrocytes derived from these humanized *FKBP5* mice have the strongest induction of *FKBP5*, yet the epigenetic correlates remain unknown [43]. Future studies thus need to evaluate cell-type-specific DNAm. Second, the analysis was limited to male mice. A recent study in the same model investigated sex-specific effects on the HPA axis and behavior after ELS modeled by prolonged maternal separation [44]. Females demonstrated higher corticosterone levels and more pronounced reduction after administration of dexamethasone. While slight genotype- and/or ELS-dependent behavioral differences were present in females, males were generally less affected behaviorally by genotype and ELS. Since sex-specific effects are plausible [73, 74], and evidence is expending for *FKBP5* [36, 37, 75], future studies should include the investigation of sex-specific effects. Third, results from humanized mouse models should be interpretated with caution due to differences in the immune system [64], the genetic background and the complex environment between the species. Finally, the interaction analysis of dexamethasone and genotype effects was likely underpowered due to the size of each individual group and results should be replicated in a larger cohort.

In conclusion, our analysis suggests that DNAm in GREs in the humanized *FKBP5* mouse model are similar to humans, especially in the PFC. Furthermore, we highlight the difficulties using peripheral blood as a proxy for changes in the brain. Given the necessity of exploring the molecular underpinnings of GxE interplay in psychiatric disorders, the recently engineered mice could present a powerful tool for studying the effects of human *FKBP5* polymorphism-related glucocorticoid response in disease-relevant tissues. Combined with naturalistic stress paradigms, behavioral tests, and/or neuroepigenetic editing, the humanized mouse can support mechanistic biological investigation of stress-related, *FKBP5*-induced psychopathology and enhance reverse translation of human findings.

### Supplementary information

Supplementary Information

Supplementary Table 7

## Data Availability

The raw data generated in the TBS experiments was uploaded to the Sequence Read Archive (SRA, https://www.ncbi.nlm.nih.gov/sra, BioProject accession numbers PRJNA1042684, PRJNA1042690, PRJNA1042916). Processed data and main analysis code in R are available in a public repository on Github Enterprise (https://github.molgen.mpg.de/mpip/Fkbp5_DNAm_HAMTBS_humanized_mouse_humans). Clinical data can be obtained upon a reasonable request.
