# Supplementary material for: DNA methylation patterns of FKBP5 regulatory regions in brain and blood of humanized mice and humans
Source: Mol Psychiatry. 2024 Feb 5;29(5):1510–20. doi: 10.1038/s41380-024-02430-x (PMC11189813; doi:10.1038/s41380-024-02430-x)
Supplement: Supplementary file 1 — Supplementary Information [file 41380_2024_2430_MOESM1_ESM.pdf]

Supplementary Information

for the article entitled "DNA methylation patterns of *FKBP5* regulatory regions in brain and blood of humanized mice and humans", Yusupov et al.

Summary

Table of Contents

1. Supplementary Methods
    1. Details of human cohorts
    2. Epigenome-wide postmortem brain DNA methylation
2. Supplementary Figures
3. Supplementary Tables
4. References

1. Supplementary Methods:

1.1. Details of human cohorts

For comparative analysis, we used DNA methylation (DNAm) data from three human cohorts. We quantified DNAm of two human cohorts: study 1 for peripheral blood (Final N=440) and study 3 for postmortem brain tissue (Final N=84).

Individuals included in study 1 are participants from a biobanking project at the Max Planck Institute of Psychiatry (MPIP) in Munich, Germany. They originated from two studies: Biological Classification of Mental Disorders study (BeCOME, see 1 for a full study protocol), observational study with psychiatric patients and self-reported healthy controls (Final N=314), and Optimized Treatment Identification at the Max Planck Institute (OPTIMA, see 2 for a full study protocol), a clinical psychotherapy study with patients recruited for major depressive

disorder (Final N=126). In short, diagnostic evaluation of participants from both studies was performed by a modified version of the computer-based Munich-Composite International Diagnostic Interview (DIA-X/M-CIDI, 3) conducted by trained study assistants. Current diagnosis (past for weeks) was defined by DSM-IV and transferred to ICD-10 system definitions (4, 5). Among participants with full current diagnosis (N=264, 60%), the following diagnostic categories were present (N, percentage from all participants with available diagnostic data): major depressive disorder (159, 37%), dysthymia (130, 30%), agoraphobia (79, 18%), social phobia (73, 17%), specific phobia (69, 16%), obsessive compulsive disorder (46, 11%), somatoform disorder (45, 10%), post-traumatic stress disorder (43, 10%), substance use/dependency (40, 9%), generalized anxiety disorder (27, 6%), bipolar disorder (12, 3%), panic disorder (11, 3%), eating disorder (4, 1%), organic mental disorder (6, 1%), unspecified screening of schizophrenia/psychotic spectrum disorders (2, 0,5%).

Individuals included in study 3 are brain donors from the NSW Brain Tissue Resource Centre (University of Sydney, Australia). This cohort consisted of patients with psychiatric disorders (N=51, 61%) with the following diagnosis (N, percentage): schizophrenia (36, 43%), major depressive disorder (6, 7%), schizoaffective disorder (5, 6%), bipolar disorder (N=4, 5%) as well as controls (33, 39%).

We additionally used previously acquired data from peripheral blood (study 2, 6). The study consisted of 59 (66.3%) patients with major depressive disorder and 30 healthy controls.

1.2. Epigenome-wide postmortem brain DNA methylation

400 ng DNA, originating from the same DNA extraction used for the high-accuracy DNAm measurement via targeted bisulfite sequencing (HAM-TBS), underwent bisulfite-conversion using the EZ-96 DNA Methylation kit (Zymo Research, Irvine, CA). Epigenome-wide DNAm quantification was with the Illumina Infinium MethylationEPIC BeadChip (Illumina, San Diego, CA, USA) according to manufacturer’s guidelines. Data was preprocessed as described in detail in Yusupov et al. (7). Briefly, raw intensity values were transformed into beta-values and

quality control was performed using with the *minfi* R package (8, 9). DNAm data was then normalized with stratified quantile normalization and subsequent beta-mixture quantile normalization (BMIQ) (10, 11). None of samples were excluded due to quality control issues (mean detection of p-value>0.05, distribution artefacts in raw beta-values or sex mismatches) or due to outlier samples of M-transformed values (> 3 standard deviation on two first principal components). Finally, we corrected sequentially for array and row as technical batch effects using ComBat of the *sva* R package (12). Batch-corrected M-values were transformed into beta-values and proportions of neuronal and non-neuronal cells calculated as suggested by Guintivano et al. (13). Final data was available for 75 participants. Further, brain tissue-related variables, which explaining variance in the principal components (brain pH and storage time) were included as a covariate in the analysis presented in supplementary figure 4.

2. Supplementary Figures:

Supplementary Figure 1. Exploration of interindividual variability of DNA methylation levels of CpGs in three tissues/brain regions of the humanized *FKBP5* mouse model. A: Histogram of interquartile ranges (IQR) of DNA methylation of the investigated CpGs. B-D: Different variability in tissues and functional elements of *FKBP5* (B: blood, C: prefrontal cortex, D: hippocampus). Generally, highest levels of interindividual variability were found in blood and prefrontal cortex and less in hippocampus. When accounting for functional regions (bars are colored for targeted introns - green: 7, blue: 5, red: 2), introns 7 and 5 presented the highest variability. Red line crosses the x-axis or y-axis at the chosen cut-off of 1%.

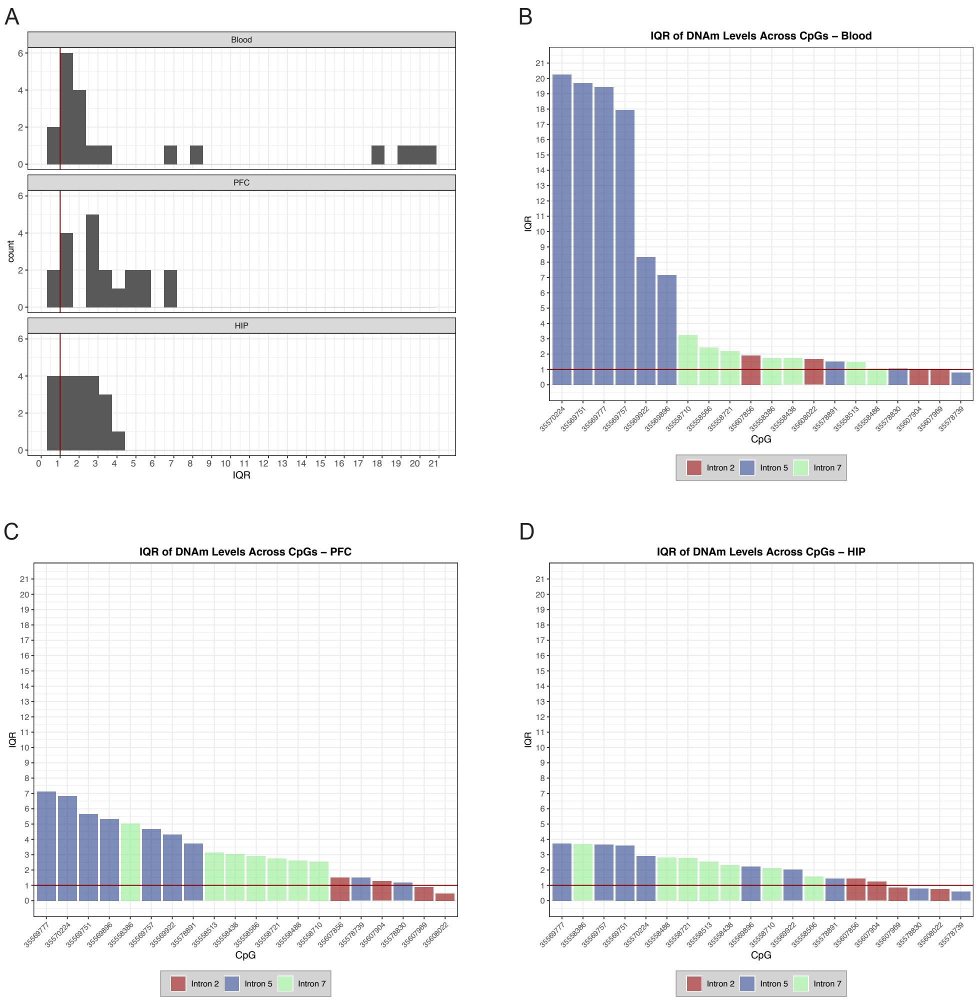

Supplementary Figure 2. Correlation matrix of DNA methylation in blood with prefrontal cortex and hippocampus of the humanized *FKBP5* mouse model. Spearman correlations are depicted. Both brain tissues are separated by a pink line. Not significant correlations are marked with n.

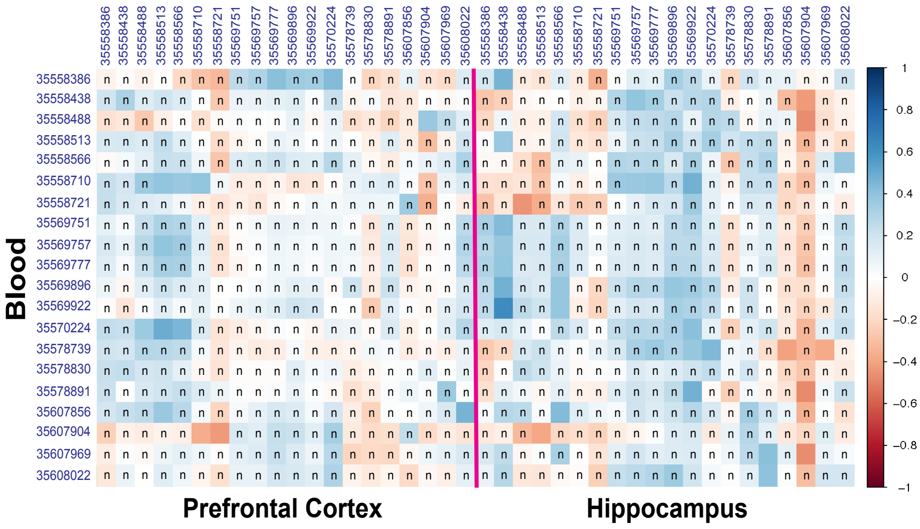

Supplementary Figure 3. Correlation matrix of CpGs for each of the humanized *FKBP5* mouse tissues/brain regions. Spearman correlations are depicted for CpGs with interquartile range > 1% located in different introns (separated by pink lines). Distinct correlation patterns are visible between the different tissues, which present higher similarity of correlation patterns among brain regions compared to blood.

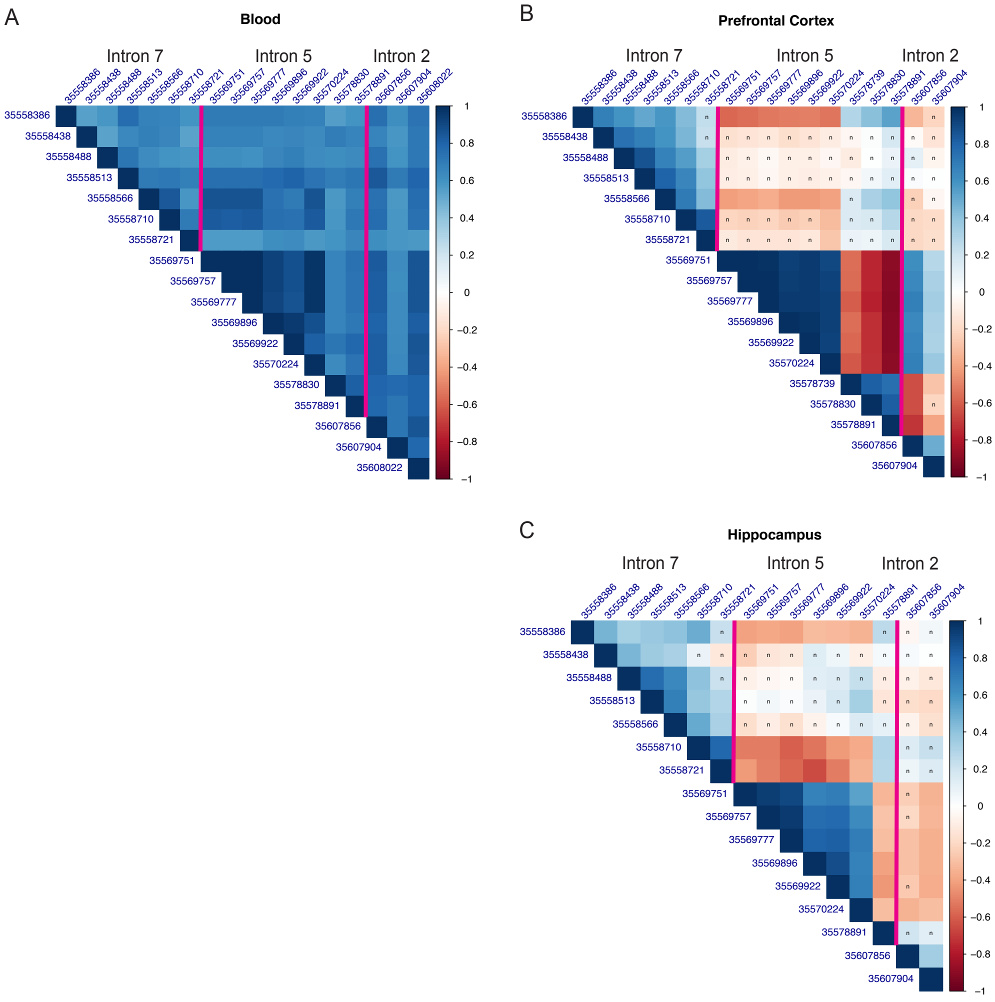

Supplementary Figure 4. DNA methylation differences related to psychiatric disease status. Depicted are boxplots of DNA methylation residuals from human blood (A: intron 7, C: intron 5, E: intron 2) and brain (B: intron 7, D: intron 5, F: intron 2) for subjects with- or without any current psychiatric diagnosis (p-values from a t-test are presented). Residuals were obtained by regressing DNA methylation on age, sex and calculated cell-type proportions (blood: CD8T, CD4T, NK, B lymphocytes, Monocytes and Granulocytes; brain: NeuN positive cells and NeuN negative cells). Final number of available subjects for the analysis was N=429 for blood (Yes: 264, No: 165) and N=72 for brain (Yes: 44, No: 28).

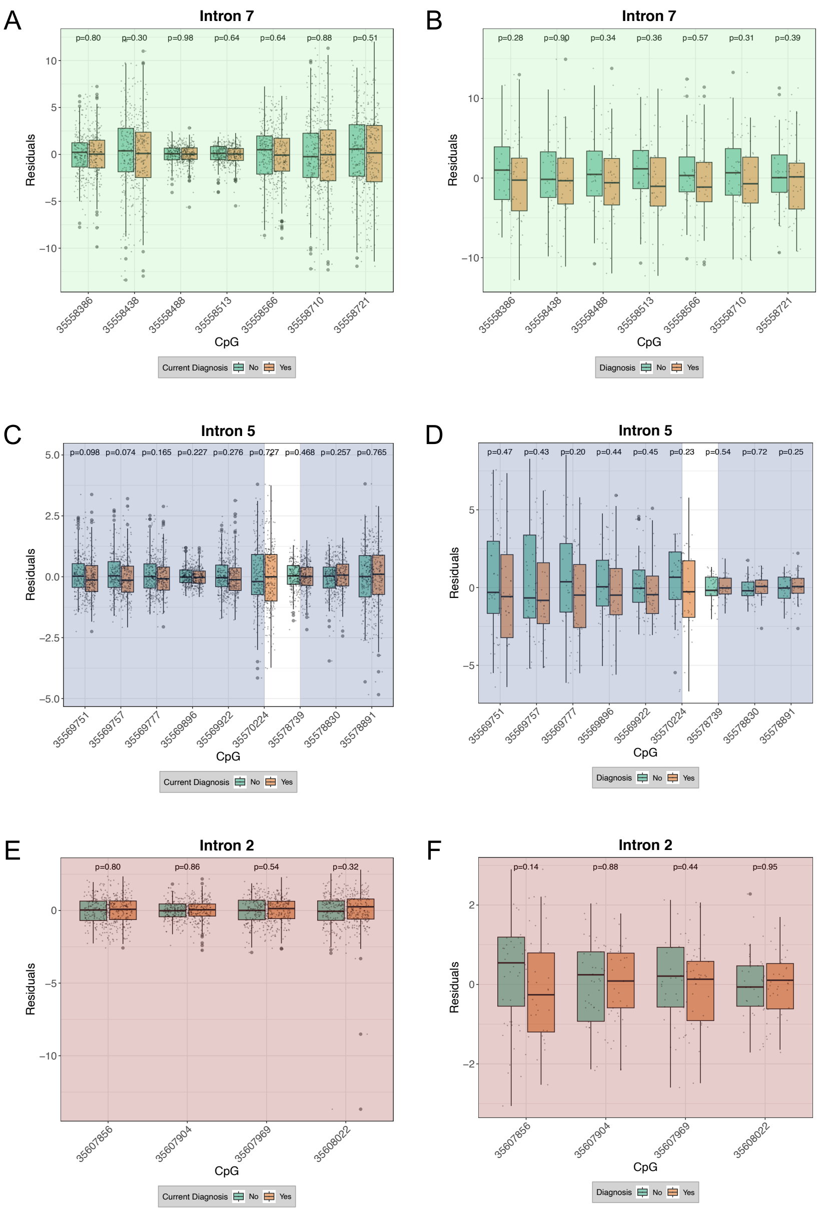

Supplementary Figure 5. Genotype effects on DNA methylation in humanized *FKBP5* mouse and human blood. A. Effects sizes (β) on DNA methylation (DNAm) levels (M-values) from multiple linear regression models are presented for the risk-allele of the rs1360780 SNP for each CpG. B. Differences in effects of risk allele homozygosity (TT) for the rs1360780 SNP in humanized *FKBP5* mouse and human blood (study 2) are presented as delta mean DNAm (in percent) for each CpG. C. Effect sizes (β) on DNAm levels (M-values) from multiple linear regression models are presented for dexamethasone (green) and its interaction with the risk-associated allele of rs1360780 SNP (yellow) for 18 CpGs in three introns of *FKBP5* (green: intron 7, blue: intron 5, red: intron 2). Darker bars: p-value<0.05, *: q-value<0.1, **: q-value<0.05.

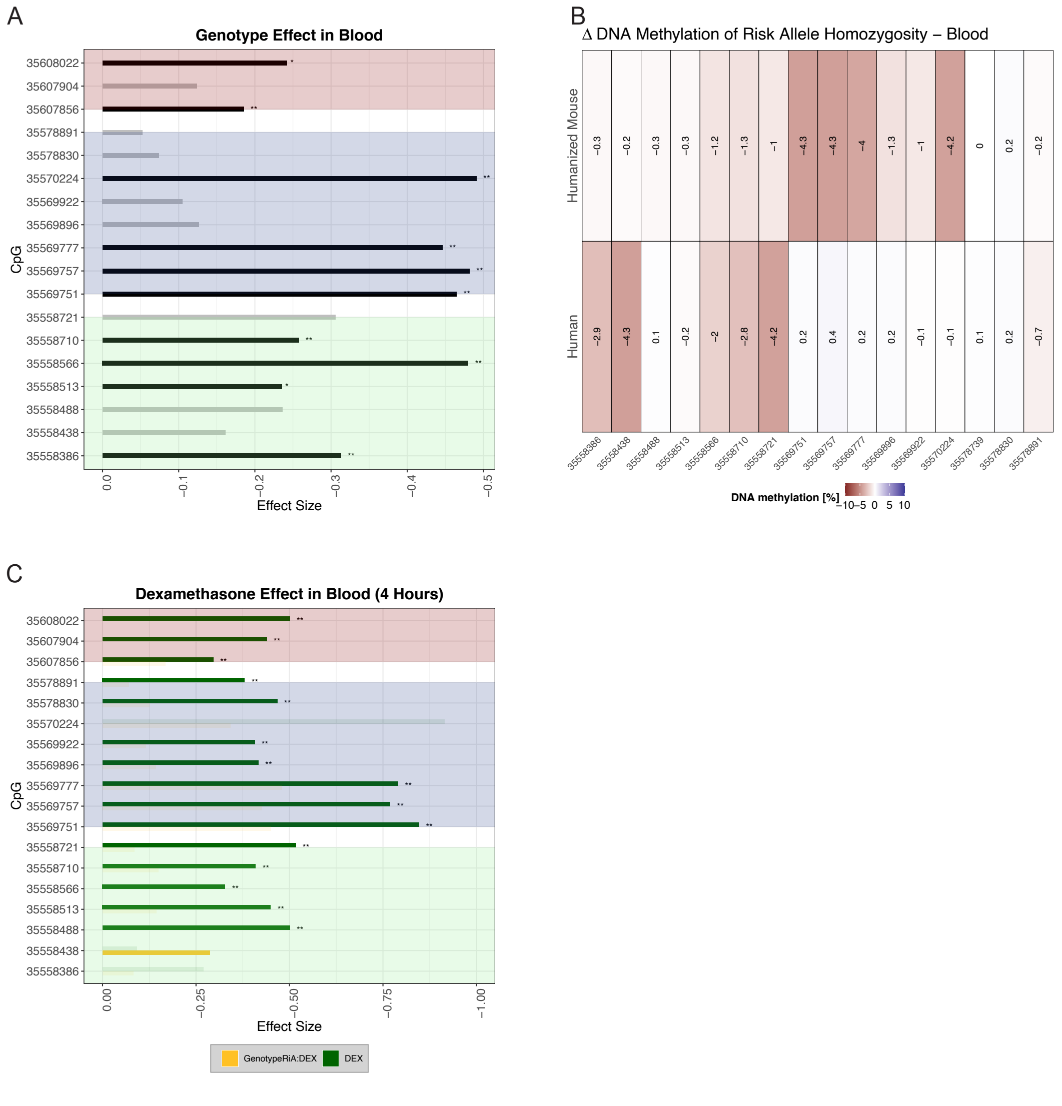

Supplementary Figure 6. Differences in genotype effects on DNA methylation in humanized *FKBP5* mouse and human prefrontal cortex. Differences in effects of risk allele homozygosity (TT) for the rs1360780 SNP in humanized *FKBP5* mouse and human postmortem PFC are presented as delta mean DNA methylation (in percent) for each CpG.

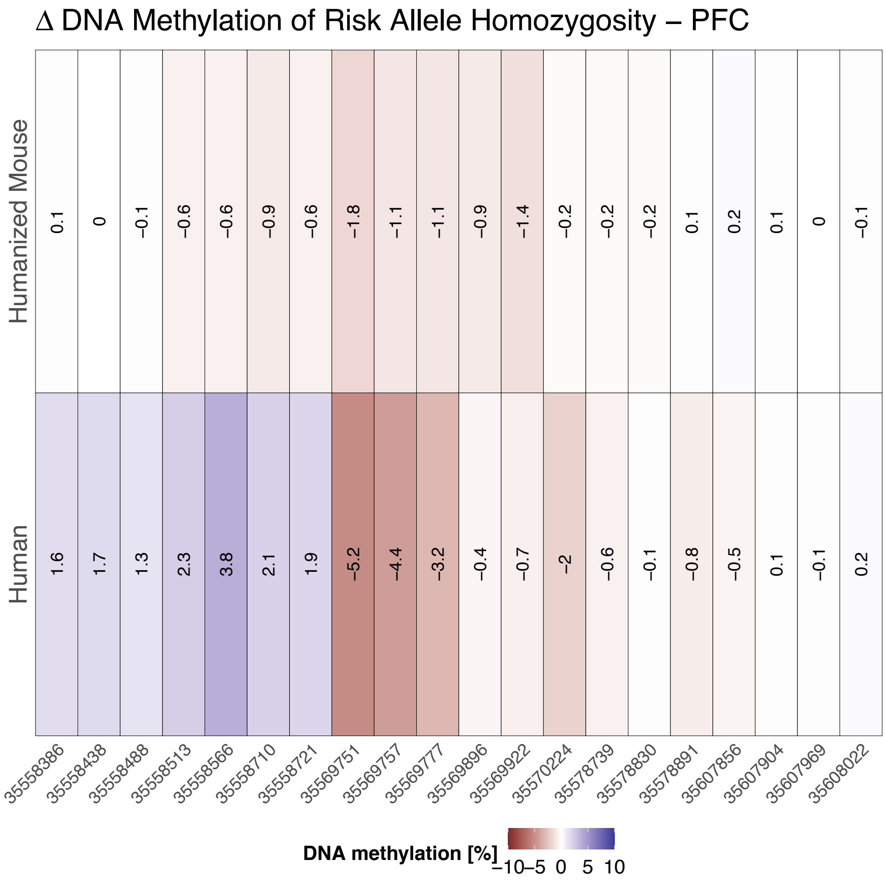

Supplementary Figure 7. Differences in dexamethasone effects on DNA methylation in humanized *FKBP5* mouse and human blood. Depicted are differences (delta mean DNA methylation) for humanized *FKBP5* mouse and human blood before and after the application of dexamethasone (humanized mouse: 4h, humans: 3h). Human data was available for introns 5 and 7.

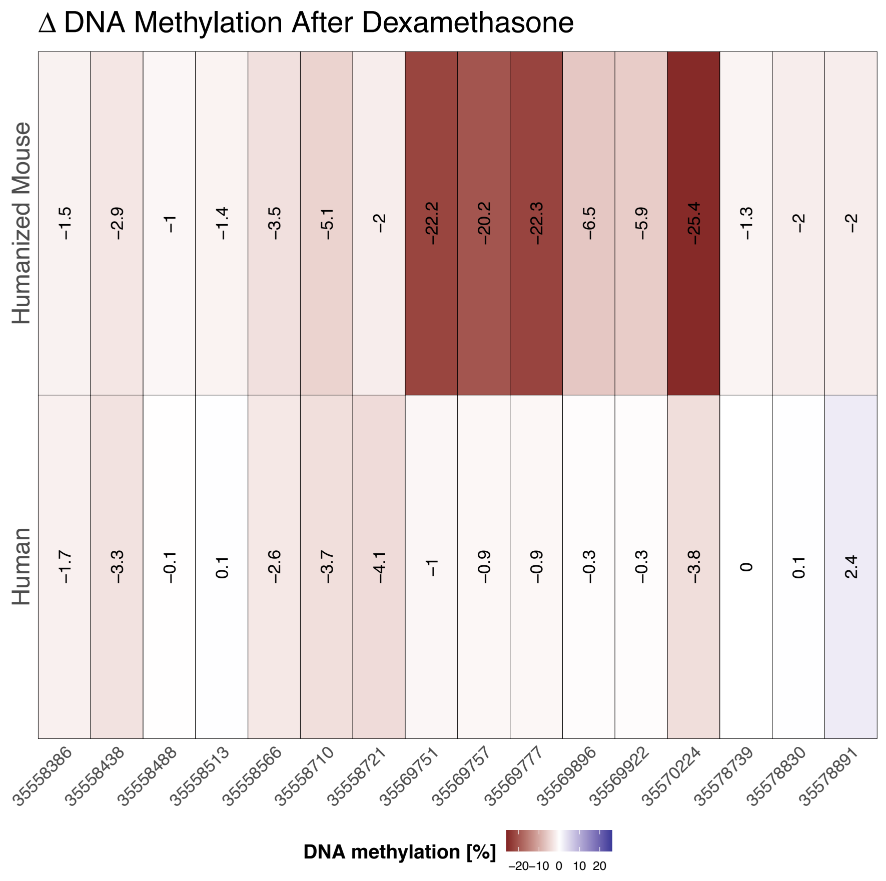

Supplementary Figure 8. Dexamethasone effects after four hours in humanized *FKBP5* mouse hippocampus. Effect sizes (β) on DNA methylation levels (M-values) from multiple linear regression models are presented for dexamethasone (green) and its interaction with the risk-associated allele of rs1360780 SNP (yellow) for 16 CpGs in three introns of *FKBP5* (green: intron 7, blue: intron 5, red: intron 2).

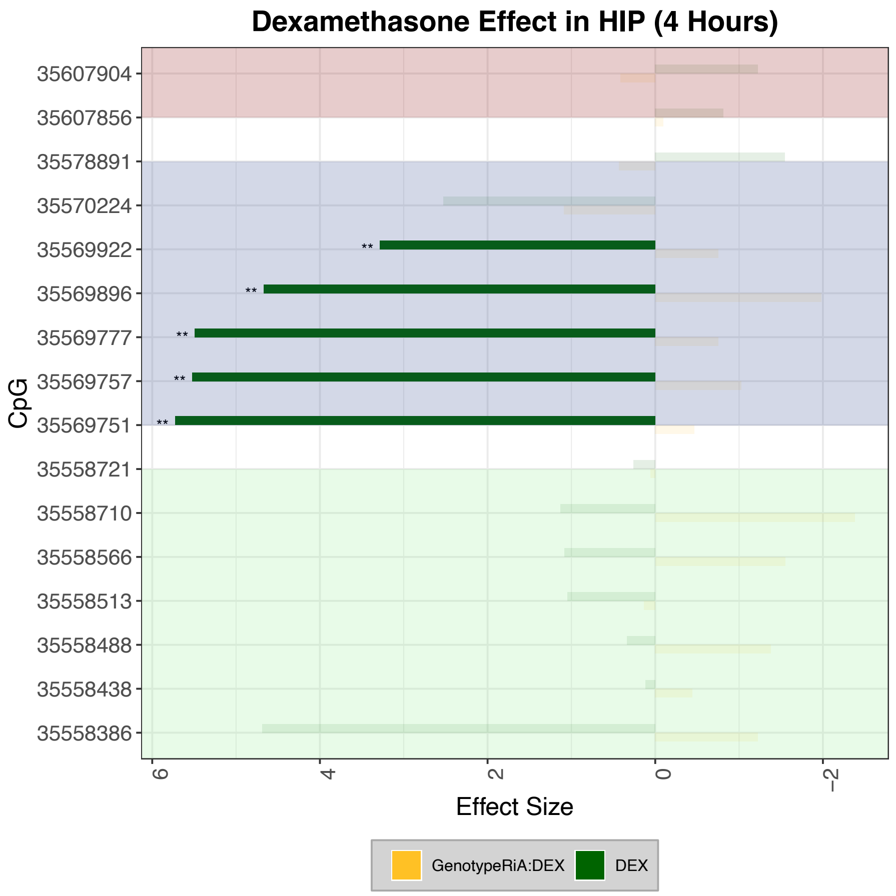

Supplementary Figure 9. Dexamethasone and interaction effects with rs1360780 genotype on DNA methylation in humanized *FKBP5* mouse hippocampus. A. Effect sizes (β) on DNA methylation (DNAm) levels (M-values) from multiple linear regression models are presented for dexamethasone (green) and its interaction with the risk-associated allele of rs1360780 SNP (yellow) for 16 CpGs in three introns of *FKBP5* (green: intron 7, blue: intron 5, red: intron 2) after 24 hours. Darker bars: p-value<0.05, *: q-value<0.1, **: q-value<0.05. B. DNAm levels (in percent) of CpG 35570224 (intron 5) are presented for each tissue/brain region (blood, prefrontal cortex and hippocampus) at baseline and after four and 24 hours of treatment with dexamethasone or vehicle. T-test was performed between the two rs1360780 genotypes, resilient (ReG) vs. risk (RiA) allele (ns: not significant, *: p<0.05, **: p<0.01). Nominally significant interaction effect was present in the hippocampus after 24 hours treatment with dexamethasone.

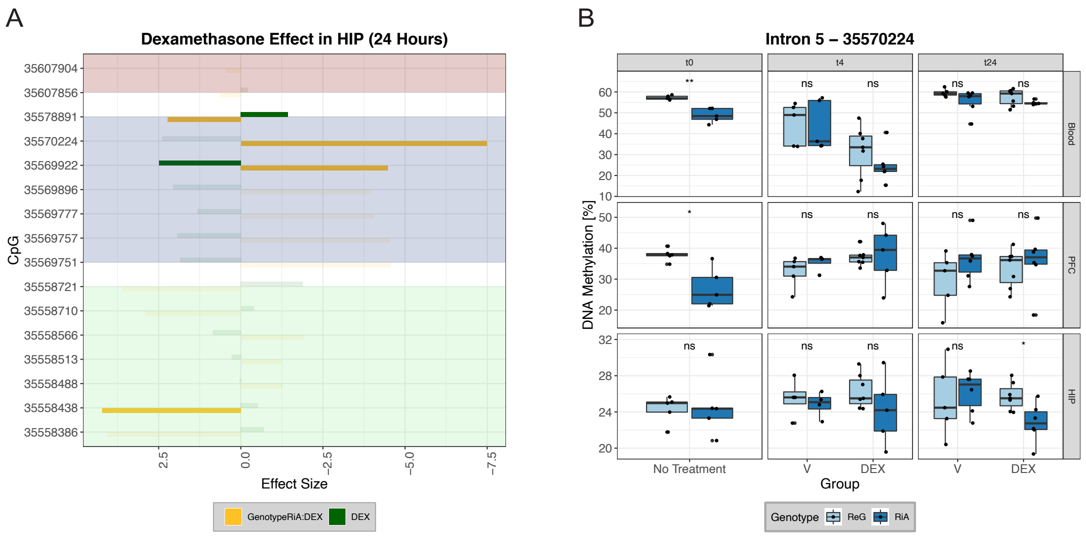

3. Supplementary Tables:

Supplementary Table 1. A table containing the number of mice for each genotype of the rs1360780 allele assigned to each of the treatment groups in each time point.

Genotype (rs1360780 allele)	Treatment condition	Time point	N
CG	dexamethasone	t4	7
CG	dexamethasone	t24	7
CG	vehicle	t4	6
CG	vehicle	t24	5
AT	dexamethasone	t4	6
AT	dexamethasone	t24	6
AT	vehicle	t4	5
AT	vehicle	t24	6
CG	not treated	t0	5
AT	not treated	t0	5

Supplementary Table 2. A table containing details of PCR primer sequences used in our panel along with the number of CpGs covered by each.

PCR Name	Functional region	Sequence	N covered CpGs
int7_1.2_F	intron 7	GAGAAGTATAAAAAAAAAATGG	5
int7_1.2_R	intron 7	AAAATCCAAAACTTATTCCC	5
int7_1.4_F	intron 7	ATAATTTGGAGTTATAGTGTAGG	5
int7_1.4_R	intron 7	CAACACTACTACTAAAAAAT	5
int5_P2_F	intron 5	ATTTAAAGGGGGAGGGA	5
int5_P2_R	intron 5	CCTTTTTTCCCCCCTAT	5
int5_P3_F	intron 5	AATTTTAAATTTAGGAAAAG	3
int5_P3_R	intron 5	AAACTAATCTCAACAAATCCAAC	3
FKBP5cg14284211_F	intron 5	AAATAATTATTGGGTTATGAGG	1
FKBP5cg14284211_R	intron 5	CCATCTCTACTTACTTTTTCAAA	1
int2_P4_F	intron 2	TGTATAGGTTTGTAGTTTTGGAGTAGT	4
int2_P4_R	intron 2	CCTTTCTCAAATTCAATTTATTCAC	4

Supplementary Table 3. A table containing details of PCR amplicons used in our panel.

Amplicon name	Chromosome	Start	Stop	Strand	Functional region	Length
FKBP5_int7_PCR_1.2	6	35558361	35558652	+	intron 7	291
FKBP5_int7_PCR_1.4	6	35558459	35558774	+	intron 7	315
FKBP5_int5_PCR_2	6	35569680	35569946	-	intron 5	266
FKBP5_int5_PCR_3	6	35578686	35578916	+	intron 5	230
FKBP5cg14284211	6	35570168	35570410	+	intron 5	242
FKBP5_int2_PCR_4	6	35607754	35608065	-	intron 2	311

Supplementary Table 4. A table containing genomic coordinates of the evaluated CpGs in three introns of the *FKBP5* gene, according to positions on the human reference genome hg19.

Chromosome	CpG location (hg19)	Functional region
6	35558386	intron 7
6	35558438	intron 7
6	35558488	intron 7
6	35558513	intron 7
6	35558566	intron 7
6	35558710	intron 7
6	35558721	intron 7
6	35569751	intron 5
6	35569757	intron 5
6	35569777	intron 5
6	35569896	intron 5
6	35569922	intron 5
6	35570224	intron 5
6	35578739	intron 5
6	35578830	intron 5
6	35578891	intron 5
6	35607856	intron 2
6	35607904	intron 2
6	35607969	intron 2
6	35608022	intron 2

Supplementary Table 5. A table containing mean and standard deviation of DNA methylation per CpG in each of the three tissues/brain regions (blood, prefrontal cortex and hippocampus) obtained from the humanized *FKBP5* mouse model.

Tissue	CpG	Mean	SD
Blood	35558386	92.77	0.91
Blood	35558438	87.42	1.12
Blood	35558488	95.14	0.58
Blood	35558513	94.62	0.83
Blood	35558566	91.40	1.47
Blood	35558710	90.68	1.78
Blood	35558721	92.70	1.21
Blood	35569751	45.99	8.06
Blood	35569757	43.16	7.84
Blood	35569777	44.66	8.23
Blood	35569896	36.17	3.35
Blood	35569922	52.80	3.52
Blood	35570224	51.68	8.86
Blood	35578739	95.03	0.48
Blood	35578830	95.22	0.58
Blood	35578891	93.32	0.74
Blood	35607856	91.57	0.79
Blood	35607904	95.76	0.51
Blood	35607969	95.58	0.55
Blood	35608022	94.86	0.63
HIP	35558386	68.38	2.85
HIP	35558438	45.02	2.04
HIP	35558488	47.59	1.64
HIP	35558513	38.09	1.63
HIP	35558566	23.60	1.28
HIP	35558710	22.55	2.03
HIP	35558721	25.07	1.95
HIP	35569751	48.64	3.11
HIP	35569757	43.54	2.90
HIP	35569777	40.76	2.92
HIP	35569896	19.55	1.90
HIP	35569922	18.08	1.54
HIP	35570224	25.18	2.56
HIP	35578739	95.44	0.49
HIP	35578830	95.33	0.55

HIP	35578891	90.53	0.85
HIP	35607856	75.27	0.91
HIP	35607904	92.89	0.81
HIP	35607969	93.56	0.66
HIP	35608022	91.68	0.55
PFC	35558386	57.52	4.05
PFC	35558438	44.04	2.29
PFC	35558488	46.11	2.28
PFC	35558513	35.79	2.18
PFC	35558566	18.45	1.66
PFC	35558710	15.00	1.77
PFC	35558721	17.70	1.71
PFC	35569751	58.29	6.85
PFC	35569757	52.88	6.85
PFC	35569777	50.96	6.74
PFC	35569896	26.98	4.86
PFC	35569922	24.03	3.94
PFC	35570224	33.16	6.86
PFC	35578739	92.37	0.87
PFC	35578830	93.85	0.77
PFC	35578891	79.03	3.29
PFC	35607856	79.67	1.51
PFC	35607904	93.86	0.81
PFC	35607969	94.13	0.61
PFC	35608022	93.28	0.64

Supplementary Table 6. A table containing delta mean percentage of DNA methylation between three tissues/brain regions (blood, prefrontal cortex and hippocampus) of the humanized *FKBP5* mouse model for each CpG in three introns of the *FKBP5* gene.

**CpG**	**Functional region**	**Blood**	**PFC**	**HIP**	**Delta DNAm Blood-PFC**	**Delta DNAm Blood-HIP**	**Delta DNAm PFC-HIP**
35558386	intron 7	92.77	57.52	68.38	35.26	24.39	-10.87
35558438	intron 7	87.42	44.04	45.02	43.38	42.40	-0.98
35558488	intron 7	95.14	46.11	47.59	49.04	47.55	-1.49
35558513	intron 7	94.62	35.79	38.09	58.83	56.53	-2.31
35558566	intron 7	91.40	18.45	23.60	72.95	67.80	-5.15

35558710	intron 7	90.68	15.00	22.55	75.68	68.13	-7.55
35558721	intron 7	92.70	17.70	25.07	75.00	67.63	-7.37
35569751	intron 5	45.99	58.29	48.64	-12.30	-2.64	9.66
35569757	intron 5	43.16	52.88	43.54	-9.72	-0.38	9.34
35569777	intron 5	44.66	50.96	40.76	-6.30	3.91	10.20
35569896	intron 5	36.17	26.98	19.55	9.18	16.62	7.44
35569922	intron 5	52.80	24.03	18.08	28.77	34.72	5.95
35570224	intron 5	51.68	33.16	25.18	18.52	26.50	7.98
35578739	intron 5	95.03	92.37	95.44	2.65	-0.42	-3.07
35578830	intron 5	95.22	93.85	95.33	1.37	-0.11	-1.48
35578891	intron 5	93.32	79.03	90.53	14.30	2.79	-11.51
35607856	intron 2	91.57	79.67	75.27	11.90	16.31	4.40
35607904	intron 2	95.76	93.86	92.89	1.90	2.88	0.98
35607969	intron 2	95.58	94.13	93.56	1.45	2.02	0.56
35608022	intron 2	94.86	93.28	91.68	1.59	3.18	1.60

Supplementary Table 7. A table containing a matrix of Spearman correlation coefficients between CpGs of three tissues/brain regions (blood, prefrontal cortex and hippocampus) obtained from the humanized *FKBP5* mouse model.

See table as a separate file for ease of readability.

Supplementary Table 8. A table containing t-test results for mean differences of DNA methylation of each CpG between the two human blood cohorts. Results are presented also for residualized values (after regressing out effects of age, sex and cell types).

CpG	Version	p-value	p-value adj.	p-value adj. significance
35558386	DNAm percent	1.2879E-06	2.0607E-05	****
35558438	DNAm percent	0.00094951	0.0151921	*
35558488	DNAm percent	0.00014629	0.00234064	**
35558513	DNAm percent	0.02345935	0.37534957	ns
35558566	DNAm percent	7.3624E-07	1.178E-05	****
35558710	DNAm percent	0.02513545	0.40216722	ns
35558721	DNAm percent	0.01968607	0.31497709	ns
35569751	DNAm percent	0.28055593	4.48889492	ns
35569757	DNAm percent	0.26962151	4.3139442	ns
35569777	DNAm percent	0.52081721	8.3330753	ns

35569896	DNAm percent	0.25403168	4.06450692	ns
35569922	DNAm percent	0.16468758	2.63500131	ns
35570224	DNAm percent	0.24007312	3.84116995	ns
35578739	DNAm percent	0.02170993	0.34735894	ns
35578830	DNAm percent	0.6937648	11.1002368	ns
35578891	DNAm percent	2.2892E-07	3.6627E-06	****
35558386	Residuals	1	1	ns
35558438	Residuals	1	1	ns
35558488	Residuals	1	1	ns
35558513	Residuals	1	1	ns
35558566	Residuals	1	1	ns
35558710	Residuals	1	1	ns
35558721	Residuals	1	1	ns
35569751	Residuals	1	1	ns
35569757	Residuals	1	1	ns
35569777	Residuals	1	1	ns
35569896	Residuals	1	1	ns
35569922	Residuals	1	1	ns
35570224	Residuals	1	1	ns
35578739	Residuals	1	1	ns
35578830	Residuals	1	1	ns
35578891	Residuals	1	1	ns

Supplementary Table 9. A table containing mean and standard deviation of DNA methylation per CpG in blood of humanized *FKBP5* mouse and human cohorts.

Study Cohort	CpG	Mean	SD
Human Study 1	35558386	87.34	3.77
Human Study 1	35558438	71.48	7.35
Human Study 1	35558488	95.25	1.00
Human Study 1	35558513	95.23	1.01
Human Study 1	35558566	77.00	3.72
Human Study 1	35558710	62.18	5.09
Human Study 1	35558721	59.44	5.26
Human Study 1	35569751	2.52	1.10
Human Study 1	35569757	2.37	1.09
Human Study 1	35569777	2.30	1.01
Human Study 1	35569896	0.93	0.42
Human Study 1	35569922	2.44	0.80
Human Study 1	35570224	8.74	2.32
Human Study 1	35578739	95.42	0.59

Human Study 1	35578830	95.77	0.69
Human Study 1	35578891	88.99	1.87
Human Study 1	35607856	91.99	0.96
Human Study 1	35607904	95.23	0.72
Human Study 1	35607969	94.02	0.98
Human Study 1	35608022	92.27	1.41
Human Study 2	35558386	84.67	4.62
Human Study 2	35558438	68.27	8.28
Human Study 2	35558488	95.78	1.19
Human Study 2	35558513	94.88	1.36
Human Study 2	35558566	74.17	4.79
Human Study 2	35558710	60.69	5.75
Human Study 2	35558721	57.87	5.80
Human Study 2	35569751	2.66	1.17
Human Study 2	35569757	2.52	1.14
Human Study 2	35569777	2.39	1.16
Human Study 2	35569896	1.00	0.50
Human Study 2	35569922	2.29	0.93
Human Study 2	35570224	9.16	3.18
Human Study 2	35578739	95.58	0.59
Human Study 2	35578830	95.74	0.62
Human Study 2	35578891	90.20	1.91
Humanized Mouse	35558386	92.77	0.91
Humanized Mouse	35558438	87.42	1.12
Humanized Mouse	35558488	95.14	0.58
Humanized Mouse	35558513	94.62	0.83
Humanized Mouse	35558566	91.40	1.47
Humanized Mouse	35558710	90.68	1.78
Humanized Mouse	35558721	92.70	1.21
Humanized Mouse	35569751	45.99	8.06
Humanized Mouse	35569757	43.16	7.84
Humanized Mouse	35569777	44.66	8.23
Humanized Mouse	35569896	36.17	3.35
Humanized Mouse	35569922	52.80	3.52
Humanized Mouse	35570224	51.68	8.86
Humanized Mouse	35578739	95.03	0.48
Humanized Mouse	35578830	95.22	0.58
Humanized Mouse	35578891	93.32	0.74
Humanized Mouse	35607856	91.57	0.79
Humanized Mouse	35607904	95.76	0.51
Humanized Mouse	35607969	95.58	0.55
Humanized Mouse	35608022	94.86	0.63

Supplementary Table 10. A table containing delta mean percentage of DNA methylation in blood between the humanized *FKBP5* mouse and the two human studies for each CpG.

CpG	Functional region	Humanized Mouse	Human Study 1	Human Study 2	Delta Mean DNAm Human Study 1	Delta Mean DNAm Human Study 2
35558386	intron 7	92.77	87.34	84.67	5.43	8.10
35558438	intron 7	87.42	71.48	68.27	15.95	19.15
35558488	intron 7	95.14	95.25	95.78	-0.11	-0.64
35558513	intron 7	94.62	95.23	94.88	-0.61	-0.26
35558566	intron 7	91.40	77.00	74.17	14.40	17.23
35558710	intron 7	90.68	62.18	60.69	28.50	29.99
35558721	intron 7	92.70	59.44	57.87	33.26	34.83
35569751	intron 5	45.99	2.52	2.66	43.48	43.33
35569757	intron 5	43.16	2.37	2.52	40.78	40.64
35569777	intron 5	44.66	2.30	2.39	42.36	42.28
35569896	intron 5	36.17	0.93	1.00	35.24	35.17
35569922	intron 5	52.80	2.44	2.29	50.36	50.51
35570224	intron 5	51.68	8.74	9.16	42.94	42.52
35578739	intron 5	95.03	95.42	95.58	-0.40	-0.56
35578830	intron 5	95.22	95.77	95.74	-0.55	-0.52
35578891	intron 5	93.32	88.99	90.20	4.33	3.12
35607856	intron 2	91.57	91.99	NA	-0.41	NA
35607904	intron 2	95.76	95.23	NA	0.53	NA
35607969	intron 2	95.58	94.02	NA	1.56	NA
35608022	intron 2	94.86	92.27	NA	2.59	NA

Supplementary Table 11. A table containing mean and standard deviation of DNA methylation per CpG in the prefrontal cortex of the human postmortem brain and the humanized *FKBP5* mouse model.

Species	CpG	Mean	SD
Human	35558386	59.76	6.05
Human	35558438	42.75	6.11
Human	35558488	37.91	5.96

Human	35558513	30.98	5.64
Human	35558566	22.65	5.82
Human	35558710	15.16	5.25
Human	35558721	15.25	5.10
Human	35569751	39.81	5.62
Human	35569757	36.91	5.67
Human	35569777	34.28	5.38
Human	35569896	16.78	3.76
Human	35569922	10.12	2.55
Human	35570224	25.74	4.64
Human	35578739	96.14	0.78
Human	35578830	96.07	0.79
Human	35578891	93.89	0.99
Human	35607856	88.93	2.12
Human	35607904	95.58	1.16
Human	35607969	95.87	1.16
Human	35608022	95.22	1.01
Humanized Mouse	35558386	57.52	4.05
Humanized Mouse	35558438	44.04	2.29
Humanized Mouse	35558488	46.11	2.28
Humanized Mouse	35558513	35.79	2.18
Humanized Mouse	35558566	18.45	1.66
Humanized Mouse	35558710	15.00	1.77
Humanized Mouse	35558721	17.70	1.71
Humanized Mouse	35569751	58.29	6.85
Humanized Mouse	35569757	52.88	6.85
Humanized Mouse	35569777	50.96	6.74
Humanized Mouse	35569896	26.98	4.86
Humanized Mouse	35569922	24.03	3.94
Humanized Mouse	35570224	33.16	6.86
Humanized Mouse	35578739	92.37	0.87
Humanized Mouse	35578830	93.85	0.77
Humanized Mouse	35578891	79.03	3.29
Humanized Mouse	35607856	79.67	1.51
Humanized Mouse	35607904	93.86	0.81
Humanized Mouse	35607969	94.13	0.61
Humanized Mouse	35608022	93.28	0.64

Supplementary Table 12. A table containing delta mean percentage of DNA methylation in prefrontal cortex between the humanized *FKBP5* mouse and the human postmortem tissue for each CpG in three introns of the *FKBP5* gene.

CpG	Functional region	Humanized Mouse	Human	Delta Mean DNAm
35558386	intron 7	57.52	59.76	-2.24
35558438	intron 7	44.04	42.75	1.29
35558488	intron 7	46.11	37.91	8.20
35558513	intron 7	35.79	30.98	4.80
35558566	intron 7	18.45	22.65	-4.21
35558710	intron 7	15.00	15.16	-0.17
35558721	intron 7	17.70	15.25	2.45
35569751	intron 5	58.29	39.81	18.48
35569757	intron 5	52.88	36.91	15.96
35569777	intron 5	50.96	34.28	16.68
35569896	intron 5	26.98	16.78	10.21
35569922	intron 5	24.03	10.12	13.91
35570224	intron 5	33.16	25.74	7.41
35578739	intron 5	92.37	96.14	-3.77
35578830	intron 5	93.85	96.07	-2.22
35578891	intron 5	79.03	93.89	-14.87
35607856	intron 2	79.67	88.93	-9.26
35607904	intron 2	93.86	95.58	-1.72
35607969	intron 2	94.13	95.87	-1.74
35608022	intron 2	93.28	95.22	-1.94

Supplementary Table 13. A table containing delta mean percentage of DNA methylation in prefrontal cortex and blood between the humanized *FKBP5* mouse and the human tissue of subjects aged between 20-29 years for each CpG in three introns of the *FKBP5* gene.

		PFC			Blood		
CpG	Functional region	Human 20-29	Humanized Mouse	Delta DNAm	Human 20-29	Humanized Mouse	Delta DNAm
35558386	intron 7	64.31	57.52	-6.80	88.16	92.77	4.61
35558438	intron 7	50.13	44.04	-6.09	73.37	87.42	14.06
35558488	intron 7	47.07	46.11	-0.97	95.24	95.14	-0.10
35558513	intron 7	36.82	35.79	-1.03	95.34	94.62	-0.72

35558566	intron 7	22.75	18.45	-4.30	77.22	91.40	14.18
35558710	intron 7	15.84	15.00	-0.85	62.40	90.68	28.28
35558721	intron 7	16.45	17.70	1.25	59.53	92.70	33.16
35569751	intron 5	52.54	58.29	5.76	2.50	45.99	43.50
35569757	intron 5	48.53	52.88	4.35	2.34	43.16	40.81
35569777	intron 5	44.53	50.96	6.43	2.28	44.66	42.39
35569896	intron 5	19.99	26.98	7.00	0.89	36.17	35.27
35569922	intron 5	13.79	24.03	10.25	2.48	52.80	50.32
35570224	intron 5	33.14	33.16	0.02	9.09	51.68	42.59
35578739	intron 5	96.80	92.37	-4.42	95.40	95.03	-0.37
35578830	intron 5	96.35	93.85	-2.50	95.77	95.22	-0.55
35578891	intron 5	94.19	79.03	-15.16	88.78	93.32	4.55
35607856	intron 2	89.94	79.67	-10.27	92.03	91.57	-0.46
35607904	intron 2	96.08	93.86	-2.22	95.29	95.76	0.47
35607969	intron 2	95.79	94.13	-1.66	94.11	95.58	1.47
35608022	intron 2	95.44	93.28	-2.16	92.72	94.86	2.14

Supplementary Table 14. Results of multiple linear regression models on DNA methylation for each investigated CpG in blood of the humanized *FKBP5* mouse at baseline, after four and 24 hours of dexamethasone treatment.

**Baseline**						
**CpG**	**term**	**estimate**	**std.error**	**statistic**	**p.value**	**q.value**
35558386	GenotypeRiA	-0.31299	0.097778	-3.20103	0.012593	0.020606
35558438	GenotypeRiA	-0.16106	0.099992	-1.61071	0.145909	0.181128
35558488	GenotypeRiA	-0.23615	0.13513	-1.74761	0.118664	0.152568
35558513	GenotypeRiA	-0.23524	0.095851	-2.45425	0.039673	0.057129
35558566	GenotypeRiA	-0.48008	0.113743	-4.22076	0.002913	0.005519
35558710	GenotypeRiA	-0.25749	0.086418	-2.97955	0.017612	0.027567
35558721	GenotypeRiA	-0.30569	0.151719	-2.01484	0.078686	0.104915
35569751	GenotypeRiA	-0.46482	0.077549	-5.99391	0.000326	0.000733
35569757	GenotypeRiA	-0.48166	0.076595	-6.28841	0.000236	0.000566
35569777	GenotypeRiA	-0.44611	0.078879	-5.65561	0.000478	0.001013
35569896	GenotypeRiA	-0.12673	0.080352	-1.57718	0.153406	0.184087
35569922	GenotypeRiA	-0.10508	0.081962	-1.28201	0.235742	0.26521
35570224	GenotypeRiA	-0.4909	0.091824	-5.34609	0.000689	0.001378
35578830	GenotypeRiA	-0.07387	0.108254	-0.68241	0.516917	0.54685
35578891	GenotypeRiA	-0.05196	0.129828	-0.40021	0.700937	0.700937
35607856	GenotypeRiA	-0.18533	0.05039	-3.67783	0.006239	0.010695

35607904	GenotypeRiA	-0.12383	0.093752	-1.32084	0.223085	0.259066
35608022	GenotypeRiA	-0.24211	0.094428	-2.56398	0.033439	0.050159
**4 Hours**						
**CpG**	**term**	**estimate**	**std.error**	**statistic**	**p.value**	**q.value**
35558386	GenotypeRiA	0.077935	0.143471	0.543209	0.593302	0.974974
35558386	GroupDEX	-0.269	0.132829	-2.02516	0.057133	0.060494
35558386	GenotypeRiA: GroupDEX	-0.0825	0.191082	-0.43174	0.670787	0.763178
35558438	GenotypeRiA	0.06477	0.092038	0.703736	0.490133	0.974974
35558438	GroupDEX	-0.09192	0.08521	-1.07878	0.29419	0.29419
35558438	GenotypeRiA: GroupDEX	-0.28751	0.12258	-2.34545	0.030012	0.54022
35558488	GenotypeRiA	0.042628	0.136554	0.312167	0.758313	0.974974
35558488	GroupDEX	-0.50075	0.126425	-3.96082	0.000838	0.001885
35558488	GenotypeRiA: GroupDEX	0.162338	0.181869	0.892609	0.383233	0.763178
35558513	GenotypeRiA	0.199453	0.127134	1.568839	0.13319	0.818579
35558513	GroupDEX	-0.44954	0.117703	-3.81927	0.001158	0.002084
35558513	GenotypeRiA: GroupDEX	-0.14379	0.169323	-0.84921	0.406336	0.763178
35558566	GenotypeRiA	-0.14746	0.121479	-1.21385	0.239679	0.862843
35558566	GroupDEX	-0.32781	0.112468	-2.91468	0.008887	0.014543
35558566	GenotypeRiA: GroupDEX	0.005563	0.161792	0.034386	0.972928	0.972928
35558710	GenotypeRiA	-0.09564	0.170697	-0.56029	0.582191	0.974974
35558710	GroupDEX	-0.40933	0.148997	-2.74722	0.013251	0.019876
35558710	GenotypeRiA: GroupDEX	-0.14909	0.221764	-0.67228	0.509944	0.763178
35558721	GenotypeRiA	0.003526	0.152632	0.023104	0.981821	0.981821
35558721	GroupDEX	-0.51675	0.133228	-3.87866	0.001101	0.002084
35558721	GenotypeRiA: GroupDEX	-0.08462	0.198294	-0.42673	0.674639	0.763178
35569751	GenotypeRiA	-0.12419	0.371592	-0.33421	0.741885	0.974974
35569751	GroupDEX	-0.84636	0.344028	-2.46016	0.02364	0.032732
35569751	GenotypeRiA: GroupDEX	-0.45028	0.494903	-0.90984	0.374303	0.763178
35569757	GenotypeRiA	-0.13722	0.355147	-0.38637	0.703519	0.974974
35569757	GroupDEX	-0.76912	0.328802	-2.33917	0.030404	0.038962
35569757	GenotypeRiA: GroupDEX	-0.42681	0.473	-0.90235	0.37817	0.763178
35569777	GenotypeRiA	-0.12535	0.370084	-0.33871	0.738545	0.974974
35569777	GroupDEX	-0.79053	0.342631	-2.30723	0.032468	0.038962

35569777	GenotypeRiA: GroupDEX	-0.47923	0.492894	-0.97227	0.343131	0.763178
35569896	GenotypeRiA	-0.01861	0.102312	-0.18192	0.857573	0.981821
35569896	GroupDEX	-0.4168	0.094723	-4.40017	0.000308	0.001384
35569896	GenotypeRiA: GroupDEX	-0.14309	0.136264	-1.05009	0.30685	0.763178
35569922	GenotypeRiA	0.035665	0.075631	0.471564	0.642608	0.974974
35569922	GroupDEX	-0.40674	0.07002	-5.80884	1.35E-05	0.000185
35569922	GenotypeRiA: GroupDEX	-0.11599	0.100728	-1.15147	0.263825	0.763178
35570224	GenotypeRiA	-0.07301	0.477072	-0.15304	0.879979	0.981821
35570224	GroupDEX	-0.91501	0.441683	-2.07165	0.052154	0.058674
35570224	GenotypeRiA: GroupDEX	-0.34138	0.635386	-0.53728	0.597312	0.763178
35578830	GenotypeRiA	0.181531	0.116448	1.558895	0.13643	0.818579
35578830	GroupDEX	-0.4671	0.111491	-4.18957	0.000551	0.001652
35578830	GenotypeRiA: GroupDEX	-0.12529	0.157672	-0.79459	0.437201	0.763178
35578891	GenotypeRiA	0.069638	0.092551	0.75243	0.46152	0.974974
35578891	GroupDEX	-0.37999	0.088611	-4.28827	0.000442	0.001593
35578891	GenotypeRiA: GroupDEX	-0.06987	0.125314	-0.55755	0.584021	0.763178
35607856	GenotypeRiA	0.211998	0.078853	2.688537	0.014544	0.261798
35607856	GroupDEX	-0.29615	0.073003	-4.05672	0.000673	0.001731
35607856	GenotypeRiA: GroupDEX	-0.16742	0.105019	-1.5942	0.127392	0.763178
35607904	GenotypeRiA	0.107517	0.085725	1.254213	0.224976	0.862843
35607904	GroupDEX	-0.43916	0.079366	-5.53333	2.45E-05	0.000185
35607904	GenotypeRiA: GroupDEX	0.048082	0.114172	0.421137	0.678381	0.763178
35608022	GenotypeRiA	0.003647	0.09968	0.03659	0.971194	0.981821
35608022	GroupDEX	-0.5009	0.092286	-5.42774	3.09E-05	0.000185
35608022	GenotypeRiA: GroupDEX	0.010574	0.132759	0.079649	0.937349	0.972928
**24 Hours**						
**CpG**	**term**	**estimate**	**std.error**	**statistic**	**p.value**	**q.value**
35558386	GenotypeRiA	0.011819	0.082102	0.143959	0.887049	0.887049
35558386	GroupDEX	0.061516	0.079392	0.774836	0.447973	0.914326
35558386	GenotypeRiA: GroupDEX	-0.12575	0.11421	-1.10106	0.284623	0.781271
35558438	GenotypeRiA	-0.01085	0.068875	-0.15747	0.876537	0.887049
35558438	GroupDEX	0.075294	0.066602	1.130506	0.272332	0.914326

35558438	GenotypeRiA: GroupDEX	-0.1049	0.09581	-1.09488	0.287255	0.781271
35558488	GenotypeRiA	-0.05626	0.092692	-0.60696	0.551059	0.708505
35558488	GroupDEX	0.049484	0.089632	0.552078	0.587331	0.914326
35558488	GenotypeRiA: GroupDEX	-0.16497	0.12894	-1.2794	0.216159	0.781271
35558513	GenotypeRiA	-0.2512	0.101118	-2.48427	0.022473	0.267959
35558513	GroupDEX	-0.23056	0.09778	-2.35798	0.029245	0.515021
35558513	GenotypeRiA: GroupDEX	0.087399	0.140662	0.62134	0.541759	0.781271
35558566	GenotypeRiA	-0.06068	0.133206	-0.45552	0.653897	0.784677
35558566	GroupDEX	-0.03959	0.128808	-0.30733	0.761939	0.914326
35558566	GenotypeRiA: GroupDEX	-0.05219	0.185298	-0.28163	0.781271	0.781271
35558710	GenotypeRiA	-0.31273	0.213781	-1.46285	0.159854	0.267959
35558710	GroupDEX	-0.08648	0.206724	-0.41836	0.680376	0.914326
35558710	GenotypeRiA: GroupDEX	0.127069	0.297384	0.427289	0.673972	0.781271
35558721	GenotypeRiA	-0.27462	0.189577	-1.4486	0.163753	0.267959
35558721	GroupDEX	0.039928	0.183319	0.217806	0.829903	0.93364
35558721	GenotypeRiA: GroupDEX	-0.10018	0.263715	-0.37987	0.708251	0.781271
35569751	GenotypeRiA	-0.23914	0.138937	-1.72122	0.101454	0.267959
35569751	GroupDEX	-0.04623	0.134351	-0.3441	0.734549	0.914326
35569751	GenotypeRiA: GroupDEX	0.062443	0.193271	0.323085	0.75016	0.781271
35569757	GenotypeRiA	-0.2321	0.141347	-1.64208	0.117022	0.267959
35569757	GroupDEX	-0.08311	0.136681	-0.60809	0.55033	0.914326
35569757	GenotypeRiA: GroupDEX	0.072619	0.196624	0.369328	0.715967	0.781271
35569777	GenotypeRiA	-0.2163	0.140607	-1.53831	0.140461	0.267959
35569777	GroupDEX	-0.07105	0.135965	-0.52257	0.607312	0.914326
35569777	GenotypeRiA: GroupDEX	0.056146	0.195594	0.287053	0.777179	0.781271
35569896	GenotypeRiA	-0.13528	0.088003	-1.53719	0.140734	0.267959
35569896	GroupDEX	-0.04412	0.085098	-0.51847	0.610119	0.914326
35569896	GenotypeRiA: GroupDEX	0.052781	0.122418	0.431154	0.671209	0.781271
35569922	GenotypeRiA	-0.13005	0.08481	-1.53345	0.141647	0.267959
35569922	GroupDEX	-0.08218	0.08201	-1.00211	0.328885	0.914326
35569922	GenotypeRiA: GroupDEX	0.05348	0.117976	0.453311	0.65546	0.781271
35570224	GenotypeRiA	-0.24167	0.136393	-1.77185	0.092457	0.267959
35570224	GroupDEX	-0.12492	0.13189	-0.94717	0.355442	0.914326

35570224	GenotypeRiA: GroupDEX	0.086381	0.189731	0.455281	0.654067	0.781271
35578830	GenotypeRiA	0.065951	0.094337	0.6991	0.492959	0.682559
35578830	GroupDEX	0.004265	0.091223	0.046756	0.963195	0.963195
35578830	GenotypeRiA: GroupDEX	-0.10968	0.131229	-0.83579	0.413663	0.781271
35578891	GenotypeRiA	-0.18655	0.089984	-2.07319	0.051997	0.267959
35578891	GroupDEX	-0.17614	0.087013	-2.02434	0.057225	0.515021
35578891	GenotypeRiA: GroupDEX	0.276222	0.125173	2.206713	0.039838	0.717082
35607856	GenotypeRiA	-0.01798	0.07192	-0.25004	0.805244	0.887049
35607856	GroupDEX	-0.00761	0.069546	-0.10944	0.914001	0.963195
35607856	GenotypeRiA: GroupDEX	-0.12291	0.100046	-1.22858	0.23423	0.781271
35607904	GenotypeRiA	0.170608	0.114953	1.484151	0.15417	0.267959
35607904	GroupDEX	0.054799	0.111159	0.49298	0.627676	0.914326
35607904	GenotypeRiA: GroupDEX	-0.12886	0.159908	-0.80584	0.430306	0.781271
35608022	GenotypeRiA	-0.11661	0.089369	-1.3048	0.207546	0.311318
35608022	GroupDEX	-0.05385	0.086419	-0.62312	0.540616	0.914326
35608022	GenotypeRiA: GroupDEX	0.05119	0.124319	0.411768	0.685118	0.781271

Supplementary Table 15. Results of multiple linear regression models on DNA methylation for each investigated CpG in the prefrontal cortex of the humanized *FKBP5* mouse at baseline, after four and 24 hours of dexamethasone treatment.

**Baseline**						
**CpG**	**term**	**estimate**	**std.error**	**statistic**	**p.value**	**q.value**
35558386	Dissecter Li	1.85	0.28	6.60714286	0.09562748	0.26196011
35558386	column2	-1.83	0.28	-6.5357143	0.09665669	0.26196011
35558386	column3	-15.29	0.28	-54.607143	0.01165687	0.17437452
35558386	column4	-17.34	0.3959798	-43.790113	0.01453545	0.17437452
35558386	column9	-9.85	0.3959798	-24.875006	0.02557897	0.17437452
35558386	column10	-5.91	0.28	-21.107143	0.03013881	0.17437452
35558386	column11	-9.76	0.37040518	-26.349523	0.02414899	0.17437452

35558386	column12	1.4	0.28	5	0.12566592	0.29938057
35558386	GenotypeRiA	NA	NA	NA	NA	NA
35558438	DissecterLi	2.4	2.56	0.9375	0.520529	0.59635025
35558438	column2	-2.02	2.56	-0.7890625	0.57471503	0.6376975
35558438	column3	-7.8	2.56	-3.046875	0.20189001	0.38434132
35558438	column4	-7.38	3.62038672	-2.0384563	0.2903451	0.43061941
35558438	column9	-6.29	3.62038672	-1.7373835	0.33248657	0.44885687
35558438	column10	-5.77	2.56	-2.2539063	0.26584028	0.42552271
35558438	column11	-5.28	3.38656168	-1.5591035	0.36306533	0.46591765
35558438	column12	-4.71	2.56	-1.8398438	0.31694622	0.44648077
35558438	GenotypeRiA	NA	NA	NA	NA	NA
35558488	DissecterLi	5.635	2.89	1.94982699	0.30168607	0.43250569
35558488	column2	-0.965	2.89	-0.33391	0.79483687	0.81287676
35558488	column3	-8.705	2.89	-3.0121107	0.20406456	0.38434132
35558488	column4	-10.08	4.0870772	-2.4663102	0.24523092	0.41382719
35558488	column9	-10.77	4.0870772	-2.635135	0.23090125	0.41105498
35558488	column10	-5.755	2.89	-1.9913495	0.29627248	0.43061941
35558488	column11	-9.13	3.82311064	-2.3881077	0.25245653	0.41889992
35558488	column12	-2.175	2.89	-0.7525952	0.58927843	0.64069198
35558488	GenotypeRiA	NA	NA	NA	NA	NA
35558513	DissecterLi	3.735	2.605	1.43378119	0.38771218	0.48771272
35558513	column2	-0.205	2.605	-0.0786948	0.95000436	0.95590501
35558513	column3	-6.195	2.605	-2.378119	0.25340859	0.41889992
35558513	column4	-7.61	3.68402633	-2.0656747	0.28701976	0.43061941
35558513	column9	-7.295	3.68402633	-1.9801704	0.29771219	0.43061941
35558513	column10	-5.775	2.605	-2.2168906	0.2697699	0.42552271

35558513	column11	-6.0875	3.44609108	-1.7664942	0.32793218	0.44885687
35558513	column12	-2.72	2.605	-1.0441459	0.48625354	0.57921378
35558513	GenotypeRiA	NA	NA	NA	NA	NA
35558566	DissecterLi	3.73	1.165	3.20171674	0.19272531	0.37616265
35558566	column2	-1.065	1.165	-0.9141631	0.52852886	0.59875297
35558566	column3	-6.98	1.165	-5.9914163	0.10528481	0.26196011
35558566	column4	-7.08	1.6475588	-4.297267	0.1455548	0.32991717
35558566	column9	-8.325	1.6475588	-5.0529304	0.12438296	0.29938057
35558566	column10	-4.265	1.165	-3.6609442	0.1697539	0.35714456
35558566	column11	-7.2025	1.54115014	-4.6734577	0.13419665	0.31507039
35558566	column12	0.005	1.165	0.00429185	0.99726774	0.99726774
35558566	GenotypeRiA	NA	NA	NA	NA	NA
35558710	DissecterLi	-0.71	2.16	-0.3287037	0.79782349	0.81287676
35558710	column2	-4.48	2.16	-2.0740741	0.28600787	0.43061941
35558710	column3	-7.8	2.16	-3.6111111	0.17198487	0.35719934
35558710	column4	-6.07	3.05470129	-1.987101	0.2968181	0.43061941
35558710	column9	-6.42	3.05470129	-2.1016785	0.28272867	0.43061941
35558710	column10	-8	2.16	-3.7037037	0.16788417	0.35714456
35558710	column11	-7.15	2.85741142	-2.5022648	0.24203917	0.41382719
35558710	column12	-6.19	2.16	-2.8657407	0.2137381	0.39347242
35558710	GenotypeRiA	NA	NA	NA	NA	NA
35558721	DissecterLi	-1.7	0.48	-3.5416667	0.17519092	0.35925226
35558721	column2	-4.25	0.48	-8.8541667	0.07159719	0.2319749
35558721	column3	-5.68	0.48	-11.833333	0.05367133	0.19659142
35558721	column4	-4.12	0.67882251	-6.0693332	0.10395724	0.26196011
35558721	column9	-5.3	0.67882251	-7.8076374	0.08109656	0.24132326

35558721	column10	-5.79	0.48	-12.0625	0.05265636	0.19659142
35558721	column11	-7.14	0.63498031	-11.244443	0.05646783	0.19659142
35558721	column12	-5.45	0.48	-11.354167	0.05592496	0.19659142
35558721	GenotypeRiA	NA	NA	NA	NA	NA
35569751	DissecterLi	13.78	3.67	3.75476839	0.16570334	0.35714456
35569751	column2	-9.11	3.67	-2.4822888	0.24380265	0.41382719
35569751	column3	10.92	3.67	2.97547684	0.20640553	0.38434132
35569751	column4	10.52	5.19016377	2.02691099	0.29177734	0.43061941
35569751	column9	9.08	5.19016377	1.7494631	0.33058284	0.44885687
35569751	column10	6.55	3.67	1.78474114	0.32513492	0.44885687
35569751	column11	9.865	4.85495366	2.03194525	0.29115121	0.43061941
35569751	column12	-12.34	3.67	-3.3623978	0.18403151	0.36357445
35569751	GenotypeRiA	NA	NA	NA	NA	NA
35569757	DissecterLi	15.07	3.87	3.89405685	0.16002701	0.35032941
35569757	column2	-8.74	3.87	-2.2583979	0.26537076	0.42552271
35569757	column3	9.85	3.87	2.54521964	0.23832818	0.41382719
35569757	column4	8.56	5.47300649	1.56403981	0.36215139	0.46591765
35569757	column9	7.83	5.47300649	1.43065791	0.38836383	0.48771272
35569757	column10	6.43	3.87	1.66149871	0.34491462	0.45692186
35569757	column11	9.005	5.11952879	1.75895095	0.3291014	0.44885687
35569757	column12	-11.73	3.87	-3.0310078	0.20287695	0.38434132
35569757	GenotypeRiA	NA	NA	NA	NA	NA
35569777	DissecterLi	16.76	3.93	4.26463104	0.14662985	0.32991717
35569777	column2	-6.46	3.93	-1.6437659	0.34794025	0.45692186
35569777	column3	10.39	3.93	2.6437659	0.23021155	0.41105498
35569777	column4	8.6	5.5578593	1.54735835	0.36525643	0.46591765

35569777	column9	7.46	5.5578593	1.34224341	0.40763219	0.50409477
35569777	column10	2.7	3.93	0.6870229	0.61677826	0.65768471
35569777	column11	8.625	5.19890133	1.65900437	0.34533735	0.45692186
35569777	column12	-9.04	3.93	-2.3002545	0.26106942	0.42552271
35569777	GenotypeRiA	NA	NA	NA	NA	NA
35569896	DissecterLi	12.29	0.31	39.6451613	0.01605454	0.17437452
35569896	column2	-3.77	0.31	-12.16129	0.05223054	0.19659142
35569896	column3	6.72	0.31	21.6774194	0.02934707	0.17437452
35569896	column4	5.01	0.4384062	11.427758	0.05556665	0.19659142
35569896	column9	5.17	0.4384062	11.7927163	0.05385531	0.19659142
35569896	column10	-0.24	0.31	-0.7741935	0.58059105	0.63983504
35569896	column11	6.165	0.41009145	15.0332321	0.0422852	0.19659142
35569896	column12	-5.63	0.31	-18.16129	0.0350183	0.18879567
35569896	GenotypeRiA	NA	NA	NA	NA	NA
35569922	DissecterLi	9.92	0.6	16.5333333	0.03845838	0.18879567
35569922	column2	-3.68	0.6	-6.1333333	0.10289135	0.26196011
35569922	column3	4.67	0.6	7.78333333	0.08134704	0.24132326
35569922	column4	5.32	0.84852814	6.26968013	0.1006913	0.26196011
35569922	column9	5.19	0.84852814	6.11647366	0.10317003	0.26196011
35569922	column10	0.98	0.6	1.63333333	0.34974266	0.45692186
35569922	column11	4.75	0.79372539	5.98443749	0.10540536	0.26196011
35569922	column12	-5.02	0.6	-8.3666667	0.07573076	0.23711207
35569922	GenotypeRiA	NA	NA	NA	NA	NA
35570224	DissecterLi	17.32	0.35	49.4857143	0.01286297	0.17437452
35570224	column2	-4.61	0.35	-13.171429	0.04824084	0.19659142
35570224	column3	7.54	0.35	21.5428571	0.02953012	0.17437452

35570224	column4	6.41	0.494974<br>75	12.9501556	0.04906188	0.19659142
35570224	column9	5.51	0.494974<br>75	11.131881	0.05703578	0.19659142
35570224	column1<br>0	-2.47	0.35	-7.0571429	0.08961268	0.25468867
35570224	column1<br>1	7.835	0.463006<br>48	16.9220094	0.03757711	0.18879567
35570224	column1<br>2	-7.72	0.35	-22.057143	0.02884254	0.17437452
35570224	Genotyp<br>eRiA	NA	NA	NA	NA	NA
35578739	Dissecter<br>Li	-1.58	0.22	-7.1818182	0.08807696	0.25468867
35578739	column2	-0.74	0.22	-3.3636364	0.18396746	0.36357445
35578739	column3	-1.7	0.22	-7.7272727	0.08193074	0.24132326
35578739	column4	-2.59	0.311126<br>98	-8.3245753	0.07611005	0.23711207
35578739	column9	-0.43	0.311126<br>98	-1.3820723	0.39875179	0.49690608
35578739	column1<br>0	0.62	0.22	2.81818182	0.21707394	0.39512336
35578739	column1<br>1	-1.18	0.291032<br>64	-4.054528	0.15394216	0.34162506
35578739	column1<br>2	-1.3	0.22	-5.9090909	0.10672449	0.26196011
35578739	Genotyp<br>eRiA	NA	NA	NA	NA	NA
35578830	Dissecter<br>Li	-0.69	0.62	-1.1129032	0.46601447	0.56567531
35578830	column2	0.76	0.62	1.22580645	0.43563559	0.53464368
35578830	column3	-1.37	0.62	-2.2096774	0.27054839	0.42552271
35578830	column4	-1.64	0.876812<br>41	-1.8704115	0.31256465	0.44417082
35578830	column9	-0.97	0.876812<br>41	-1.10628	0.46790427	0.56567531
35578830	column1<br>0	0.47	0.62	0.75806452	0.58706139	0.64069198
35578830	column1<br>1	-0.68	0.820182<br>91	-0.8290834	0.55931598	0.62489095
35578830	column1<br>2	0.44	0.62	0.70967742	0.60708376	0.65565046
35578830	Genotyp<br>eRiA	NA	NA	NA	NA	NA
35578891	Dissecter<br>Li	-7.28	0.17	-42.823529	0.01486342	0.17437452

35578891	column2	2.82	0.17	16.5882353	0.0383314	0.18879567
35578891	column3	-5.53	0.17	-32.529412	0.01956443	0.17437452
35578891	column4	-7.52	0.24041631	-31.279076	0.02034597	0.17437452
35578891	column9	-2.4	0.24041631	-9.982684	0.06356037	0.21013836
35578891	column10	-0.43	0.17	-2.5294118	0.23968119	0.41382719
35578891	column11	-5.405	0.22488886	-24.034094	0.02647293	0.17437452
35578891	column12	1.7	0.17	10	0.06345103	0.21013836
35578891	GenotypeRiA	NA	NA	NA	NA	NA
35607856	DissecterLi	3.01	0.87	3.45977011	0.17912474	0.36272759
35607856	column2	0.95	0.87	1.09195402	0.47203471	0.56644166
35607856	column3	0.81	0.87	0.93103448	0.52272676	0.59635025
35607856	column4	1.25	1.2303658	1.01595802	0.49496072	0.58104084
35607856	column9	-0.7	1.2303658	-0.5689365	0.67069859	0.70554007
35607856	column10	-3.99	0.87	-4.5862069	0.13667265	0.31629957
35607856	column11	0.465	1.15090182	0.40403099	0.75555295	0.78461268
35607856	column12	0.22	0.87	0.25287356	0.84232115	0.85285016
35607856	GenotypeRiA	NA	NA	NA	NA	NA
35607904	DissecterLi	0.47	1.36	0.34558824	0.7881717	0.81287676
35607904	column2	0.67	1.36	0.49264706	0.70858859	0.74058937
35607904	column3	1.39	1.36	1.02205882	0.49305533	0.58104084
35607904	column4	1.32	1.92333044	0.68630952	0.61708689	0.65768471
35607904	column9	1.28	1.92333044	0.66551226	0.62617497	0.6630088
35607904	column10	-1.33	1.36	-0.9779412	0.50709955	0.59100811
35607904	column11	1.55	1.79911089	0.86153667	0.54726551	0.61567369
35607904	column12	1.31	1.36	0.96323529	0.51192033	0.59236495
35607904	GenotypeRiA	NA	NA	NA	NA	NA

4 Hours						
**CpG**	**term**	**estimate**	**std.error**	**statistic**	**p.value**	**q.value**
35558386	DissecterLi	-1.2939569	1.79467555	-0.7209977	0.48742424	0.77939049
35558386	column2	-7.7933627	2.82768107	-2.7560968	0.02026509	0.30053396
35558386	column4	-6.5396623	4.43633523	-1.4741137	0.17122143	0.99071103
35558386	column9	-3.9493126	3.52847864	-1.1192678	0.28918958	0.43378438
35558386	column10	-2.2197926	2.31437046	-0.9591345	0.36010639	0.49860885
35558386	column11	-1.6805251	3.47163528	-0.484073	0.63876013	0.76651215
35558386	column12	0.05782465	2.65011535	0.02181967	0.98302105	0.98302105
35558386	GenotypeRiA	-1.4322315	2.59362477	-0.5522123	0.59292942	0.94177674
35558386	GroupDEX	-1.8908808	2.85491208	-0.6623254	0.52273326	0.7694266
35558386	GenotypeRiA:GroupDEX	1.264499	3.3773885	0.3744014	0.71592268	0.97189416
35558438	DissecterLi	0.89315932	1.49262723	0.59838069	0.56289313	0.77939049
35558438	column2	-3.4964459	2.35177538	-1.4867261	0.16792107	0.32281838
35558438	column4	-0.0440421	3.68968909	-0.0119365	0.99071103	0.99071103
35558438	column9	-0.2489699	2.934627	-0.0848387	0.9340641	0.9340641
35558438	column10	-1.3227796	1.92485621	-0.6872095	0.50757446	0.57102126

35558438	column11	-2.427988	2.88735051	-0.8409052	0.42005372	0.54006907
35558438	column12	-1.7939158	2.20409441	-0.8139015	0.43465366	0.66171803
35558438	GenotypeRiA	0.6610511	2.15711134	0.30645201	0.76554605	0.94177674
35558438	GroupDEX	2.25488277	2.37442335	0.9496549	0.36467375	0.7694266
35558438	GenotypeRiA:GroupDEX	-1.8797595	2.8089657	-0.6691999	0.51851876	0.97189416
35558488	DissecterLi	0.54596693	1.5523516	0.35170314	0.73235909	0.86195608
35558488	column2	-3.2957565	2.44587677	-1.3474745	0.20756298	0.32281838
35558488	column4	-0.1596894	3.83732431	-0.0416148	0.96762496	0.99071103
35558488	column9	-3.5473647	3.05204998	-1.1622892	0.27210601	0.43378438
35558488	column10	-1.414008	2.00187532	-0.7063417	0.4961029	0.57102126
35558488	column11	-4.2279459	3.00288183	-1.4079628	0.1894617	0.28419255
35558488	column12	-1.8706212	2.29228665	-0.8160503	0.4334796	0.66171803
35558488	GenotypeRiA	0.21747996	2.24342365	0.0969411	0.92468855	0.94177674
35558488	GroupDEX	0.42372244	2.46943095	0.17158708	0.86718471	0.86718471
35558488	GenotypeRiA:GroupDEX	-0.5889178	2.92136061	-0.2015903	0.84427871	0.97189416
35558513	DissecterLi	-0.2975666	1.56817769	-0.1897531	0.85329891	0.90349297
35558513	column2	-2.8467821	2.47081227	-1.1521644	0.2760532	0.3822275

35558513	column4	-0.4715862	3.8764455	-0.1216543	0.90558274	0.99071103
35558513	column9	-2.5557956	3.08316537	-0.8289518	0.42647505	0.59050392
35558513	column10	-1.574001	2.02228427	-0.7783283	0.45439754	0.57102126
35558513	column11	-3.2621182	3.03349595	-1.0753659	0.30747156	0.42572985
35558513	column12	-0.7758277	2.31565632	-0.3350358	0.74451999	0.78831529
35558513	GenotypeRiA	0.42849749	2.26629516	0.189074	0.85381712	0.94177674
35558513	GroupDEX	2.48215281	2.49460659	0.99500772	0.34319894	0.7694266
35558513	GenotypeRiA:GroupDEX	-2.4373647	2.95114363	-0.8259052	0.42812226	0.97189416
35558566	DissecterLi	-0.487508	1.2381149	-0.3937502	0.70203049	0.86195608
35558566	column2	-0.4463046	1.95076713	-0.2287842	0.82364758	0.82364758
35558566	column4	-0.9238186	3.06054917	-0.3018473	0.76895251	0.99071103
35558566	column9	0.83494188	2.43423497	0.34299971	0.73869987	0.79441752
35558566	column10	0.54574048	1.59664322	0.3418049	0.73957195	0.73957195
35558566	column11	-0.3776232	2.39501974	-0.1576702	0.87785494	0.92949346
35558566	column12	-1.0228627	1.82826768	-0.559471	0.58815108	0.66171803
35558566	GenotypeRiA	-0.3148988	1.78929583	-0.1759903	0.86381438	0.94177674
35558566	GroupDEX	1.68482665	1.96955332	0.85543592	0.41233666	0.7694266

35558566	GenotypeRiA:GroupDEX	-2.7124649	2.33000056	-1.1641478	0.27138631	0.97189416
35558710	DissecterLi	-0.6624817	1.01859147	-0.65039	0.53169026	0.77939049
35558710	column2	0.68940244	1.56183151	0.44140641	0.6693331	0.7351455
35558710	column4	-1.5201463	2.4478319	-0.6210175	0.54999048	0.99071103
35558710	column9	0.63889024	1.94677852	0.32817819	0.75028322	0.79441752
35558710	column10	0.85592073	1.34423874	0.63673268	0.54015408	0.57192785
35558710	column11	-0.7232439	1.91498359	-0.3776763	0.71442389	0.80372687
35558710	column12	0.82379268	1.46724934	0.56145377	0.58819381	0.66171803
35558710	GenotypeRiA	-0.2662866	1.43267117	-0.1858672	0.85667055	0.94177674
35558710	GroupDEX	0.54312805	1.5845952	0.34275508	0.73965212	0.83210864
35558710	GenotypeRiA:GroupDEX	-2.009878	1.8978085	-1.0590521	0.31717053	0.97189416
35558721	DissecterLi	-1.6321098	0.8163838	-1.9991942	0.07665234	0.17246776
35558721	column2	0.50808537	1.25178149	0.40588982	0.69430408	0.7351455
35558721	column4	-2.028122	1.96189578	-1.0337562	0.32822768	0.99071103
35558721	column9	0.96965854	1.56030999	0.6214525	0.54971679	0.70677873
35558721	column10	2.14297561	1.07738457	1.98905356	0.0779153	0.13822048
35558721	column11	-0.0825366	1.53482689	-0.0537758	0.95828854	0.95828854
35558721	column12	1.2542439	1.17597548	1.06655617	0.31394605	0.66171803

35558721	GenotypeRiA	-0.1232805	1.14826166	-0.1073627	0.91685648	0.94177674
35558721	GroupDEX	-0.8857683	1.2700262	-0.697441	0.50314403	0.7694266
35558721	GenotypeRiA:GroupDEX	-2.2307317	1.52106135	-1.4665626	0.17654539	0.97189416
35569751	DissecterLi	6.09629259	2.38424401	2.556908	0.02852403	0.12970715
35569751	column2	-4.9708818	3.75660195	-1.3232389	0.21521225	0.32281838
35569751	column4	-0.5848697	5.89371473	-0.0992362	0.9229119	0.99071103
35569751	column9	-8.3393788	4.68761837	-1.7790225	0.10559455	0.23338305
35569751	column10	-5.8887776	3.07466378	-1.915259	0.08446807	0.13822048
35569751	column11	-8.9157515	4.61210141	-1.9331213	0.0820097	0.19512068
35569751	column12	-2.6802605	3.52070415	-0.7612854	0.4640609	0.66171803
35569751	GenotypeRiA	0.25805611	3.44565587	0.07489318	0.94177674	0.94177674
35569751	GroupDEX	-2.8714228	3.79277861	-0.7570763	0.46646768	0.7694266
35569751	GenotypeRiA:GroupDEX	1.58496994	4.48689365	0.35324437	0.73123842	0.97189416
35569757	DissecterLi	5.64982365	2.5010005	2.2590254	0.04744697	0.13097462
35569757	column2	-5.6112014	3.94056283	-1.4239594	0.18490498	0.32281838
35569757	column4	-0.46151	6.18233008	-0.0746499	0.94196552	0.99071103
35569757	column9	-8.8962786	4.91717115	-1.8092269	0.10052896	0.23338305

35569757	column10	-6.7762094	3.22523014	-2.1010003	0.06197296	0.13822048
35569757	column11	-9.8577114	4.83795613	-2.0375777	0.068926	0.19512068
35569757	column12	-3.06548	3.69311311	-0.8300531	0.4258807	0.66171803
35569757	GenotypeRiA	1.59222645	3.61438972	0.44052429	0.66892751	0.94177674
35569757	GroupDEX	-2.1638136	3.97851107	-0.5438752	0.59844291	0.7694266
35569757	GenotypeRiA:GroupDEX	-0.4992285	4.70661693	-0.1060695	0.91762483	0.97189416
35569777	DissecterLi	6.55396393	2.95594783	2.21721231	0.05093458	0.13097462
35569777	column2	-6.3698707	4.65737538	-1.3676954	0.20135627	0.32281838
35569777	column4	-0.6054339	7.30693385	-0.0828574	0.9356	0.99071103
35569777	column9	-10.453762	5.81163475	-1.7987644	0.10225784	0.23338305
35569777	column10	-8.2024178	3.81191928	-2.1517816	0.0568932	0.13822048
35569777	column11	-10.860305	5.71801003	-1.8993154	0.0867203	0.19512068
35569777	column12	-2.6556323	4.36491305	-0.6084044	0.55648535	0.66171803
35569777	GenotypeRiA	1.95370541	4.27186938	0.45734203	0.65720009	0.94177674
35569777	GroupDEX	-2.6980301	4.70222664	-0.5737771	0.57879409	0.7694266
35569777	GenotypeRiA:GroupDEX	-0.2410922	5.56277943	-0.0433402	0.96628351	0.97189416
35569896	DissecterLi	6.09888978	1.98490763	3.07263153	0.01178781	0.12970715

35569896	column2	-5.7431884	3.12740971	-1.8364042	0.09616299	0.3208469
35569896	column4	0.27736874	4.90657806	0.05652998	0.95603318	0.99071103
35569896	column9	-8.0005491	3.90249045	-2.0501137	0.06749545	0.23338305
35569896	column10	-5.7736934	2.55968918	-2.2556228	0.04772191	0.13822048
35569896	column11	-9.0158196	3.8396218	-2.348101	0.04077247	0.19512068
35569896	column12	-3.3077375	2.93102237	-1.1285269	0.28544384	0.66171803
35569896	GenotypeRiA	0.91526653	2.86854391	0.31907008	0.75623843	0.94177674
35569896	GroupDEX	-2.6552585	3.15752716	-0.8409297	0.42004059	0.7694266
35569896	GenotypeRiA:GroupDEX	0.29360721	3.73538505	0.07860159	0.93890012	0.97189416
35569922	DissecterLi	4.59780862	1.81028474	2.5398262	0.0293719	0.12970715
35569922	column2	-6.3522725	2.85227483	-2.2270899	0.05008899	0.30053396
35569922	column4	-2.0017325	4.47492027	-0.4473225	0.66417548	0.99071103
35569922	column9	-7.9302625	3.55916759	-2.2281228	0.05000136	0.23338305
35569922	column10	-5.8897585	2.3344997	-2.5229211	0.03023558	0.13822048
35569922	column11	-7.416505	3.50182984	-2.1178942	0.06023673	0.19512068
35569922	column12	-4.1910351	2.67316473	-1.5678177	0.14799422	0.66171803
35569922	GenotypeRiA	-0.7626463	2.61618283	-0.2915111	0.77661783	0.94177674

35569922	GroupDEX	-3.0095762	2.87974269	-1.0450851	0.32058817	0.7694266
35569922	GenotypeRiA:GroupDEX	1.7648998	3.40676335	0.51805765	0.61568534	0.97189416
35570224	DissecterLi	7.14838377	2.95324709	2.42051666	0.03602976	0.12970715
35570224	column2	-8.1074168	4.65312011	-1.7423614	0.1120563	0.3208469
35570224	column4	-0.3796202	7.30025777	-0.0520009	0.95955205	0.99071103
35570224	column9	-11.402343	5.80632487	-1.9637797	0.07794561	0.23338305
35570224	column10	-9.151013	3.80843647	-2.4028267	0.03713584	0.13822048
35570224	column11	-12.764537	5.71278569	-2.2343805	0.04947359	0.19512068
35570224	column12	-3.4212595	4.36092498	-0.7845261	0.45091605	0.66171803
35570224	GenotypeRiA	2.45821743	4.26796633	0.57596927	0.57736748	0.94177674
35570224	GroupDEX	-1.9002635	4.69793039	-0.4044895	0.69436881	0.83210864
35570224	GenotypeRiA:GroupDEX	1.20425852	5.55769692	0.21668301	0.83281197	0.97189416
35578739	DissecterLi	-1.1018868	0.42434884	-2.5966532	0.02664407	0.12970715
35578739	column2	1.58730261	0.66860174	2.37406293	0.03900578	0.30053396
35578739	column4	0.99837575	1.04896605	0.95177127	0.36365045	0.99071103
35578739	column9	1.20194589	0.83430447	1.44065618	0.18024899	0.32444819
35578739	column10	1.08710321	0.54723007	1.98655605	0.07504959	0.13822048
35578739	column11	1.30317836	0.82086393	1.58756928	0.14346747	0.26440392

35578739	column12	0.4027485	0.62661654	0.64273518	0.53485418	0.66171803
35578739	GenotypeRiA	-0.261492	0.61325941	-0.426397	0.67885165	0.94177674
35578739	GroupDEX	0.14201102	0.67504048	0.21037408	0.83760036	0.86718471
35578739	GenotypeRiA:GroupDEX	0.34856713	0.79857939	0.43648401	0.67175902	0.97189416
35578830	DissecterLi	-0.5664319	0.37601747	-1.5063977	0.16288362	0.29319052
35578830	column2	0.30008918	0.59245109	0.50652144	0.62347078	0.7351455
35578830	column4	-0.3617916	0.92949366	-0.3892351	0.70526222	0.99071103
35578830	column9	0.30899399	0.73928104	0.41796553	0.6848056	0.79441752
35578830	column10	0.96195591	0.48490309	1.98381064	0.07539322	0.13822048
35578830	column11	1.1107976	0.72737131	1.52713968	0.15771508	0.26440392
35578830	column12	0.85458317	0.55524781	1.53910228	0.15479986	0.66171803
35578830	GenotypeRiA	-0.8716102	0.543412	-1.6039584	0.13980482	0.94177674
35578830	GroupDEX	-0.5287766	0.59815648	-0.8840104	0.3974463	0.7694266
35578830	GenotypeRiA:GroupDEX	1.0659519	0.70762488	1.50637991	0.16288813	0.97189416
35578891	DissecterLi	-3.0415541	1.66365486	-1.8282362	0.09745643	0.19491286
35578891	column2	5.07644389	2.62124558	1.93665329	0.08153158	0.3208469
35578891	column4	-0.1094008	4.1124596	-0.0266023	0.97930035	0.99071103
35578891	column9	5.61685772	3.27088127	1.71723069	0.11669152	0.23338305
35578891	column10	4.94262325	2.14540933	2.30381363	0.04396801	0.13822048

35578891	column11	7.88954309	3.21818778	2.4515484	0.03416713	0.19512068
35578891	column12	2.0328016	2.45664309	0.82747128	0.42727499	0.66171803
35578891	GenotypeRiA	-1.2794419	2.40427662	-0.5321525	0.60624063	0.94177674
35578891	GroupDEX	1.83295491	2.64648859	0.69259883	0.50432685	0.7694266
35578891	GenotypeRiA:GroupDEX	1.59086172	3.13082151	0.50812917	0.62238282	0.97189416
35607856	DissecterLi	-0.2641232	0.86430525	-0.3055902	0.76618318	0.86195608
35607856	column2	-2.2816834	1.36179467	-1.6754973	0.1247738	0.3208469
35607856	column4	0.39047595	2.13651312	0.18276319	0.85863596	0.99071103
35607856	column9	-4.1292685	1.69929469	-2.4299897	0.03545082	0.23338305
35607856	column10	-2.4338026	1.11458728	-2.1835909	0.05391718	0.13822048
35607856	column11	-4.0537074	1.67191926	-2.4245832	0.03578011	0.19512068
35607856	column12	-1.7234519	1.27628006	-1.3503713	0.20666409	0.66171803
35607856	GenotypeRiA	-0.1757565	1.24907453	-0.1407094	0.89089354	0.94177674
35607856	GroupDEX	-1.5038527	1.37490897	-1.0937835	0.29969667	0.7694266
35607856	GenotypeRiA:GroupDEX	0.0808517	1.62653056	0.04970808	0.96133386	0.97189416
35607904	DissecterLi	0.02313126	0.39147364	0.05908766	0.9540464	0.9540464
35607904	column2	-0.503512	0.61680374	-0.8163245	0.43332993	0.55713848

35607904	column4	-1.1136573	0.96770043	-1.1508286	0.27657734	0.99071103
35607904	column9	-1.7466733	0.76966913	-2.2693821	0.04661951	0.23338305
35607904	column10	-0.6439379	0.504835	-1.2755413	0.23095119	0.34642678
35607904	column11	-1.1446693	0.75726986	-1.5115739	0.16158018	0.26440392
35607904	column12	-0.4326854	0.57807123	-0.7484984	0.47139736	0.66171803
35607904	GenotypeRiA	0.12515531	0.56574891	0.2212206	0.82937242	0.94177674
35607904	GroupDEX	-0.6794739	0.62274366	-1.0910973	0.30082109	0.7694266
35607904	GenotypeRiA:GroupDEX	0.02661323	0.73671175	0.03612434	0.97189416	0.97189416
**24 Hours**						
**CpG**	**term**	**estimate**	**std.error**	**statistic**	**p.value**	**q.value**
35558386	DissecterLi	-5.3736765	2.4891525	-2.1588378	0.05181881	0.08479441
35558386	column2	-8.9648329	5.42578928	-1.6522634	0.12438597	0.60039288
35558386	column3	-12.965692	6.44044324	-2.0131677	0.06708586	0.71871644
35558386	column9	-11.362393	5.92149814	-1.9188375	0.07909996	0.32354286
35558386	column10	-9.3330367	5.47613549	-1.7043108	0.11405396	0.50017495
35558386	column11	-7.3294834	5.53271108	-1.3247544	0.20992694	0.41985389

35558386	column12	-7.9822985	5.99120879	-1.3323352	0.20749896	0.48871956
35558386	GenotypeRiA	-1.6776031	3.87681739	-0.4327269	0.67288823	0.94283549
35558386	GroupDEX	0.47871193	4.31539972	0.11093107	0.91350479	0.92414001
35558386	GenotypeRiA:GroupDEX	-0.7489985	5.12623812	-0.1461108	0.88625972	0.96511189
35558438	DissecterLi	0.21853638	1.71313301	0.12756533	0.90060574	0.90060574
35558438	column2	-0.4424281	3.73424237	-0.1184787	0.90764857	0.95008097
35558438	column3	-0.5499577	4.43256728	-0.124072	0.90331228	0.95060099
35558438	column9	-1.694044	4.07540877	-0.4156746	0.68498483	0.77060794
35558438	column10	-2.78978	3.76889262	-0.7402121	0.47340569	0.53620824
35558438	column11	1.64928088	3.80783017	0.43312879	0.67260425	0.71216921
35558438	column12	0.4748731	4.1233864	0.1151658	0.91021837	0.95352844
35558438	GenotypeRiA	-2.1219036	2.66817877	-0.7952629	0.44190719	0.94283549
35558438	GroupDEX	-2.9164721	2.97002844	-0.9819677	0.34549992	0.92414001
35558438	GenotypeRiA:GroupDEX	0.51066836	3.52807943	0.14474401	0.88731562	0.96511189
35558488	DissecterLi	1.17258252	1.09033258	1.07543565	0.30173461	0.33945143
35558488	column2	0.65159788	2.39249905	0.27235032	0.78963048	0.88833429
35558488	column3	-0.6559392	2.83937124	-0.2310157	0.8208987	0.92351103
35558488	column9	-1.3059034	2.61102288	-0.5001501	0.62532852	0.75039423

35558488	column10	-2.4729832	2.41248502	-1.0250771	0.32402386	0.53620824
35558488	column11	0.54655033	2.43451089	0.22450108	0.82585748	0.82585748
35558488	column12	-2.4642739	2.56922988	-0.9591489	0.35498371	0.49151591
35558488	GenotypeRiA	-2.5264511	1.70754311	-1.4795826	0.16280593	0.94283549
35558488	GroupDEX	-2.6666596	1.88648171	-1.4135624	0.18098716	0.92414001
35558488	GenotypeRiA:GroupDEX	2.46755924	2.24540975	1.09893495	0.29173226	0.96511189
35558513	DissecterLi	-0.2754776	1.35167817	-0.2038042	0.84166226	0.8911718
35558513	column2	-1.7558622	2.96596543	-0.5920036	0.56400282	0.78092698
35558513	column3	-3.6440069	3.51994997	-1.0352439	0.31942953	0.71871644
35558513	column9	-2.3067253	3.236868	-0.7126411	0.48866239	0.68566842
35558513	column10	-3.2263736	2.99074191	-1.078787	0.30029267	0.53620824
35558513	column11	-1.3787194	3.01804724	-0.456825	0.65533643	0.71216921
35558513	column12	-4.4332649	3.18505749	-1.3918948	0.18731048	0.48871956
35558513	GenotypeRiA	-2.0327304	2.11683003	-0.960271	0.35443979	0.94283549
35558513	GroupDEX	-0.9417966	2.33865905	-0.402708	0.69370704	0.92414001
35558513	GenotypeRiA:GroupDEX	1.48343281	2.78361979	0.53291502	0.60308362	0.96511189

35558566	DissecterLi	-2.2788766	1.10565058	-2.0611183	0.05988308	0.08982462
35558566	column2	-2.0578264	2.42611108	-0.8481996	0.41167257	0.67364603
35558566	column3	-3.2209965	2.87926135	-1.1186885	0.28351877	0.71871644
35558566	column9	-2.504111	2.64770494	-0.9457666	0.36151586	0.65072854
35558566	column10	-2.061945	2.44637783	-0.8428563	0.41454769	0.53620824
35558566	column11	-2.3829818	2.46871314	-0.9652729	0.3520224	0.5280336
35558566	column12	-3.4094562	2.60532479	-1.3086492	0.21331656	0.48871956
35558566	GenotypeRiA	-1.2599866	1.73153225	-0.7276715	0.47971617	0.94283549
35558566	GroupDEX	-0.365668	1.91298475	-0.1911505	0.85136073	0.92414001
35558566	GenotypeRiA:GroupDEX	0.62092531	2.27695534	0.27269982	0.78936762	0.96511189
35558710	DissecterLi	-1.5186618	1.04173276	-1.4578228	0.16862099	0.21279333
35558710	column2	-1.1020613	2.28585724	-0.4821217	0.63773529	0.81994537
35558710	column3	-1.0937442	2.71281083	-0.4031775	0.69337013	0.84004454
35558710	column9	-1.5965411	2.49464073	-0.6399884	0.53329766	0.68566842
35558710	column10	-1.4522947	2.30495237	-0.6300758	0.53956251	0.57130148
35558710	column11	-2.0079676	2.32599647	-0.863272	0.40363395	0.55887777

35558710	column12	-2.8189011	2.45471059	-1.1483639	0.27151086	0.48871956
35558710	GenotypeRiA	-2.9240724	1.63143212	-1.7923347	0.09637012	0.86733111
35558710	GroupDEX	-0.3715073	1.80239482	-0.2061187	0.8398911	0.92414001
35558710	GenotypeRiA:GroupDEX	1.32960925	2.14532422	0.61977077	0.54611889	0.96511189
35558721	DissecterLi	-1.5851454	0.86463676	-1.8333079	0.089748	0.12426646
35558721	column2	-0.630086	1.89725838	-0.3321034	0.74510486	0.88833429
35558721	column3	-0.1422098	2.25162927	-0.0631586	0.95060099	0.95060099
35558721	column9	0.17751079	2.07054838	0.0857313	0.93298635	0.9529167
35558721	column10	-1.502554	1.91310731	-0.7853997	0.44629793	0.53620824
35558721	column11	-1.5426509	1.93057388	-0.7990634	0.43860976	0.56392684
35558721	column12	-2.4175227	2.03740642	-1.1865687	0.25663028	0.48871956
35558721	GenotypeRiA	-3.5000375	1.35408642	-2.5847962	0.02264976	0.40769569
35558721	GroupDEX	-0.5251295	1.49598523	-0.3510259	0.73119229	0.92414001
35558721	GenotypeRiA:GroupDEX	2.43040913	1.78061616	1.36492591	0.19543446	0.96511189
35569751	DissecterLi	17.0241855	4.98648766	3.41406349	0.00461591	0.00999774
35569751	column2	15.1484447	10.9417688	1.38446032	0.18952172	0.60039288
35569751	column3	15.1321623	12.985478	1.16531424	0.26482894	0.71871644

35569751	column9	22.8657813	11.9411578	1.91487138	0.07777222	0.32354286
35569751	column10	15.6860934	11.0331719	1.42172111	0.17865254	0.50017495
35569751	column11	24.5788329	11.1339042	2.20756641	0.04585559	0.18162673
35569751	column12	14.8494278	11.7500232	1.26377859	0.22850191	0.48871956
35569751	GenotypeRiA	-2.2780013	7.80921602	-0.2917068	0.77511365	0.94283549
35569751	GroupDEX	1.29062413	8.62756737	0.14959305	0.88338158	0.92414001
35569751	GenotypeRiA:GroupDEX	2.5206045	10.2690759	0.24545583	0.8099359	0.96511189
35569757	DissecterLi	16.9031356	4.75340505	3.55600574	0.00351574	0.00999774
35569757	column2	14.3976459	10.4303194	1.38036481	0.19074901	0.60039288
35569757	column3	15.3488465	12.3784998	1.23996015	0.23690603	0.71871644
35569757	column9	22.239393	11.3829941	1.95373842	0.07259289	0.32354286
35569757	column10	15.473035	10.5174501	1.47117741	0.1650318	0.50017495
35569757	column11	24.3005904	10.6134739	2.28959818	0.03941183	0.18162673
35569757	column12	14.397147	11.2007937	1.28536846	0.22109083	0.48871956
35569757	GenotypeRiA	-1.0229344	7.44419105	-0.1374138	0.89280922	0.94283549
35569757	GroupDEX	1.33228402	8.22429033	0.1619938	0.87380146	0.92414001
35569757	GenotypeRiA:GroupDEX	2.15356439	9.78907008	0.21999683	0.82929061	0.96511189
35569777	DissecterLi	17.9434686	4.72157615	3.80031329	0.00220602	0.0099271
35569777	column2	15.508217	10.3604778	1.49686311	0.1583092	0.60039288
35569777	column3	16.2874949	12.2956131	1.32465903	0.20809941	0.71871644
35569777	column9	23.1362456	11.3067734	2.04622883	0.06151344	0.32354286

35569777	column10	16.9237721	10.447025	1.6199609	0.12923335	0.50017495
35569777	column11	25.7249481	10.5424058	2.44014018	0.02975907	0.18162673
35569777	column12	15.6221684	11.125793	1.40413977	0.18371491	0.48871956
35569777	GenotypeRiA	-1.5940756	7.39434459	-0.2155804	0.83266036	0.94283549
35569777	GroupDEX	0.81005299	8.16922031	0.09915916	0.92252466	0.92414001
35569777	GenotypeRiA:GroupDEX	3.01278717	9.72352227	0.30984525	0.76158953	0.96511189
35569896	DissecterLi	11.6207965	3.3526919	3.46610928	0.00417696	0.00999774
35569896	column2	9.26110909	7.35675731	1.2588575	0.23021856	0.60039288
35569896	column3	7.43109142	8.73085619	0.85112975	0.41010154	0.72836102
35569896	column9	13.8650268	8.02870191	1.72693256	0.10784762	0.32354286
35569896	column10	10.8798662	7.41821269	1.46664252	0.16624334	0.50017495
35569896	column11	15.5556177	7.4859406	2.07797771	0.05808538	0.18162673
35569896	column12	9.28564608	7.90019153	1.17536974	0.26092529	0.48871956
35569896	GenotypeRiA	-0.7689359	5.25056854	-0.1464481	0.88581428	0.94283549
35569896	GroupDEX	2.53467884	5.80079149	0.43695396	0.66931505	0.92414001
35569896	GenotypeRiA:GroupDEX	1.36204743	6.90446861	0.19727042	0.84666684	0.96511189
35569922	DissecterLi	9.84451236	2.82337994	3.48678271	0.00401453	0.00999774
35569922	column2	6.44950915	6.1952967	1.04103314	0.31683482	0.63366964
35569922	column3	5.7900744	7.35245738	0.78750193	0.44510951	0.72836102
35569922	column9	10.4641436	6.7611569	1.54768536	0.1456899	0.32780228
35569922	column10	8.2392822	6.24704971	1.31890774	0.20996157	0.50017495

35569922	column11	12.9552334	6.30408496	2.05505374	0.06054224	0.18162673
35569922	column12	6.77432555	6.65293531	1.01824612	0.32713768	0.49151591
35569922	GenotypeRiA	-1.174052	4.42162607	-0.2655249	0.7947692	0.94283549
35569922	GroupDEX	1.70027727	4.88498164	0.34806216	0.73336497	0.92414001
35569922	GenotypeRiA:GroupDEX	1.46448239	5.81441385	0.25187103	0.80507877	0.96511189
35570224	DissecterLi	17.5027099	4.41300179	3.96616878	0.00161162	0.0099271
35570224	column2	14.9037376	9.68337806	1.53910521	0.14775811	0.60039288
35570224	column3	15.4895243	11.4920444	1.34784759	0.20072691	0.71871644
35570224	column9	18.8373939	10.5678294	1.78252253	0.0980195	0.32354286
35570224	column10	15.3630304	9.7642691	1.57339277	0.13964106	0.50017495
35570224	column11	23.4186518	9.85341639	2.37670376	0.0335137	0.18162673
35570224	column12	15.1258227	10.3986768	1.4545911	0.16949934	0.48871956
35570224	GenotypeRiA	1.74664918	6.91109386	0.25273122	0.80442812	0.94283549
35570224	GroupDEX	0.74127288	7.63532828	0.09708461	0.92414001	0.92414001
35570224	GenotypeRiA:GroupDEX	0.39230204	9.08805023	0.0431668	0.96622456	0.96622456
35578739	DissecterLi	-2.2691932	0.67283487	-3.3725855	0.00499887	0.00999774
35578739	column2	-1.2706149	1.47639062	-0.8606224	0.40503939	0.67364603
35578739	column3	-1.0720642	1.75215161	-0.6118558	0.55118447	0.82677671
35578739	column9	-1.6007838	1.6112398	-0.9935106	0.33859458	0.65072854

35578739	column10	-1.0910809	1.48872379	-0.7328968	0.47662955	0.53620824
35578739	column11	-2.3816906	1.50231576	-1.5853462	0.13690282	0.30803134
35578739	column12	-1.0785548	1.5854497	-0.6802832	0.50825997	0.60991197
35578739	GenotypeRiA	-0.0770314	1.05371019	-0.0731049	0.94283549	0.94283549
35578739	GroupDEX	0.37440585	1.16413167	0.32161813	0.75285471	0.92414001
35578739	GenotypeRiA:GroupDEX	-1.3241289	1.38562308	-0.9556199	0.35669815	0.96511189
35578830	DissecterLi	-1.679393	0.62921845	-2.6690143	0.01930051	0.03474092
35578830	column2	-0.878187	1.3806838	-0.6360522	0.5357804	0.78092698
35578830	column3	-0.6454384	1.63856863	-0.3939038	0.70003711	0.84004454
35578830	column9	-1.233407	1.50679141	-0.8185652	0.4277851	0.68566842
35578830	column10	-1.0829649	1.39221748	-0.7778705	0.45057091	0.53620824
35578830	column11	-1.6776341	1.40492835	-1.1941065	0.25377046	0.45678683
35578830	column12	-0.3517455	1.48267314	-0.2372374	0.81617027	0.91819156
35578830	GenotypeRiA	-0.4434632	0.98540357	-0.4500321	0.66010019	0.94283549
35578830	GroupDEX	-0.5941157	1.08866699	-0.5457277	0.5944942	0.92414001
35578830	GenotypeRiA:GroupDEX	0.14686276	1.29580025	0.1133375	0.91149456	0.96511189
35578891	DissecterLi	-8.9716965	2.32543279	-3.858076	0.00197703	0.0099271

35578891	column2	-5.9199788	5.10265935	-1.1601752	0.26684128	0.60039288
35578891	column3	-5.1206266	6.05573667	-0.8455828	0.41307897	0.72836102
35578891	column9	-8.6969477	5.56872127	-1.5617495	0.14235341	0.32780228
35578891	column10	-6.5952616	5.14528491	-1.2818069	0.22229998	0.50017495
35578891	column11	-9.6381526	5.1922611	-1.8562534	0.08622093	0.22171096
35578891	column12	-5.5442838	5.47958625	-1.011807	0.33009271	0.49151591
35578891	GenotypeRiA	-0.8811733	3.64180325	-0.2419607	0.81258562	0.94283549
35578891	GroupDEX	-1.0916278	4.02343882	-0.2713171	0.79040773	0.92414001
35578891	GenotypeRiA:GroupDEX	-2.0773313	4.78895115	-0.4337758	0.67156287	0.96511189
35607856	DissecterLi	3.68376369	0.87225497	4.22326476	0.00099548	0.0099271
35607856	column2	0.12215841	1.9139749	0.06382446	0.95008097	0.95008097
35607856	column3	0.92802252	2.27146811	0.40855626	0.68951535	0.84004454
35607856	column9	1.36192306	2.08879175	0.65201477	0.5257524	0.68566842
35607856	column10	1.57538468	1.92996348	0.81627694	0.42904615	0.53620824
35607856	column11	2.1449843	1.94758395	1.10135653	0.29071585	0.47571684
35607856	column12	-0.1221106	2.05535777	-0.0594109	0.95352844	0.95352844
35607856	GenotypeRiA	1.36299433	1.36601712	0.99778715	0.33659341	0.94283549
35607856	GroupDEX	0.56692324	1.50916618	0.37565329	0.71323301	0.92414001

35607856	GenotypeRiA:GroupDEX	-0.4945523	1.79630496	-0.2753165	0.78740049	0.96511189
35607904	DissecterLi	0.63994076	0.44864354	1.42639022	0.17732777	0.21279333
35607904	column2	-1.1904768	0.98445123	-1.2092796	0.24808914	0.60039288
35607904	column3	-1.3036609	1.16832753	-1.1158351	0.28469426	0.71871644
35607904	column9	-0.0646704	1.07436811	-0.0601939	0.9529167	0.9529167
35607904	column10	-0.2566478	0.99267494	-0.2585416	0.8000372	0.8000372
35607904	column11	-0.452994	1.00173801	-0.452208	0.65857255	0.71216921
35607904	column12	-0.867678	1.05717138	-0.8207543	0.42658095	0.54846123
35607904	GenotypeRiA	0.62323627	0.70260965	0.88703062	0.39117761	0.94283549
35607904	GroupDEX	0.50604539	0.77623824	0.65192021	0.52581149	0.92414001
35607904	GenotypeRiA:GroupDEX	0.63604396	0.9239278	0.68841306	0.50329306	0.96511189

Supplementary Table 16. Results of multiple linear regression models on DNA methylation for each investigated CpG in the hippocampus of the humanized *FKBP5* mouse at baseline, after four and 24 hours of dexamethasone treatment.

Baseline						
**CpG**	**term**	**estimate**	**std.error**	**statistic**	**p.value**	**q.value**
35558386	DissecterLi	5.64666667	1.99402626	2.83179152	0.06609196	0.20561945
35558386	column2	-7.2566667	3.15283235	-2.3016342	0.10483118	0.29343929

35558386	column3	-8.025	2.11498424	-3.7943545	0.03212769	0.13588125
35558386	column4	-13.896667	3.15283235	-4.407677	0.02166193	0.1059758
35558386	column6	-2.08	2.44217344	-0.8517004	0.45696896	0.69138782
35558386	column7	-11.22	2.44217344	-4.5942683	0.01937755	0.10519212
35558386	GenotypeRiA	NA	NA	NA	NA	NA
35558438	DissecterLi	-1.1033333	2.11690725	-0.5212006	0.63825599	0.77019822
35558438	column2	9.90333333	3.34712425	2.9587588	0.05960338	0.19073082
35558438	column3	3.225	2.24531921	1.43632139	0.24643756	0.48387146
35558438	column4	2.35333333	3.34712425	0.70309112	0.53265006	0.71800525
35558438	column6	4.58	2.5926713	1.7665178	0.17548029	0.39307584
35558438	column7	4.98	2.5926713	1.92079883	0.15051949	0.36648223
35558438	GenotypeRiA	NA	NA	NA	NA	NA
35558488	DissecterLi	-2.575	0.81132265	-3.1738298	0.05033216	0.1708243
35558488	column2	4.345	1.28281375	3.38708562	0.0428668	0.1548736
35558488	column3	0.505	0.86053762	0.58684244	0.59854795	0.75265269
35558488	column4	-1.685	1.28281375	-1.3135188	0.28043169	0.50658629
35558488	column6	0.485	0.99366326	0.48809292	0.65893331	0.77019822
35558488	column7	-0.27	0.99366326	-0.2717218	0.8034622	0.85702635
35558488	GenotypeRiA	NA	NA	NA	NA	NA
35558513	DissecterLi	2.35333333	0.85151608	2.76369806	0.06993571	0.21169728
35558513	column2	-2.8883333	1.34636513	-2.1452823	0.12124656	0.30862761

35558513	column3	-0.7425	0.90316919	-0.8221051	0.47126835	0.69201927
35558513	column4	-6.2833333	1.34636513	-4.6668865	0.01857325	0.10519212
35558513	column6	-1.43	1.04288995	-1.3711897	0.26388228	0.48546846
35558513	column7	-5.395	1.04288995	-5.1731249	0.01401747	0.0872198
35558513	GenotypeRiA	NA	NA	NA	NA	NA
35558566	DissecterLi	1.57	1.03662915	1.51452426	0.22712633	0.4625118
35558566	column2	0.75	1.63905461	0.45758085	0.67835898	0.77203508
35558566	column3	0.765	1.09951126	0.69576368	0.53663198	0.71800525
35558566	column4	-1.42	1.63905461	-0.8663531	0.45003072	0.69045809
35558566	column6	1.18	1.26960624	0.92942202	0.42122728	0.67490014
35558566	column7	-0.43	1.26960624	-0.3386877	0.7571647	0.82332473
35558566	GenotypeRiA	NA	NA	NA	NA	NA
35558710	DissecterLi	6.94	1.82552884	3.8016381	0.03196898	0.13588125
35558710	column2	-7.46	2.88641454	-2.5845213	0.08145979	0.240092
35558710	column3	-3.005	1.93626574	-1.5519564	0.21847614	0.45313569
35558710	column4	-10.87	2.88641454	-3.7659178	0.03275709	0.13588125
35558710	column6	-2.06	2.23580709	-0.9213675	0.42481053	0.67490014
35558710	column7	-8.01	2.23580709	-3.5825989	0.03721774	0.14887096
35558710	GenotypeRiA	NA	NA	NA	NA	NA
35558721	DissecterLi	7.89	1.49776129	5.26786214	0.01333253	0.0872198
35558721	column2	-9.61	2.36816854	-4.0579882	0.02697117	0.12586546
35558721	column3	-0.52	1.58861575	-0.327329	0.76492981	0.82377056

35558721	column4	-10.42	2.36816854	-4.4000247	0.02176289	0.1059758
35558721	column6	1.27	1.83437546	0.69233373	0.53850394	0.71800525
35558721	column7	-6.32	1.83437546	-3.4453143	0.04107658	0.15335256
35558721	GenotypeRiA	NA	NA	NA	NA	NA
35569751	DissecterLi	-8.8233333	4.6868097	-1.8825883	0.15629463	0.37244677
35569751	column2	4.57333333	7.41049681	0.61714261	0.58081698	0.74217941
35569751	column3	0.345	4.97111238	0.06940097	0.94903752	0.96846375
35569751	column4	12.9033333	7.41049681	1.74122379	0.18001323	0.39526745
35569751	column6	-4.59	5.74014615	-0.7996312	0.48238313	0.6926527
35569751	column7	7.86	5.74014615	1.36930312	0.26440693	0.48546846
35569751	GenotypeRiA	NA	NA	NA	NA	NA
35569757	DissecterLi	-7.0933333	4.99379467	-1.4204295	0.25057629	0.48387146
35569757	column2	5.06333333	7.89588266	0.64126248	0.56697958	0.74217941
35569757	column3	0.58	5.29671911	0.10950175	0.91971841	0.96269591
35569757	column4	10.9933333	7.89588266	1.39228682	0.25809064	0.48546846
35569757	column6	-3.75	6.11612441	-0.6131334	0.58314096	0.74217941
35569757	column7	7.01	6.11612441	1.14615066	0.33486753	0.57700251
35569757	GenotypeRiA	NA	NA	NA	NA	NA
35569777	DissecterLi	-8.1133333	4.4416872	-1.8266332	0.16522345	0.38552139
35569777	column2	5.89333333	7.0229241	0.83915663	0.46298291	0.69138782
35569777	column3	2.015	4.71112071	0.42771139	0.69770584	0.78143054

35569777	column4	11.4433333	7.0229241	1.62942574	0.20171275	0.4262609
35569777	column6	-3.87	5.43993362	-0.7114057	0.52816004	0.71800525
35569777	column7	7.89	5.43993362	1.45038535	0.24283661	0.48387146
35569777	GenotypeRiA	NA	NA	NA	NA	NA
35569896	DissecterLi	-7.36	1.61254113	-4.5642247	0.01972352	0.10519212
35569896	column2	8.28	2.54965139	3.24750279	0.04757947	0.16652813
35569896	column3	2.175	1.71035815	1.27166348	0.29312553	0.52111206
35569896	column4	8.85	2.54965139	3.47106276	0.0403153	0.15335256
35569896	column6	-1.78	1.97495148	-0.901288	0.43386438	0.67490014
35569896	column7	6.08	1.97495148	3.07855665	0.05419721	0.178532
35569896	GenotypeRiA	NA	NA	NA	NA	NA
35569922	DissecterLi	-4.47	1.96475613	-2.2750915	0.10741974	0.29343929
35569922	column2	5.35	3.10655222	1.72216645	0.18351703	0.39526745
35569922	column3	2.41	2.08393858	1.15646403	0.33122066	0.57700251
35569922	column4	6.78	3.10655222	2.18248384	0.117076	0.30862761
35569922	column6	0.16	2.406325	0.06649143	0.95116976	0.96846375
35569922	column7	4.32	2.406325	1.79526872	0.17048603	0.38968235
35569922	GenotypeRiA	NA	NA	NA	NA	NA
35570224	DissecterLi	-3.28	3.08334595	-1.0637794	0.3654613	0.61092039
35570224	column2	2.2	4.875198	0.45126372	0.68242387	0.77203508
35570224	column3	3.05	3.27038224	0.9326127	0.41981548	0.67490014
35570224	column4	2.37	4.875198	0.4861341	0.6601699	0.77019822
35570224	column6	-3.07	3.77631213	-0.8129625	0.47576325	0.69201927
35570224	column7	1.75	3.77631213	0.46341508	0.6746178	0.77203508
35570224	GenotypeRiA	NA	NA	NA	NA	NA

35578891	Dissecter Li	3.28	1.52668122	2.14845113	0.12088446	0.30862761
35578891	column2	-1.51	2.41389496	-0.625545	0.57596851	0.74217941
35578891	column3	-0.88	1.61928997	-0.5434481	0.624603	0.76874215
35578891	column4	-0.13	2.41389496	-0.0538549	0.9604365	0.96908908
35578891	column6	0.91	1.869795	0.48668437	0.65982237	0.77019822
35578891	column7	-1.31	1.869795	-0.7006116	0.53399489	0.71800525
35578891	GenotypeRiA	NA	NA	NA	NA	NA
35607856	Dissecter Li	1.09	1.38846838	0.78503768	0.48971926	0.69428554
35607856	column2	1.1	2.19536127	0.50105648	0.65078644	0.77019822
35607856	column3	-0.105	1.47269311	-0.071298	0.94764762	0.96846375
35607856	column4	0.08	2.19536127	0.03644047	0.97322032	0.97322032
35607856	column6	0.68	1.70051953	0.3998778	0.71601612	0.79399808
35607856	column7	0.98	1.70051953	0.57629447	0.60481019	0.75265269
35607856	GenotypeRiA	NA	NA	NA	NA	NA
35607904	Dissecter Li	1.40333333	0.71655555	1.95844319	0.14507245	0.36106921
35607904	column2	-0.4033333	1.1329738	-0.3559953	0.74540667	0.81848576
35607904	column3	-0.15	0.76002193	-0.1973627	0.85615897	0.9046208
35607904	column4	-2.7333333	1.1329738	-2.41253	0.09480158	0.27225069
35607904	column6	0.8	0.87759773	0.91157938	0.42920238	0.67490014
35607904	column7	-0.95	0.87759773	-1.0825005	0.35827526	0.60798226
35607904	GenotypeRiA	NA	NA	NA	NA	NA
**4 Hours**						
**CpG**	**term**	**estimate**	**std.error**	**statistic**	**p.value**	**q.value**

35558386	DissecterLi	4.75584774	2.81295351	1.69069547	0.12936094	0.80475861
35558386	column2	-2.2566733	3.94684267	-0.5717667	0.58318388	0.82853972
35558386	column3	0.14524915	5.09283162	0.02852031	0.97794581	0.99115085
35558386	column4	-1.2574218	4.42246573	-0.284326	0.78338128	0.98622825
35558386	column5	-2.6893535	4.15599686	-0.6471019	0.53568804	0.75095812
35558386	column6	-1.0538875	3.73964941	-0.2818145	0.78523844	0.78523844
35558386	column7	-3.4185323	4.3972122	-0.7774317	0.45927429	0.56526067
35558386	column8	3.9770449	6.00710372	0.66205697	0.52654219	0.561645
35558386	GenotypeRiA	4.37804064	4.09204058	1.06989179	0.31588294	0.64056946
35558386	GroupDEX	4.68555086	3.21078299	1.45931721	0.18259798	0.32461863
35558386	GenotypeRiA:GroupDEX	-1.2238356	4.86133927	-0.2517486	0.8075819	0.98333491
35558438	DissecterLi	1.29437657	1.89544637	0.68288746	0.51396349	0.88479037
35558438	column2	7.28804636	2.65949245	2.74038994	0.0254339	0.40694236
35558438	column3	6.7566715	3.4316917	1.96890399	0.08448675	0.24067283
35558438	column4	5.59215068	2.97998051	1.8765729	0.09741762	0.75300934
35558438	column5	6.03812376	2.80042637	2.15614445	0.06316772	0.25750998
35558438	column6	5.54256058	2.51987987	2.19953366	0.05903682	0.15895327
35558438	column7	5.80217619	2.96296398	1.95823379	0.08589225	0.41540839
35558438	column8	6.04978669	4.0477537	1.49460346	0.17337641	0.27740226
35558438	GenotypeRiA	1.87976778	2.75733084	0.68173458	0.51465476	0.70913213

35558438	GroupDEX	0.11646259	2.16351495	0.05383027	0.95839038	0.95839038
35558438	GenotypeRiA:GroupDEX	-0.4418485	3.27570572	-0.1348865	0.89603359	0.98333491
35558488	DissecterLi	0.23333949	1.51899579	0.15361431	0.8817181	0.94721008
35558488	column2	4.10685469	2.1312963	1.92692808	0.09014696	0.48078381
35558488	column3	2.1520658	2.75013069	0.78253219	0.45643787	0.56176968
35558488	column4	4.20152197	2.3881329	1.75933339	0.11656506	0.75300934
35558488	column5	4.81157409	2.24423963	2.14396628	0.0643775	0.25750998
35558488	column6	2.29412697	2.01941187	1.13603718	0.28882537	0.33008614
35558488	column7	3.37483859	2.37449599	1.42128629	0.19302054	0.41540839
35558488	column8	3.46088688	3.24383792	1.06691116	0.3171478	0.42286374
35558488	GenotypeRiA	0.8658418	2.20970321	0.39183624	0.70541039	0.8681974
35558488	GroupDEX	0.33680037	1.73382383	0.19425294	0.85081988	0.94015737
35558488	GenotypeRiA:GroupDEX	-1.3806854	2.62512475	-0.5259504	0.61318646	0.98333491
35558513	DissecterLi	1.67384879	1.05389467	1.58825056	0.15089224	0.80475861
35558513	column2	3.07383516	1.47871497	2.07872053	0.07125676	0.48078381
35558513	column3	3.07793662	1.90806854	1.61311638	0.14538373	0.25845996
35558513	column4	2.60455337	1.6569108	1.57193336	0.15460951	0.75300934
35558513	column5	3.3524878	1.55707619	2.15306599	0.0634714	0.25750998
35558513	column6	2.10548665	1.40108842	1.50275074	0.17130713	0.21083954
35558513	column7	2.90791617	1.64744937	1.76510199	0.11554536	0.41540839
35558513	column8	4.80067313	2.25060762	2.13305647	0.06548061	0.13585088
35558513	GenotypeRiA	0.02404209	1.53311448	0.01568186	0.98787224	0.98787224

35558513	GroupDEX	1.04643093	1.20294454	0.86989125	0.40970024	0.58495239
35558513	GenotypeRiA:GroupDEX	0.13476118	1.82133815	0.0739902	0.94283479	0.98333491
35558566	DissecterLi	1.01255113	0.7904234	1.28102373	0.23607021	0.88479037
35558566	column2	1.20046004	1.10903959	1.08243209	0.31060472	0.82827926
35558566	column3	1.13007015	1.43105574	0.78967584	0.45248504	0.56176968
35558566	column4	1.78754849	1.24268687	1.43845447	0.18825234	0.75300934
35558566	column5	2.87324911	1.16781069	2.46037234	0.03929594	0.25750998
35558566	column6	2.15197695	1.0508195	2.04790352	0.07475109	0.15895327
35558566	column7	1.56443352	1.23559078	1.26614212	0.24108513	0.41540839
35558566	column8	4.09751308	1.68796083	2.42749299	0.0413637	0.12986784
35558566	GenotypeRiA	0.9866328	1.14983935	0.85806144	0.41582432	0.66531892
35558566	GroupDEX	1.08325307	0.90221114	1.2006647	0.2642231	0.42275697
35558566	GenotypeRiA:GroupDEX	-1.5495309	1.36600776	-1.13435	0.28949167	0.98333491
35558710	DissecterLi	0.02491885	1.41697189	0.01758599	0.98639982	0.98639982
35558710	column2	-0.324085	1.98814702	-0.1630086	0.87455419	0.90566479
35558710	column3	-4.4597242	2.56541718	-1.7384012	0.12033642	0.24067283
35558710	column4	-1.7055931	2.2277331	-0.7656182	0.46588919	0.98622825
35558710	column5	-1.1896728	2.09350447	-0.5682686	0.58544559	0.75095812
35558710	column6	-3.2031376	1.88377735	-1.7003802	0.1274795	0.21083954
35558710	column7	-2.92323	2.21501211	-1.3197354	0.22343689	0.41540839

35558710	column8	-3.785795	3.02596439	-1.2511036	0.24624369	0.35817265
35558710	GenotypeRiA	1.34676343	2.06128771	0.65336024	0.5318491	0.70913213
35558710	GroupDEX	1.13109513	1.61737094	0.69934182	0.5041607	0.62050548
35558710	GenotypeRiA:GroupDEX	-2.3790025	2.44880731	-0.9714944	0.35975502	0.98333491
35558721	DissecterLi	0.21263227	1.46262828	0.14537684	0.88800945	0.94721008
35558721	column2	1.42787835	2.0522073	0.69577686	0.5062745	0.82853972
35558721	column3	-3.7918723	2.64807773	-1.4319339	0.19005098	0.30408157
35558721	column4	-1.5868584	2.29951311	-0.6900845	0.50966123	0.98622825
35558721	column5	-0.4320781	2.16095948	-0.1999473	0.84651081	0.84651081
35558721	column6	-3.0782188	1.94447472	-1.5830593	0.15206598	0.21083954
35558721	column7	-2.7740032	2.28638223	-1.2132718	0.25963024	0.41540839
35558721	column8	-1.7983586	3.12346428	-0.5757577	0.5806095	0.5806095
35558721	GenotypeRiA	0.09986938	2.12770466	0.04693761	0.96371353	0.98787224
35558721	GroupDEX	0.25715794	1.6694844	0.15403435	0.88139753	0.94015737
35558721	GenotypeRiA:GroupDEX	0.05447157	2.52771057	0.02154976	0.98333491	0.98333491
35569751	DissecterLi	-0.3358989	1.62686099	-0.2064706	0.84158132	0.94721008
35569751	column2	-1.0121491	1.97065291	-0.513611	0.62140479	0.82853972
35569751	column3	4.69055635	2.67304823	1.75475934	0.1173796	0.24067283

35569751	column4	-0.5075854	2.27021534	-0.2235847	0.82868428	0.98622825
35569751	column5	1.13464791	2.13872146	0.53052627	0.61015347	0.75095812
35569751	column6	4.68341758	1.97320944	2.37350253	0.04499973	0.15895327
35569751	column7	1.8732316	2.292314	0.81717932	0.43748341	0.56526067
35569751	column8	8.44119059	3.38534048	2.49345395	0.0373208	0.12986784
35569751	GenotypeRiA	2.88371006	2.06882921	1.39388503	0.20085036	0.64056946
35569751	GroupDEX	5.72755842	1.7990296	3.18369327	0.01292544	0.0413614
35569751	GenotypeRiA:GroupDEX	-0.46701	2.48405669	-0.188003	0.8555555	0.98333491
35569757	DissecterLi	0.44325703	1.43430958	0.30903861	0.76518736	0.94721008
35569757	column2	-0.90842	1.7374111	-0.5228584	0.61524044	0.82853972
35569757	column3	5.14893022	2.35667257	2.18483054	0.06040587	0.24067283
35569757	column4	0.79222381	2.00151803	0.39581148	0.70259045	0.98622825
35569757	column5	1.39502464	1.88558745	0.73983555	0.48054449	0.75095812
35569757	column6	4.35438086	1.73966504	2.50299958	0.03676978	0.15895327
35569757	column7	3.26804324	2.02100115	1.61704175	0.14453112	0.41540839
35569757	column8	8.07191067	2.9846596	2.70446609	0.02688746	0.12986784
35569757	GenotypeRiA	2.64292322	1.82396749	1.4489969	0.18537602	0.64056946
35569757	GroupDEX	5.52303767	1.58610072	3.48214815	0.00829343	0.0413614
35569757	GenotypeRiA:GroupDEX	-1.0245207	2.19004963	-0.4678071	0.65240194	0.98333491
35569777	DissecterLi	0.95505961	1.54236488	0.61921768	0.55299398	0.88479037
35569777	column2	-1.2115816	1.86830089	-0.6484938	0.53483277	0.82853972

35569777	column3	5.73212049	2.53421511	2.26189184	0.05356398	0.24067283
35569777	column4	0.51093626	2.15230461	0.23739031	0.81832046	0.98622825
35569777	column5	1.49506756	2.02764027	0.73734359	0.48197677	0.75095812
35569777	column6	4.23921634	1.87072464	2.26608249	0.05321481	0.15895327
35569777	column7	3.12968844	2.17325551	1.44009226	0.18780293	0.41540839
35569777	column8	9.72093785	3.20951224	3.02878978	0.01633947	0.12986784
35569777	GenotypeRiA	2.00511206	1.9613781	1.02229757	0.33655628	0.64056946
35569777	GroupDEX	5.49252265	1.70559137	3.22030396	0.01223361	0.0413614
35569777	GenotypeRiA:GroupDEX	-0.7527182	2.35503944	-0.3196202	0.7574434	0.98333491
35569896	DissecterLi	1.10916071	1.07380404	1.03292655	0.33185096	0.88479037
35569896	column2	-0.8437307	1.30072272	-0.648663	0.53472887	0.82853972
35569896	column3	4.74601017	1.76433635	2.68996904	0.02749795	0.24067283
35569896	column4	0.02668415	1.49844788	0.01780786	0.98622825	0.98622825
35569896	column5	1.94638213	1.41165579	1.37879371	0.20527987	0.54741297
35569896	column6	3.61263392	1.30241015	2.77380664	0.02415433	0.15895327
35569896	column7	2.62625338	1.51303403	1.73575302	0.12082158	0.41540839
35569896	column8	7.93546177	2.23448242	3.5513646	0.0074945	0.11991207
35569896	GenotypeRiA	3.14882213	1.36552365	2.30594477	0.05000463	0.64056946
35569896	GroupDEX	4.67008107	1.18744334	3.9328875	0.00433904	0.0413614
35569896	GenotypeRiA:GroupDEX	-1.9797282	1.63959313	-1.2074509	0.26174259	0.98333491
35569922	DissecterLi	0.5761278	0.91643374	0.6286628	0.54709539	0.88479037

35569922	column2	-1.6192577	1.11009658	-1.4586638	0.18277277	0.58487287
35569922	column3	2.84931966	1.50576577	1.89227284	0.09509265	0.24067283
35569922	column4	-0.6457527	1.27884432	-0.5049502	0.6272072	0.98622825
35569922	column5	0.44694484	1.20477196	0.37097879	0.72028478	0.82318261
35569922	column6	2.4101065	1.11153671	2.16826533	0.06198587	0.15895327
35569922	column7	0.26930536	1.29129281	0.20855483	0.84000782	0.89600834
35569922	column8	4.42974408	1.90701004	2.32287402	0.04870044	0.12986784
35569922	GenotypeRiA	1.87752027	1.16540067	1.6110513	0.14583412	0.64056946
35569922	GroupDEX	3.28332856	1.01341875	3.23985379	0.01188023	0.0413614
35569922	GenotypeRiA:GroupDEX	-0.7494278	1.39930417	-0.5355717	0.60681848	0.98333491
35570224	DissecterLi	3.03385312	1.36485808	2.22283412	0.05692986	0.80475861
35570224	column2	-2.5409029	1.65328296	-1.5368832	0.16287633	0.58487287
35570224	column3	4.90013511	2.24255883	2.18506424	0.06038386	0.24067283
35570224	column4	1.68825306	1.90460143	0.88640754	0.40125782	0.98622825
35570224	column5	3.49903354	1.79428439	1.95009975	0.08697878	0.27833208
35570224	column6	2.52321094	1.65542776	1.5242048	0.16596343	0.21083954
35570224	column7	3.49492767	1.92314114	1.81730171	0.10669188	0.41540839
35570224	column8	5.99128914	2.84013775	2.10950653	0.06792544	0.13585088
35570224	GenotypeRiA	-1.6859561	1.73564815	-0.9713698	0.35981337	0.64056946
35570224	GroupDEX	2.52826419	1.50929926	1.67512451	0.13243993	0.287061

35570224	GenotypeRiA:GroupDEX	1.08829757	2.08400403	0.52221471	0.61566847	0.98333491
35578891	DissecterLi	0.20809728	0.80868283	0.25732868	0.80342016	0.94721008
35578891	column2	0.11981879	0.9795755	0.12231705	0.90566479	0.90566479
35578891	column3	-1.0602194	1.32872336	-0.7979233	0.44795028	0.56176968
35578891	column4	-0.119752	1.1284825	-0.1061178	0.91810162	0.98622825
35578891	column5	-0.9994897	1.0631193	-0.9401483	0.37465741	0.75095812
35578891	column6	-1.9699189	0.9808463	-2.008387	0.07947663	0.15895327
35578891	column7	-0.3485885	1.13946734	-0.3059223	0.7674734	0.87711245
35578891	column8	-2.7752694	1.6827908	-1.6492064	0.13771546	0.24482749
35578891	GenotypeRiA	-1.2039771	1.02837714	-1.1707544	0.27538749	0.64056946
35578891	GroupDEX	-1.546123	0.89426469	-1.7289322	0.12207963	0.287061
35578891	GenotypeRiA:GroupDEX	0.43476395	1.23477912	0.35209856	0.73385951	0.98333491
35607856	DissecterLi	-0.6042486	0.88708919	-0.6811588	0.51291407	0.88479037
35607856	column2	0.38101183	1.18965321	0.32027134	0.75607333	0.90566479
35607856	column3	0.01857253	1.62877064	0.01140279	0.99115085	0.99115085
35607856	column4	0.08491779	1.41806662	0.0598828	0.95355761	0.98622825
35607856	column5	-0.891903	1.33277651	-0.6692068	0.52015921	0.75095812
35607856	column6	-1.2888414	1.23020441	-1.0476645	0.32211234	0.34358649

35607856	column7	0.06979026	1.42846233	0.04885691	0.96210038	0.96210038
35607856	column8	-1.4698909	1.97761543	-0.7432643	0.4762751	0.5443144
35607856	GenotypeRiA	0.19539874	1.2674196	0.15417052	0.88087706	0.98787224
35607856	GroupDEX	-0.8123256	1.00260595	-0.8102142	0.43871429	0.58495239
35607856	GenotypeRiA:GroupDEX	-0.0958612	1.5509119	-0.0618096	0.95206534	0.98333491
35607904	DissecterLi	0.607941	0.67682457	0.89822536	0.39246116	0.88479037
35607904	column2	0.16182852	0.90767258	0.17828953	0.86244422	0.90566479
35607904	column3	0.59757222	1.24270706	0.4808633	0.64208775	0.73381457
35607904	column4	0.84184081	1.08194571	0.77808046	0.45648842	0.98622825
35607904	column5	-0.2104702	1.0168717	-0.2069781	0.84063402	0.84651081
35607904	column6	-1.4227658	0.93861202	-1.5158189	0.16387009	0.21083954
35607904	column7	1.06394899	1.08987734	0.97620984	0.35446839	0.51559038
35607904	column8	-1.1913215	1.50886601	-0.7895475	0.45009162	0.5443144
35607904	GenotypeRiA	-0.9320129	0.96700619	-0.9638128	0.36032032	0.64056946
35607904	GroupDEX	-1.2257744	0.76496067	-1.6024019	0.1435305	0.287061
35607904	GenotypeRiA:GroupDEX	0.4150799	1.18330299	0.35078074	0.73382358	0.98333491
**24 Hours**						
**CpG**	**term**	**estimate**	**std.error**	**statistic**	**p.value**	**q.value**
35558386	DissecterLi	2.07318711	1.21510542	1.70617881	0.1136977	0.18191632

35558386	column2	2.71844168	2.10547533	1.29112968	0.22097705	0.8586917
35558386	column3	-1.3831059	2.02680755	-0.6824061	0.50794138	0.98613518
35558386	column4	-3.5365073	1.9143471	-1.8473699	0.0894834	0.71586717
35558386	column5	-3.1115842	2.1640936	-1.4378233	0.17604719	0.35209437
35558386	column6	0.41159356	1.67317416	0.24599565	0.80984284	0.81223671
35558386	column7	1.28793902	2.51817325	0.51145767	0.61831085	0.99510151
35558386	column8	-0.6120085	2.97028983	-0.2060434	0.84021134	0.94105105
35558386	GenotypeRiA	-4.0355904	2.17551125	-1.8550078	0.08831727	0.64679873
35558386	GroupDEX	-0.7085603	1.72532068	-0.4106833	0.68854314	0.98647054
35558386	GenotypeRiA:GroupDEX	4.07350784	2.57871247	1.57966733	0.14016754	0.32038294
35558438	DissecterLi	-0.2545146	0.91157803	-0.2792022	0.78484333	0.78508226
35558438	column2	-1.2174293	1.5795379	-0.7707503	0.45576277	0.8586917
35558438	column3	-3.0276199	1.52052094	-1.9911728	0.06972589	0.98613518
35558438	column4	-4.4935824	1.43615257	-3.1289033	0.00870958	0.13935332
35558438	column5	-5.3473024	1.62351361	-3.2936604	0.00641566	0.10265053
35558438	column6	-3.6997573	1.25522344	-2.947489	0.01220101	0.19521624
35558438	column7	0.21649342	1.8891459	0.11459857	0.91065848	0.99510151

35558438	column8	-3.3003612	2.22832597	-1.4810944	0.16435515	0.86477326
35558438	GenotypeRiA	-4.710786	1.63207919	-2.886371	0.01366793	0.21868695
35558438	GroupDEX	-0.5246973	1.29434402	-0.405377	0.69233453	0.98647054
35558438	GenotypeRiA:GroupDEX	4.21655185	1.93456272	2.17958912	0.04993055	0.19972219
35558488	DissecterLi	-0.7799094	1.43139689	-0.5448589	0.59583163	0.68095043
35558488	column2	1.63236319	2.48025464	0.65814339	0.52287209	0.8586917
35558488	column3	-0.4730057	2.38758381	-0.1981106	0.84627499	0.98646664
35558488	column4	1.48684794	2.25510516	0.65932532	0.52213889	0.99655376
35558488	column5	0.33942021	2.54930709	0.13314215	0.89628763	0.93691101
35558488	column6	2.9575453	1.97100289	1.50052814	0.15932249	0.71821239
35558488	column7	0.10792877	2.96641371	0.03638359	0.97157474	0.99510151
35558488	column8	4.18521591	3.49900805	1.19611497	0.25475139	0.86477326
35558488	GenotypeRiA	-0.1978801	2.56275711	-0.0772137	0.939726	0.939726
35558488	GroupDEX	0.03518985	2.03243161	0.01731416	0.98647054	0.98647054
35558488	GenotypeRiA:GroupDEX	-1.279977	3.03772905	-0.4213598	0.68094148	0.70415917
35558513	DissecterLi	-0.4156918	1.49055799	-0.2788833	0.78508226	0.78508226
35558513	column2	0.93301875	2.58276609	0.36124787	0.72419431	0.89131608
35558513	column3	-2.2009504	2.48626509	-0.8852437	0.3934183	0.98613518

35558513	column4	-1.1924323	2.34831096	-0.507783	0.62080936	0.99655376
35558513	column5	-0.9484603	2.65467255	-0.3572796	0.72708703	0.86102361
35558513	column6	0.49840411	2.05246646	0.24283179	0.81223671	0.81223671
35558513	column7	0.55877993	3.08901861	0.18089238	0.85947125	0.99510151
35558513	column8	1.04889715	3.64362562	0.28787182	0.77835621	0.94105105
35558513	GenotypeRiA	-0.6576539	2.66867848	-0.2464343	0.8095111	0.90739683
35558513	GroupDEX	0.27759816	2.11643409	0.13116315	0.89781957	0.98647054
35558513	GenotypeRiA:GroupDEX	-1.2302154	3.16328149	-0.3889048	0.70415917	0.70415917
35558566	DissecterLi	0.83253632	1.41944984	0.58652042	0.56839539	0.68095043
35558566	column2	0.52312473	2.45955337	0.21269095	0.83513824	0.95444371
35558566	column3	-0.2111303	2.36765601	-0.0891727	0.93041566	0.98646664
35558566	column4	-0.519056	2.23628309	-0.2321066	0.82036637	0.99655376
35558566	column5	0.20433608	2.52802948	0.0808282	0.93691101	0.93691101
35558566	column6	0.74126816	1.95455206	0.37925219	0.71112632	0.81223671
35558566	column7	0.35477006	2.94165475	0.12060221	0.90600192	0.99510151
35558566	column8	2.54960539	3.46980383	0.73479814	0.47657732	0.94105105
35558566	GenotypeRiA	-0.4888329	2.54136724	-0.1923503	0.85068453	0.90739683
35558566	GroupDEX	0.86555228	2.01546807	0.42945472	0.67520218	0.98647054
35558566	GenotypeRiA:GroupDEX	-1.9303876	3.01237486	-0.6408192	0.53368707	0.70415917

35558710	DissecterLi	2.16624191	1.51523345	1.42964235	0.17833472	0.25939596
35558710	column2	-1.1508137	2.62552253	-0.438318	0.66894251	0.89131608
35558710	column3	0.04377283	2.527424	0.01731915	0.98646664	0.98646664
35558710	column4	-0.1524195	2.38718611	-0.063849	0.95014173	0.99655376
35558710	column5	-1.6707741	2.69861937	-0.6191218	0.54741031	0.79623317
35558710	column6	1.15812095	2.08644404	0.55506926	0.58904473	0.78539297
35558710	column7	1.38203761	3.14015582	0.44011753	0.66767471	0.99510151
35558710	column8	1.41348519	3.70394408	0.38161623	0.70941743	0.94105105
35558710	GenotypeRiA	-3.0684036	2.71285716	-1.1310598	0.28012845	0.75204263
35558710	GroupDEX	-0.3935884	2.15147063	-0.1829392	0.85790011	0.98647054
35558710	GenotypeRiA:GroupDEX	2.9071408	3.21564808	0.90406062	0.38375387	0.55818745
35558721	DissecterLi	2.44983748	1.37631223	1.78000125	0.10038891	0.17846917
35558721	column2	-0.2280681	2.38480662	-0.0956338	0.92538992	0.96731394
35558721	column3	0.27473644	2.29570206	0.11967426	0.90672142	0.98646664
35558721	column4	0.00956242	2.1683216	0.00441006	0.99655376	0.99655376
35558721	column5	-1.6511624	2.45120171	-0.6736135	0.51332286	0.79623317
35558721	column6	1.74991874	1.89515249	0.92336567	0.37401056	0.71821239
35558721	column7	2.10476665	2.85225675	0.73793029	0.47474084	0.99510151
35558721	column8	3.65451352	3.36435518	1.08624486	0.29870942	0.86477326

35558721	GenotypeRiA	-2.3712628	2.46413414	-0.9623107	0.35488467	0.75204263
35558721	GroupDEX	-1.896531	1.95421723	-0.9704812	0.35096204	0.92436616
35558721	GenotypeRiA:GroupDEX	3.59678412	2.92082765	1.23142635	0.24175146	0.39879274
35569751	DissecterLi	-5.4474903	1.5029748	-3.6244721	0.00348587	0.01544397
35569751	column2	1.03672641	2.60428134	0.39808541	0.6975586	0.89131608
35569751	column3	1.9722226	2.50697645	0.78669371	0.4467197	0.98613518
35569751	column4	0.50015335	2.36787313	0.21122473	0.83625653	0.99655376
35569751	column5	5.17917895	2.67678681	1.93484925	0.07693028	0.17584065
35569751	column6	2.13375485	2.06956414	1.03101654	0.32286612	0.71821239
35569751	column7	2.62830487	3.1147511	0.84382501	0.41527216	0.99510151
35569751	column8	-0.2774322	3.67397816	-0.0755128	0.94105105	0.94105105
35569751	GenotypeRiA	1.54308428	2.6909094	0.57344342	0.5769336	0.90739683
35569751	GroupDEX	1.84430605	2.13406464	0.86422221	0.40441019	0.92436616
35569751	GenotypeRiA:GroupDEX	-4.573569	3.18963261	-1.4338858	0.17714508	0.35429015
35569757	DissecterLi	-6.0409305	1.28088219	-4.7162265	0.0005002	0.00800318
35569757	column2	1.73410344	2.2194501	0.78132121	0.44975403	0.8586917
35569757	column3	1.40932062	2.13652383	0.65963253	0.52194842	0.98613518
35569757	column4	-0.6118749	2.01797562	-0.3032122	0.76692051	0.99655376
35569757	column5	4.7639305	2.28124153	2.08830605	0.05875154	0.17584065

35569757	column6	1.90703473	1.76374736	1.0812403	0.30084086	0.71821239
35569757	column7	0.78595373	2.65448842	0.29608482	0.77222679	0.99510151
35569757	column8	-0.3948761	3.13107923	-0.126115	0.90172926	0.94105105
35569757	GenotypeRiA	1.10529196	2.29327725	0.48197049	0.63849789	0.90739683
35569757	GroupDEX	1.93614555	1.81871671	1.06456687	0.30802458	0.92436616
35569757	GenotypeRiA:GroupDEX	-4.5403157	2.71830478	-1.6702747	0.12072066	0.32038294
35569777	DissecterLi	-5.9128347	1.57518337	-3.7537438	0.00275212	0.01544397
35569777	column2	1.84838739	2.72940082	0.67721361	0.51111537	0.8586917
35569777	column3	0.95350029	2.62742104	0.3629035	0.72298874	0.98613518
35569777	column4	0.18272149	2.48163467	0.07362949	0.94251831	0.99655376
35569777	column5	3.70724615	2.80538972	1.32147278	0.21098516	0.37508473
35569777	column6	1.73858263	2.1689938	0.80156182	0.43839111	0.71821239
35569777	column7	-0.4125216	3.26439548	-0.12637	0.90153174	0.99510151
35569777	column8	1.21270926	3.85048991	0.31494934	0.75820932	0.94105105
35569777	GenotypeRiA	1.15939019	2.82019083	0.41110345	0.68824329	0.90739683
35569777	GroupDEX	1.32263196	2.23659315	0.5913601	0.56525284	0.98647054
35569777	GenotypeRiA:GroupDEX	-4.0478628	3.34287457	-1.2108928	0.24924546	0.39879274
35569896	DissecterLi	-3.5159514	0.98519529	-3.5687862	0.00386099	0.01544397
35569896	column2	1.08581152	1.70709829	0.63605683	0.53668231	0.8586917
35569896	column3	1.22718503	1.64331524	0.74677396	0.46957924	0.98613518

35569896	column4	1.14997159	1.55213344	0.74089738	0.47300519	0.99655376
35569896	column5	3.49546275	1.75462539	1.99214189	0.06960758	0.17584065
35569896	column6	1.06202431	1.35659283	0.7828615	0.44888274	0.71821239
35569896	column7	0.0911229	2.04170962	0.04463068	0.96513577	0.99510151
35569896	column8	1.3633579	2.40828121	0.56611242	0.58174985	0.94105105
35569896	GenotypeRiA	1.62774054	1.76388271	0.92281677	0.37428519	0.75204263
35569896	GroupDEX	2.06800662	1.39887271	1.47833795	0.16507982	0.88042569
35569896	GenotypeRiA:GroupDEX	-3.9702416	2.09079422	-1.8989155	0.08187776	0.26200884
35569922	DissecterLi	-2.657857	0.77184449	-3.4435136	0.00486284	0.01556108
35569922	column2	1.18025636	1.33741445	0.88249111	0.39484586	0.8586917
35569922	column3	1.59867431	1.28744406	1.24174274	0.23805442	0.98613518
35569922	column4	0.5017212	1.2160083	0.41259685	0.68717803	0.99655376
35569922	column5	3.52366856	1.37464924	2.56332195	0.02484915	0.1485624
35569922	column6	1.00607148	1.06281336	0.94661163	0.36250929	0.71821239
35569922	column7	0.40942651	1.59956341	0.25596141	0.80231566	0.99510151
35569922	column8	1.93931289	1.88675141	1.02785819	0.32428997	0.86477326
35569922	GenotypeRiA	1.27045592	1.38190182	0.91935324	0.37602131	0.75204263
35569922	GroupDEX	2.4849662	1.09593724	2.26743478	0.04263499	0.36343364
35569922	GenotypeRiA:GroupDEX	-4.4748526	1.6380184	-2.7318696	0.01820337	0.1917606
35570224	DissecterLi	-4.2089386	1.3658612	-3.0815273	0.00951089	0.02536236
35570224	column2	2.38125449	2.36669759	1.00615072	0.33420107	0.8586917

35570224	column3	1.16550446	2.27826965	0.51157441	0.61823156	0.98613518
35570224	column4	1.60093305	2.15185646	0.74397762	0.47120753	0.99655376
35570224	column5	5.70106537	2.43258852	2.34362093	0.0371406	0.1485624
35570224	column6	3.77553071	1.88076165	2.00744773	0.06776336	0.54210686
35570224	column7	0.45716561	2.83059815	0.16150848	0.87438031	0.99510151
35570224	column8	5.17324356	3.33880796	1.54942831	0.14723817	0.86477326
35570224	GenotypeRiA	3.53054796	2.44542274	1.44373728	0.17440895	0.69763581
35570224	GroupDEX	2.39079534	1.93937789	1.23276405	0.24126951	0.92436616
35570224	GenotypeRiA:GroupDEX	-7.4868733	2.89864835	-2.5828843	0.02397008	0.1917606
35578891	DissecterLi	0.85918884	0.45216071	1.90018466	0.08169817	0.16339635
35578891	column2	-1.2992514	0.78348199	-1.6583041	0.1231459	0.8586917
35578891	column3	-0.3973774	0.75420842	-0.5268801	0.60787935	0.98613518
35578891	column4	-0.8455911	0.71236004	-1.1870277	0.25818425	0.99655376
35578891	column5	-1.9919524	0.80529482	-2.4735691	0.0293013	0.1485624
35578891	column6	0.55959442	0.62261562	0.89877992	0.38644937	0.71821239
35578891	column7	-1.4716957	0.93705368	-1.5705564	0.14226627	0.99510151
35578891	column8	-1.429552	1.10529369	-1.2933685	0.22022695	0.86477326
35578891	GenotypeRiA	-1.349931	0.80954351	-1.6675212	0.12127476	0.64679873
35578891	GroupDEX	-1.4331281	0.6420202	-2.2322166	0.0454292	0.36343364

35578891	GenotypeRiA:GroupDEX	2.23598447	0.95958132	2.33016674	0.0380589	0.19972219
35607856	DissecterLi	-0.5348123	0.59831423	-0.8938653	0.38896971	0.51862628
35607856	column2	0.04337732	1.03672968	0.04184053	0.96731394	0.96731394
35607856	column3	-0.8470297	0.9979939	-0.8487323	0.41264124	0.98613518
35607856	column4	0.24963145	0.94261872	0.2648276	0.79563634	0.99655376
35607856	column5	-0.3425403	1.06559314	-0.321455	0.75339566	0.86102361
35607856	column6	-0.4774062	0.82386589	-0.5794707	0.57298979	0.78539297
35607856	column7	-0.0077725	1.23994088	-0.0062685	0.99510151	0.99510151
35607856	column8	-0.2303563	1.46256172	-0.1575019	0.87746842	0.94105105
35607856	GenotypeRiA	-0.5270373	1.07121515	-0.4919994	0.63159689	0.90739683
35607856	GroupDEX	-0.2167496	0.84954268	-0.2551368	0.80293771	0.98647054
35607856	GenotypeRiA:GroupDEX	0.63433338	1.26975021	0.49957336	0.62640909	0.70415917
35607904	DissecterLi	0.94430893	0.48974789	1.92815312	0.07783091	0.16339635
35607904	column2	0.8154383	0.84861124	0.96090915	0.35556071	0.8586917
35607904	column3	0.27789479	0.81690421	0.34018039	0.73960139	0.98613518
35607904	column4	1.14149108	0.77157706	1.4794259	0.16479348	0.87889854
35607904	column5	0.43159464	0.87223731	0.49481331	0.6296671	0.83955613
35607904	column6	0.27715447	0.67437237	0.41098135	0.68833042	0.81223671

35607904	column7	1.53789048	1.01494902	1.51523915	0.15560082	0.99510151
35607904	column8	-1.0177876	1.19717448	-0.8501582	0.41187893	0.94105105
35607904	GenotypeRiA	0.25303754	0.8768392	0.28857918	0.77782768	0.90739683
35607904	GroupDEX	0.01236157	0.69539001	0.01777646	0.98610933	0.98647054
35607904	GenotypeRiA:GroupDEX	0.44331655	1.03934933	0.42653277	0.67727141	0.70415917

4. References

1. Bruckl TM, Spoormaker VI, Samann PG, Brem AK, Henco L, Czamara D, et al. The biological classification of mental disorders (BeCOME) study: a protocol for an observational deep-phenotyping study for the identification of biological subtypes. BMC Psychiatry. 2020;20(1):213.
2. Kopf-Beck J, Zimmermann P, Egli S, Rein M, Kappelmann N, Fietz J, et al. Schema therapy versus cognitive behavioral therapy versus individual supportive therapy for depression in an inpatient and day clinic setting: study protocol of the OPTIMA-RCT. BMC Psychiatry. 2020;20(1):506.
3. Wittchen H-U PH. DIA-X-Interviews: Manual für Screening-Verfahren und Interview; Interviewheft. 1997.
4. World Health Organization(WHO). The ICD-10 classification of mental and behavioural disorders: World Health Organization; 1993.
5. American Psychiatric Association. Diagnostic and statistical manual of mental disorders (4th ed.). 1994.
6. Wiechmann T, Roh S, Sauer S, Czamara D, Arloth J, Kodel M, et al. Identification of dynamic glucocorticoid-induced methylation changes at the FKBP5 locus. Clin Epigenetics. 2019;11(1):83.
7. Yusupov N, Dieckmann L, Erhart M, Sauer S, Rex-Haffner M, Kopf-Beck J, et al. Transdiagnostic evaluation of epigenetic age acceleration and burden of psychiatric disorders. Neuropsychopharmacology. 2023.
8. Aryee MJ, Jaffe AE, Corrada-Bravo H, Ladd-Acosta C, Feinberg AP, Hansen KD, et al. Minfi: a flexible and comprehensive Bioconductor package for the analysis of Infinium DNA methylation microarrays. Bioinformatics. 2014;30(10):1363-9.
9. Maksimovic J, Phipson B, Oshlack A. A cross-package Bioconductor workflow for analysing methylation array data. F1000Res. 2016;5:1281.
10. Touleimat N, Tost J. Complete pipeline for Infinium((R)) Human Methylation 450K BeadChip data processing using subset quantile normalization for accurate DNA methylation estimation. Epigenomics. 2012;4(3):325-41.

11. Teschendorff AE, Marabita F, Lechner M, Bartlett T, Tegner J, Gomez-Cabrero D, et al. A beta-mixture quantile normalization method for correcting probe design bias in Illumina Infinium 450 k DNA methylation data. Bioinformatics. 2013;29(2):189-96.
12. Leek JT, Johnson WE, Parker HS, Jaffe AE, Storey JD. The sva package for removing batch effects and other unwanted variation in high-throughput experiments. Bioinformatics. 2012;28(6):882-3.
13. Guintivano J, Aryee MJ, Kaminsky ZA. A cell epigenotype specific model for the correction of brain cellular heterogeneity bias and its application to age, brain region and major depression. Epigenetics. 2013;8(3):290-302.
